# Macroevolutionary Dynamics and Historical Biogeography of Primate Diversification Inferred from a Species Supermatrix

**DOI:** 10.1371/journal.pone.0049521

**Published:** 2012-11-16

**Authors:** Mark S. Springer, Robert W. Meredith, John Gatesy, Christopher A. Emerling, Jong Park, Daniel L. Rabosky, Tanja Stadler, Cynthia Steiner, Oliver A. Ryder, Jan E. Janečka, Colleen A. Fisher, William J. Murphy

**Affiliations:** 1 Department of Biology, University of California Riverside, Riverside, California, United States of America; 2 Department of Biology and Molecular Biology, Montclair State University, Montclair, New Jersey, United States of America; 3 Department of Biology, University of Washington, Seattle, Washington, United States of America; 4 Department of Integrative Biology, University of California, Berkeley, California, United States of America; 5 Department of Ecology and Evolutionary Biology, University of Michigan, Ann Arbor, Michigan, United States of America; 6 Institut für Integrative Biologie, Eidgenössiche Technische Hochschule Zurich, Zurich, Switzerland; 7 San Diego Zoo Institute for Conservation Research, San Diego Zoo Global, San Diego, California, United States of America; 8 Department of Veterinary Integrative Biosciences, Texas A&M University, College Station, Texas, United States of America; University of Florence, Italy

## Abstract

Phylogenetic relationships, divergence times, and patterns of biogeographic descent among primate species are both complex and contentious. Here, we generate a robust molecular phylogeny for 70 primate genera and 367 primate species based on a concatenation of 69 nuclear gene segments and ten mitochondrial gene sequences, most of which were extracted from GenBank. Relaxed clock analyses of divergence times with 14 fossil-calibrated nodes suggest that living Primates last shared a common ancestor 71–63 Ma, and that divergences within both Strepsirrhini and Haplorhini are entirely post-Cretaceous. These results are consistent with the hypothesis that the Cretaceous-Paleogene mass extinction of non-avian dinosaurs played an important role in the diversification of placental mammals. Previous queries into primate historical biogeography have suggested Africa, Asia, Europe, or North America as the ancestral area of crown primates, but were based on methods that were coopted from phylogeny reconstruction. By contrast, we analyzed our molecular phylogeny with two methods that were developed explicitly for ancestral area reconstruction, and find support for the hypothesis that the most recent common ancestor of living Primates resided in Asia. Analyses of primate macroevolutionary dynamics provide support for a diversification rate increase in the late Miocene, possibly in response to elevated global mean temperatures, and are consistent with the fossil record. By contrast, diversification analyses failed to detect evidence for rate-shift changes near the Eocene-Oligocene boundary even though the fossil record provides clear evidence for a major turnover event (“Grande Coupure”) at this time. Our results highlight the power and limitations of inferring diversification dynamics from molecular phylogenies, as well as the sensitivity of diversification analyses to different species concepts.

## Introduction

Primates comprise an ecologically, morphologically, and taxonomically diverse order of mammals. The oldest stem primates (i.e., “Plesiadapiformes”) are from the earliest Paleocene of North America [1], whereas the fossil record of crown primates begins with the appearance of taxa in Western North America, Western Europe, Africa, and Asia at or near the Paleocene/Eocene boundary [1], [2]. Extant primates are widely distributed throughout tropical and subtropical regions of Africa, Madagascar, Asia, and the New World, although the fossil record is depauperate in several regions where extant primate diversity is highest including Madagascar, the Congo Basin, Southeast Asia, and the Amazon Basin [3].

Groves [4] recognized 233 primate species in his 1993 classification. Since that time, the number of primate species has increased steadily, with 354 and 376 species in Groves’ 2001 [5] and 2005 [6] classifications, respectively. We estimate that the number of living primate species is at least 450 when Groves’ [6] classification is augmented with newly recognized species (Text S1). The increase in the number of primate species has been driven by widespread adoption of the phylogenetic species concept by primate taxonomists and the application of genetic methods that allow for the discovery of young evolutionary lineages that would otherwise go undetected [5]–[11].

Analyses of phylogenetic relationships and divergence times among primate species are critical for understanding the evolutionary and biogeographic history of this group, including patterns of diversification in relationship to environmental changes throughout the Cenozoic and the role of dispersal to previously uncolonized areas in promoting diversification. Accurate phylogenies also provide an essential framework for informing decisions in conservation biology and understanding the emergence of zoonotic diseases [12].

Purvis [13] provided the first comprehensive estimate of phylogenetic relationships among living primates based on supertree methods and included all 203 species that were recognized by Corbett and Hill [14]. The most comprehensive analyses of primate relationships based on molecular supermatrices are those of Chatterjee et al. [15], Fabre et al. [3], and Perelman et al. [12], who included 219, 271, and 186 primate species, respectively. Fabre et al.’s [3] data set included the greatest number of primate species, but Perelman et al.’s [12] data matrix comprised the largest number of gene loci (54 nuclear segments) and had the lowest percentage (18) of missing data. The majority of nodes were recovered with 100% bootstrap support in Perelman et al.’s [12] analysis, although these authors only included 41–49% of extant primate species if we allow that there are 376–450 living species.

Recent studies have also estimated divergence times among primate species based on relaxed clock methods [3], [12], [15]. Molecular divergence estimates are in good agreement for some nodes (e.g., Simiiformes, Catarrhini, Platyrrhini) but show more disagreement deeper in the tree (e.g., Primates, Strepsirrhini, Haplorhini). For example, relaxed clock estimates for the base of primates range from 63.7 Ma [15] to 87.2 Ma [12]. These differences have profound implications for interpreting the history of primate diversification in relationship to important events in earth history such as the Cretaceous-Paleogene (KPg) mass extinction.

The biogeographic history of Primates is also contentious. Beard [16] suggested that the common ancestor of living primates was Asian; Silcox [2] suggested that Africa, Asia, Europe, and North America are all possible places of origin; and Bloch et al. [1] concluded that the common ancestor of living primates was either Asian or North American. All of these studies used Fitch parsimony to reconstruct ancestral areas, but this approach has shortcomings when applied to ancestral area reconstructions [17], [18].

In the present study we assembled a molecular supermatrix, primarily from previously published GenBank sequences, for 367 extant primate species and reconstructed phylogenetic relationships using maximum likelihood. We also performed timetree analyses with relaxed clock methods and a more comprehensive assemblage of calibrations than previous studies. Ancestral area reconstructions were performed with methods that were developed specifically for historical biogeography [17]–[19], and the resulting ancestral area chronograms were used as a framework for understanding the history of trans-continental dispersal in the ancestors of primates with extant descendants. Finally, we performed diversification analyses to determine if a molecular phylogeny for primates retains the signature of historical events that have increased or decreased diversification rates, and ascertained if diversification rate changes are sensitive to the application of the phylogenetic species concept.

## Results

### Primate Supermatrix

The concatenated data set included 372 taxa (367 primates, five outgroups) and was 61,199 base pairs (bp) after excluding ambiguous regions of the alignment for the mitochondrial *12S* and *16S rRNA* genes. Among the 22,766,028 cells in the data matrix, 7,140,500 (31.4%) are filled by nucleotides and the remainder are either missing or gaps (68.6%). The mean number of nucleotides per taxon is 19,194.9. Taxon completeness for the concatenated data set ranged from 34 taxa (*ABO*) to 276 taxa (cytochrome b), with a mean of 138.3 taxa per locus. Gene completeness for the concatenated data set ranged from one locus per taxon to 77 loci per taxon with a mean of 29.4 loci per taxon. The nuclear data set included 243 taxa and 51,801 bp. Mean completeness for the nuclear data set was 136 taxa per gene segment (taxon completeness) and 25.1 loci per taxon (gene completeness). The mitochondrial data set included 355 taxa, 9398 bp, and mean completeness of 154.2 taxa per mitochondrial gene (taxon completeness) and 4.1 mitochondrial genes per taxon (gene completeness). In almost all cases where a taxon was represented by a single locus, the singleton was a mitochondrial gene (cytochrome b, cytochrome oxidase II, *12S rRNA*, or *16S rRNA*) with overlapping sequences among congeners in the data set. The only exception is *Gorilla beringei*, which is represented by a *CXCR5* sequence that shares both congeneric (*G. gorilla*) and confamilial (*Homo sapiens, Pan troglodytes*) overlap.

### Phylogenetic Analyses

**Figure 1:**
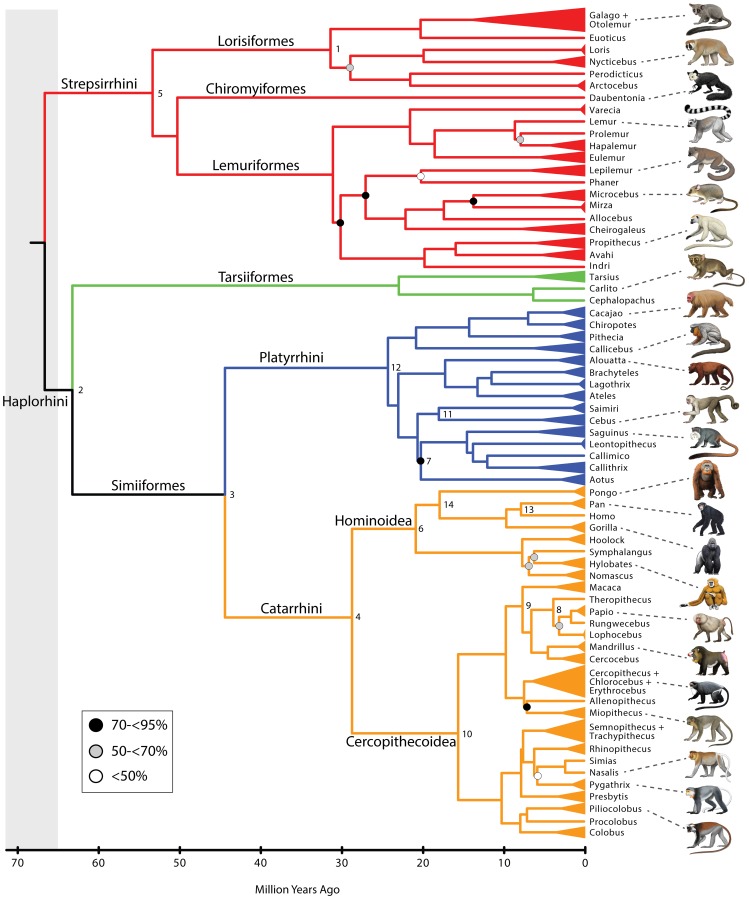
A timetree for 70 primate genera based on mcmctree with autocorrelated rates and soft-bounded constraints. The tree was rooted with five outgroups from Lagomorpha, Scandentia, and Dermoptera (not shown). All nodes without filled circles were recovered with≥95% bootstrap support in maximum likelihood analyses with RAxML. Black, gray, and white filled circles indicate nodes that were recovered with 70 to<95% bootstrap support, 50 to<70% bootstrap support, and<50% bootstrap support, respectively. The full timetree with 367 primate species and five outgroups is provided in Text S2. Also see Figure 2 (strepsirrhines), Figure 3 (tarsiiforms+platyrrhines), Figure 4 (cercopithecoid), and Figure 5 (hominoids). Calibrated nodes are indicated with numbers and are cross-referenced to Text S3. Paintings by Carl Buell.

**Figure 2:**
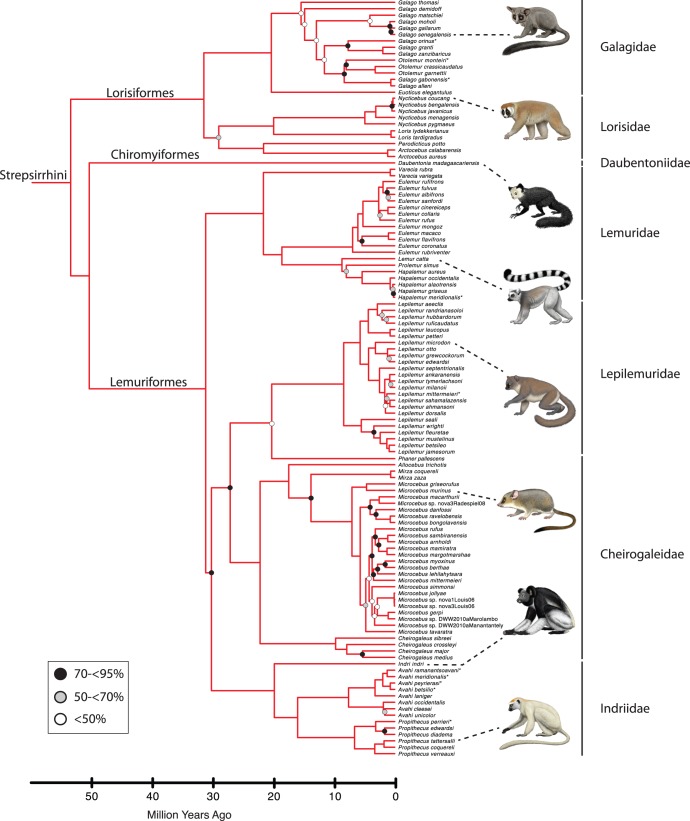
Strepsirrhine portion of mcmctree timetree (autocorrelated rates, soft-bounded constraints). Nodes without filled circles and nodes with black, gray, and white filled circles are as in Figure 1. Primate species that were represented by a single locus in the RAxML analysis with the combined (nuclear+mitochondrial) data set are denoted with asterisks. Paintings by Carl Buell.

**Figure 3:**
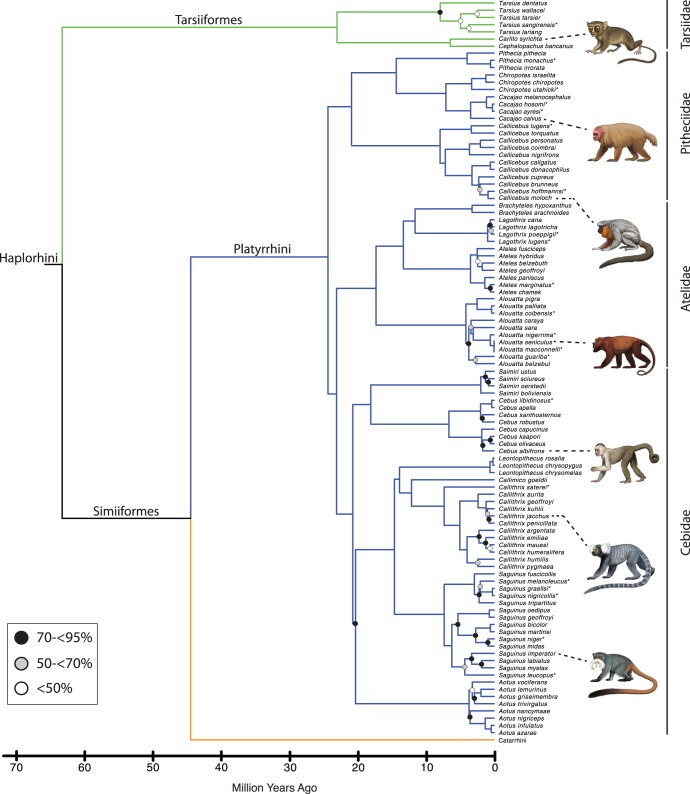
Tarsiiform and platyrrhine portion of mcmctree timetree (autocorrelated rates, soft-bounded constraints). Nodes without filled circles and nodes with black, gray, and white filled circles are as in Figure 1. Primate species that were represented by a single locus in the RAxML analysis with the combined (nuclear+mitochondrial) data set are denoted with asterisks. Paintings by Carl Buell.

**Figure 4:**
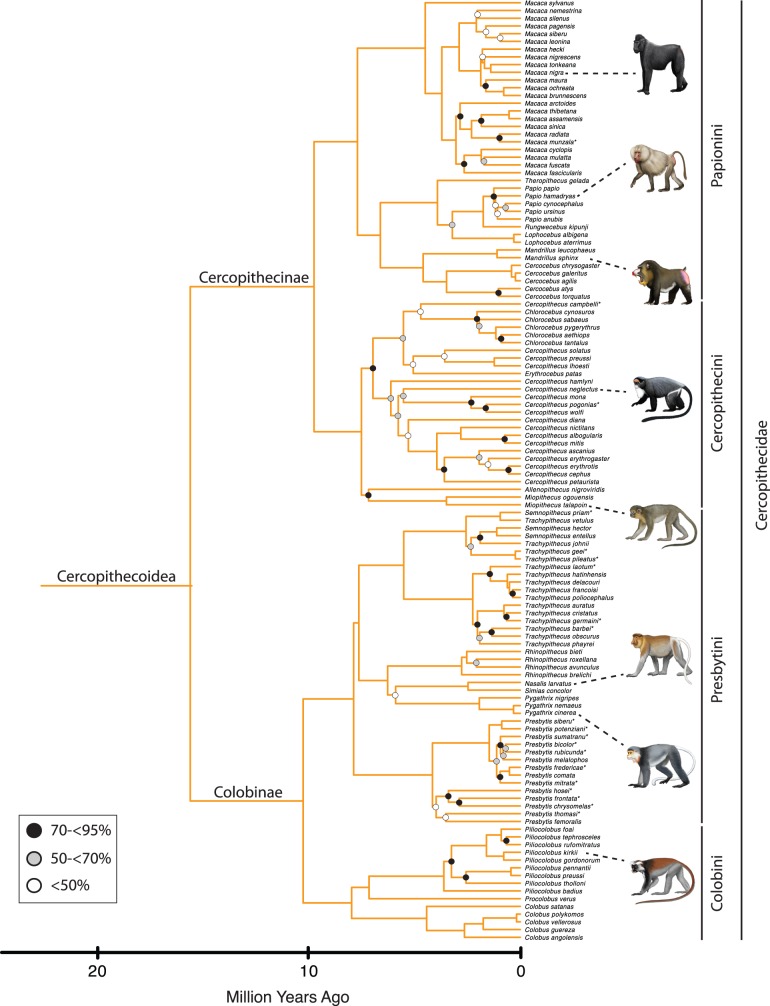
Cercopithecoid portion of mcmctree timetree (autocorrelated rates, soft-bounded constraints). Nodes without filled circles and nodes with black, gray, and white filled circles are as in Figure 1. Primate species that were represented by a single locus in the RAxML analysis with the combined (nuclear+mitochondrial) data set are denoted with asterisks. Paintings by Carl Buell.

**Figure 5:**
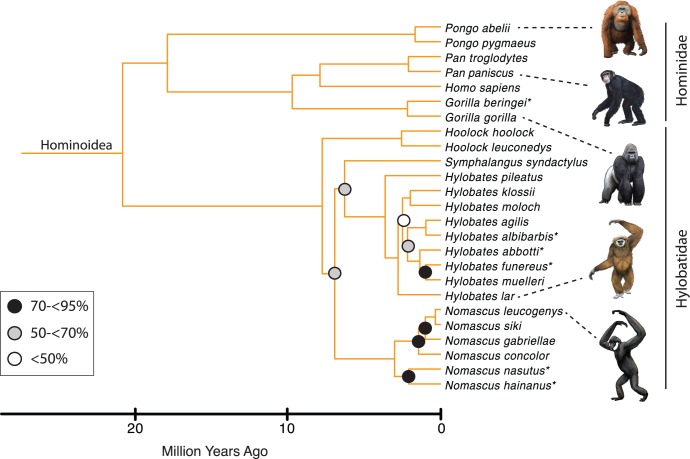
Hominoid portion of mcmctree timetree (autocorrelated rates, soft-bounded constraints). Nodes without filled circles and nodes with black, gray, and white filled circles are as in Figure 1. Primate species that were represented by a single locus in the RAxML analysis with the combined (nuclear+mitochondrial) data set are denoted with asterisks. Paintings by Carl Buell.

Figure 1 shows the maximum likelihood bootstrap tree for 70 primate genera that were included in our analysis. Species level trees for Strepsirrhini, Tarsiiformes+Platyrrhini, Cercopithecoidea, and Hominoidea are shown in Figures 2, 3, 4, and 5, respectively. The ML phylogram for 367 primate species is provided in Text S2. Primate suborders (Haplorhini, Strepsirrhini), infraorders (Simiiformes, Tarsiiformes, Lemuriformes, Lorisiformes), parvorders (Catarrhini, Platyrrhini), and superfamilies (Hominoidea, Cercopithecoidea) (*sensu* [12]) were recovered with 99–100% bootstrap support. All primate families were recovered as monophyletic with 100% bootstrap support except for Cebidae (99%), Lorisidae (63%), and Cheirogaleidae (56%). Bootstrap support for most primate genera was 100%, but in a few cases support was slightly lower (*Hapalemur* = 99%, *Miopithecus* = 99%, *Callithrix* = 98%, *Saguinus* = 98%, *Chlorocebus* = 90%) or genera were not recovered as monophyletic (*Trachypithecus*, *Semnopithecus*, *Cercopithecus*, *Galago*). Mean bootstrap support percentages for all primate nodes, primate nodes at and above the level of genus, and primate nodes below the level of genus were 86.2%, 95.5%, and 82.1%, respectively.

### Timetree Analyses

Divergence times for 367 primates based on analyses with autocorrelated rates and soft-bounded constraints are shown in Figures 1, 2, 3, 4, 5. Mean divergence dates and composite 95% credibility intervals for key nodes are provided in Table 1. Median dates, mean dates, and 95% credibility intervals for analyses with autocorrelated rates and hard-bounded constraints (AUTOhard), autocorrelated rates and soft-bounded constraints (AUTOsoft), independent rates and hard-bounded constraints (IRhard), and independent rates and soft-bounded constraints (IRsoft) are given in Table S1 for all primate nodes (numbered in Fig. S1). Newick timetrees based on each analysis are provided in Text S2.

**Table 1:** Mean divergence dates and 95% composite credibility intervals in millions of years for select nodes based on four mcmctree chronograms (autocorrelated rates with hard-bounded constraints, autocorrelated rates with soft-bounded constraints, independent rates with hard-bounded constraints, independent rates with soft-bounded constraints).

Clade	Mean DivergenceDate	Composite 95%Credibility Minimum	Composite 95%Credibility Maximum
Primates	67.84	60.99	76.72
Strepsirrhini	54.23	48.75	57.22
Lorisiformes	34.74	27.9	38.24
Galagidae	22.67	16.2	32.22
*Galago*+*Otolemur*	15.08	11.18	21.06
Lorisidae	32.28	25.58	36.31
*Loris*	0.43	0	2.56
*Nycticebus*	5.27	3.54	7.91
*Arctocebus*	0.83	0.04	3.51
Lemuriformes+Chiromyiformes	49.96	45.16	53.78
Lemuriformes	31.77	26.73	36.64
Lemuridae	20.73	15.28	25.97
*Varecia*	0.70	0.28	1.68
*Hapalemur*	4.09	1.54	8.1
*Eulemur*	6.21	4.21	9.47
Indriidae+Cheirogaleidae+Lepilemuridae	30.65	25.62	35.45
Lepilemuridae	8.51	6.15	12.71
Cheirogaleidae (w/o *Phaner*)	21.84	17.39	26.62
*Mirza*	0.50	0.05	2.13
*Microcebus*	6.92	4.97	8.81
*Cheirogaleus*	9.34	4.86	17.77
Indriidae	18.45	11.71	25.51
*Avahi*	6.45	2.72	11.37
*Propithecus*	6.04	3.93	9.39
Haplorhini	61.16	57.62	69.59
Tarsiiformes	18.64	8.75	37.19
*Tarsius*	5.14	1.29	13.52
Simiiformes	40.60	33.55	49.48
Platyrrhini	23.32	19.26	27.49
Pitheciidae	19.85	15.97	23.83
*Cacajao*	2.90	1.6	4.42
*Chiropotes*	2.67	1.1	5.03
*Pithecia*	3.46	1.97	6.01
*Callicebus*	7.64	5.16	10.38
Atelidae	15.17	10.94	20.18
*Alouatta*	3.68	2.44	5.15
*Brachyteles*	2.65	1.3	5.12
*Lagothrix*	1.038	0.34	2.89
*Ateles*	2.82	1.58	4.43
Cebidae	19.73	16.07	23.56
*Saimiri*+*Cebus*	17.25	13.76	20.79
*Saimiri*	1.75	1.06	2.88
*Cebus*	5.7	3.55	8.33
*Saguinus*	6.65	4.82	8.69
*Leontopithecus*	0.52	0.21	1.1
*Callithrix*	6.51	3.81	11.44
*Aotus*	3.31	2.09	4.43
Catarrhini	25.07	19.67	32.83
Hominoidea	17.36	12.44	23.9
Hominidae	15.09	11.04	20.8
*Pongo*	1.28	0.51	2.99
Homininae	8.03	5.53	11.66
*Homo*+*Pan*	6.66	4.74	9.5
*Pan*	1.62	0.67	3.5
*Gorilla*	1.69	0.01	4.64
Hylobatidae	6.68	3.92	9.73
*Hoolock*	1.96	0.22	4.4
*Hylobates*	2.99	1.62	4.68
*Nomascus*	2.66	1.22	4.19
Cercopithecidae	13.17	8.93	18.27
Cercpithecinae	8.35	5.41	11.6
Papionini	6.47	3.94	9.13
*Macaca*	3.8	2.34	5.47
*Papio*	1.07	0.55	1.66
*Lophocebus*	0.24	0.01	0.65
Cercopithecini	6.15	3.78	8.96
*Cercopithecus*+*Cercocebus*+*Erythrocebus*	5.68	3.45	8.36
*Miopithecus*	2.96	0.85	6.73
Colobinae	8.85	6.25	12.04
Presbytini	6.69	4.59	9.29
*Trachypithecus*+*Semnopithecus*	4.17	2.08	6.84
*Rhinopithecus*	2.54	1.27	3.79
*Pygathrix*	1.49	0.59	3
*Presbytis*	3.81	2.14	5.73
Colobini	6.83	4.68	9.46
*Piliocolobus*	3.41	1.97	4.66
*Colobus*	3.79	1.99	6.13

Median dates, mean dates, and 95% credibility intervals for individual chronograms are given in Table S2.

Mean divergence dates based on four molecular dating analyses ranged from 71.4−62.8 Ma for Primates, 55.3−53.3 Ma for Strepsirrhini, and 64.2−58.4 for Haplorhini (Table S1). Analyses with hard-bounded constraints provided younger dates (58.6−58.5) for Haplorhini than did soft-bounded analyses (64.2−63.3 Ma). Within Strepsirrhini, the basal split among Malagasy taxa (Chiromyiformes to Lemuriformes) was dated at 50.5−49.2 Ma whereas Galagidae and Lorisidae separated from each other at 37.4−31.4 Ma. The base of Tarsiiformes was dated at 24.9−23.0 Ma in analyses with autocorrelated rates but only 13.5−13.2 Ma in analyses with independent rates. Similarly, molecular dates based on autocorrelated rates were consistently older than dates based on independent rates for Simiiformes (44.5−42.8 versus 37.7−37.4 Ma) and most of its constituent clades including Catarrhini (28.8−27.7 versus 22.5−21.3 Ma), Cercopithecidae (15.7−15.6 versus 11.4−10.1 Ma), Hominoidea (20.9−20.2 versus 14.7−13.6 Ma), Hominidae (18.0−17.3 versus 13.0−12.1 Ma), Hylobatidae (7.8 versus 6.0−5.2 Ma), Platyrrhini (24.3−24.0 versus 23.4−21.5 Ma), Cebidae (20.7−20.6 versus 19.6−18.1 Ma), Pitheciidae (20.9−20.7 versus 19.7−18.1 Ma), and Atelidae (17.3 versus 13.6−12.5 Ma). Among monophyletic genera the oldest basal divergences were in *Cheirogaleus* (11.6−7.6 Ma) and *Lepilemur* (10.2−7.3 Ma), whereas basal divergence dates were less than one million years in all analyses for *Loris*, *Varecia*, *Mirza*, *Leontopithecus*, and *Lophocebus* (Figs. 2, 3, 4, Table S1).

Using soft bounds, six timetree dates were either younger than the minimum date or older than the maximum date. AUTOsoft and IRsoft analyses returned dates of 31.4 and 32.8 Ma for Lorisiformes, respectively, both of which are younger than the minimum age of 37.1 Ma. The maximum age of Haplorhini (58.9 Ma) was also violated in both the AUTOsoft (63.3 Ma) and IRsoft (64.2 Ma) analyses. Finally, IRsoft analyses returned dates for *Macaca* to other Papionini (4.5 Ma) and *Theropithecus* to *Papio*+*Rungwecebus*+*Lophocebus* (2.4 Ma) that were slightly younger than minimum constraints for these clades (5.5 and 3.5 Ma, respectively).

### Ancestral-Area Reconstructions

**Figure 6:**
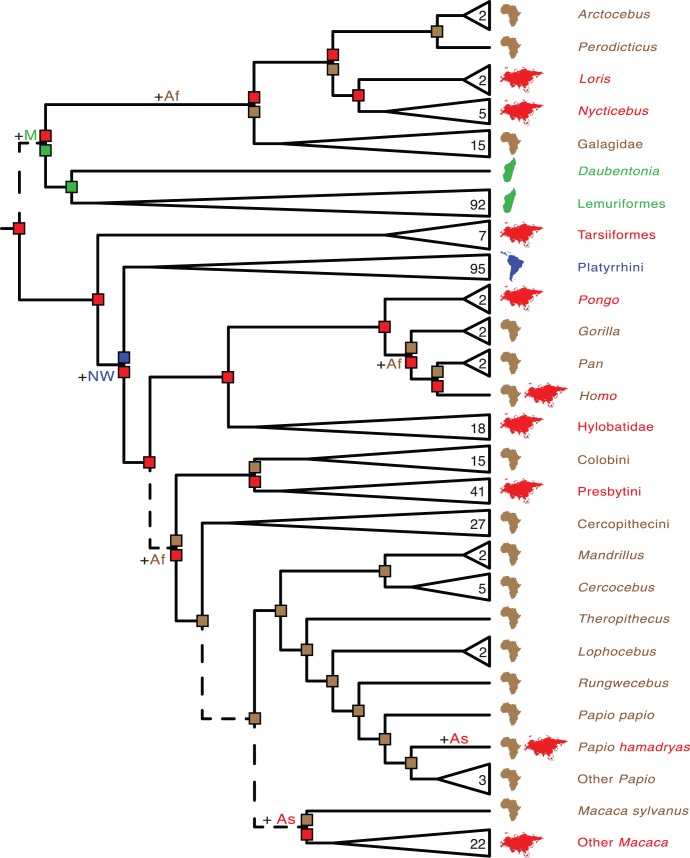
Ancestral area reconstructions for extant primates based on dispersal-extinction-cladogenesis (DEC). Ancestral area reconstructions were performed under a simplified DEC model that allowed a maximum of two areas at internal nodes. Internal nodes with a single square were reconstructed to include a single area, whereas internal nodes with two squares were reconstructed to include two areas. Dashed lines between adjacent nodes indicate that alternative area reconstructions at the basal-most node fall within two log-likelihood units of the optimal scenario shown in the figure. Multi-colored names denote taxa that occur in more than one area. Range transitions on branches indicate inferred dispersal events. Numbers in triangles indicate the number of species in each collapsed clade.

**Figure 7:**
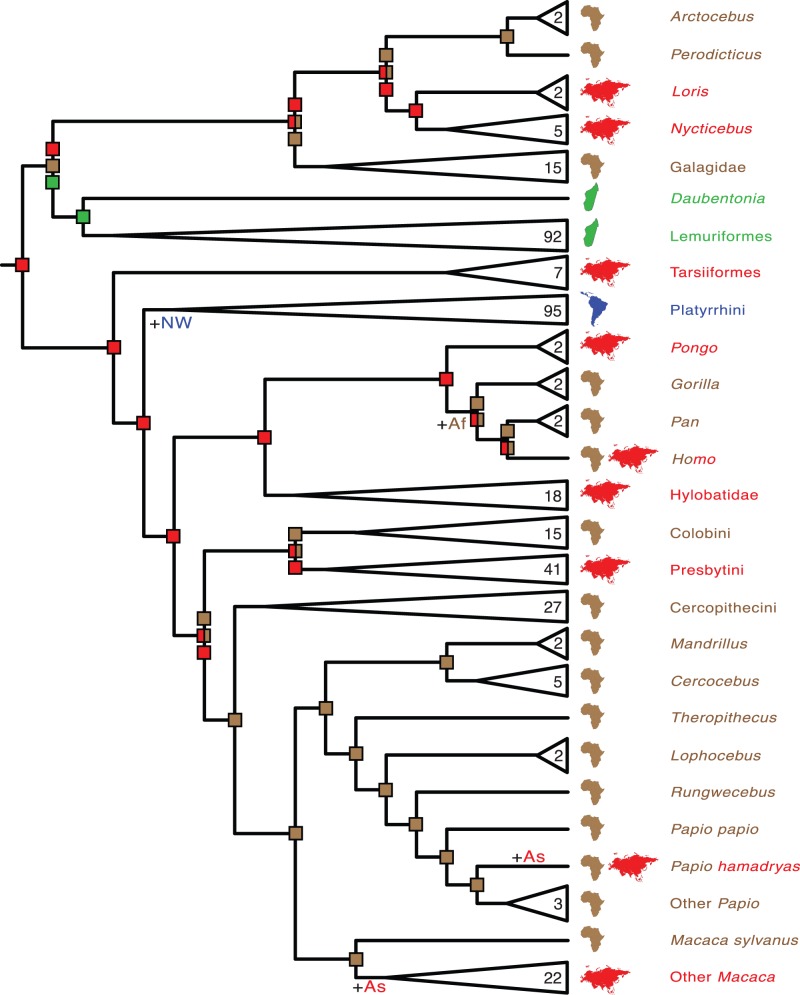
Ancestral area reconstructions for extant primates based on minimum area change (MAC) parsimony. Nodes with unambiguous ancestral area reconstructions are shown with a single colored square; nodes with ambiguous reconstructions are shown with two or more squares, and each colored square corresponds to a different reconstruction. Bi-colored squares indicate reconstructions that included two areas. Multi-colored names denote taxa that occur in more than one area. Range transitions on branches indicate inferred dispersal events. Numbers in triangles indicate the number of species in each collapsed clade.

The results of dispersal-extinction-cladogenesis (DEC) [17], [19] and minimum area change (MAC) parsimony [18] analyses are summarized in Figures 6 and 7, respectively, and in Table 2. Asia was reconstructed as the ancestral area of Primates, Haplorhini, Catarrhini, and Hominoidea by both methods. The ancestral area of Strepsirrhini was reconstructed as Asia+Madagascar by DEC (Fig. 6) and Asia or Africa or Madagascar by MAC parsimony (Fig. 7). Lorisiformes was reconstructed to have an ancestral area that included both Asia and Africa by DEC (Fig. 6) and one or both of these areas by MAC parsimony (Fig. 7). The ancestral area for Simiiformes was either Asia+New World (DEC) (Fig. 6) or Asia (MAC parsimony) (Fig. 7).

**Table 2:** Results of ancestral area reconstructions with minimum area change (MAC) parsimony and eight topologies from Seiffert et al.

Seiffert et al. [20] Topology	Crown Primates		Strepsirrhini		Haplorhini		Stem Primates	
	5 areas	6 areas	5 areas	6 areas	5 areas	6 areas	5 areas	6 areas
Figure 2A	EA	NA, EU, AS, NA+E, EU+AS	AF	AF	EA	NA, EU, AS, NA+EU	EA	NA, EU, AS
Figure 2B	EA	AS	AF, EA, MA	AF, EU, MA, AS	EA	AS	EA	AS
Figure 2C	EA	AS	MA	MA	EA	AS	EA	AS
Figure 2D	EA	AS	MA	MA	EA	AS	EA	AS
Figure S2A	EA	NA, EU, AS, NA+EU, NA+AS, EU+AS	MA	MA	EA	NA, EU, AS, NA+EU	EA	NA, EU, AS, NA+AS, EU+AS
Figure S2B	EA	NA, EU, AS, EU+AS	AF, EA, MA	NA, AF, EU, MA, AS	EA	NA, EU, AS, EU+AS	EA	NA, EU, AS, EU+AS
Figure S2C	EA	NA, EU, AS, NA+EU, NA+AS, EU+AS	MA	MA	EA	NA, EU, AS, NA+EU	EA	NA, EU, AS, NA+AS, EU+AS
Figure S2D	EA	NA, EU, AS, NA+EU, NA+AS, EU+AS	AF, EA, MA	AF, EU, MA, AS	EA	NA, EU, AS, NA+EU	EA	NA, EU, AS, NA+AS, EU+AS

Abbreviations: AF = Africa; AS = Asia; EA = Eurasia; EU = Europe; MA = Madagascar; NA = North America.

[20]. Areas delimited by commas indicate alternate reconstructions.

DEC results suggest that there have been seven dispersal events between the four areas: one dispersal to Madagascar in the ancestor of Strepsirrhini, one dispersal to the New World in the ancestor of Simiiformes, three dispersals to Africa (ancestors of Lorisiformes, hominids, and cercopithecids, respectively), and two dispersals to Asia (ancestors of *Macaca* and *Papio hamadryas*, respectively) (Fig. 6), although we note that the occurrence of *P. hamadryas* in Asia is restricted to the Arabian Peninsula. Other changes in area are the result of range inheritance at cladogenic events. MAC parsimony results suggest that there have been 18 gains or losses of area in the evolutionary history of extant primates lineages, with a minimum of seven gains of area (Madagascar, New World, Asia × 2, Africa × 3) and a maximum of ten gains of area (Madagascar, New World, Asia 3 to 5 times, Africa 3 to 5 times). However, the only gains or losses of area that were reconstructed unambiguously were gain of the New World in the ancestor of Platyrrhini, gain of Africa in the ancestor of Homininae, gain of Asia in *Papio hamadryas*, and gain of Asia in the common ancestor of Asian *Macaca* (Fig. 7).

MAC parsimony analyses with a combination of extinct and extant taxa and eight different topologies from Seiffert et al. [20] are summarized in Table 2. Analyses with five areas (North America, Eurasia, Africa, Madagascar, South America) suggest that the most recent common ancestor of crown primates resided in Eurasia. Analyses with six areas (same as above but with Eurasia split into Europe and Asia) reconstructed the common ancestor of crown primates as Asian or were equivocal and recovered different combinations of Asia, Europe, North America, North America+Europe, North America+Asia, and Europe+Asia as the ancestral area for crown primates. Analyses with five different areas reconstructed Eurasia as the common ancestral area of Haplorhini whereas analyses with six areas either reconstructed Asia as the sole area for this clade or were equivocal and suggested that the common ancestor of Haplorhini occurred in North America, Europe, Asia, or North America+Europe. The most recent common ancestor of Strepsirrhini was reconstructed as African, Madagascan, or equivocal (at least three of North America, Africa, Europe, Madagascar, Asia). All of the analyses that reconstructed Madagascar as the exclusive area of Strepsirrhini were based on topologies with paraphyletic Lemuriformes. Finally, the ancestral area for crown+stem primates was reconstructed as Eurasian in analyses with five areas and Asian or equivocal (four or more of North America, Europe, Asia, North America+Asia, Europe+Asia) in analyses with six areas.

### Diversification Analyses

**Figure 8:**
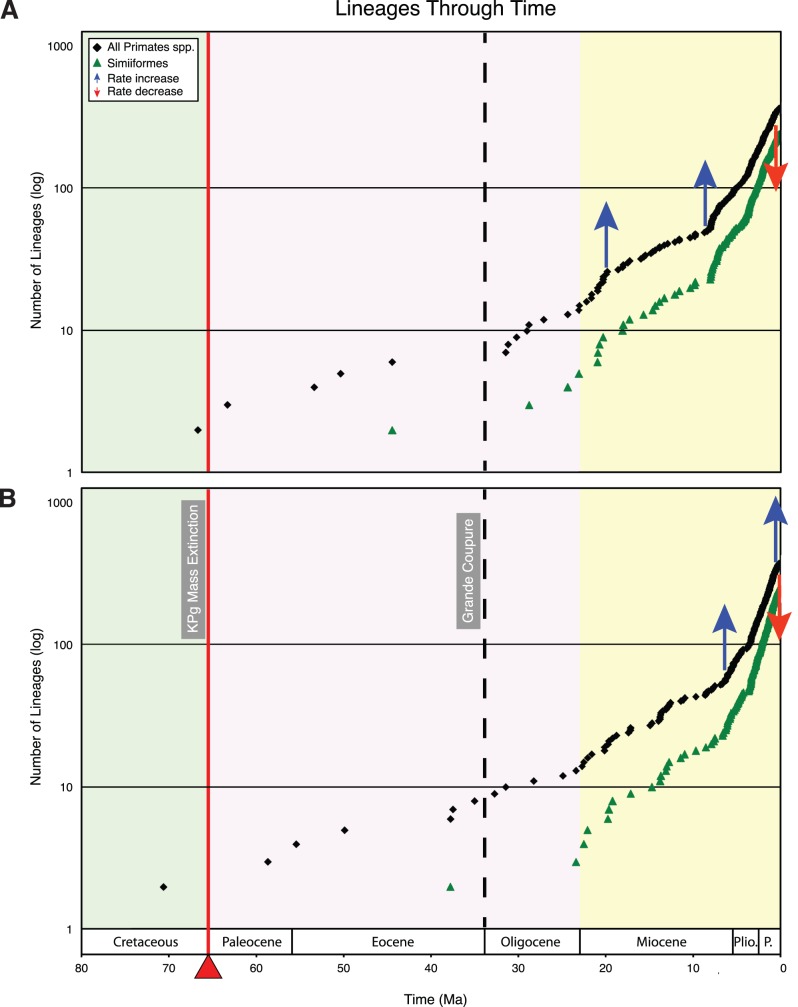
Lineage through time (LTT) plots for Primates and Simiiformes based on Groves05+taxon sampling. (A) LTT plot based on timetree with autocorrelated rates and soft-bounded constraints. (B) LTT plot based on timetree with independent rates and hard-bounded constraints. Arrows denote statistically significant rate increases and decreases that were detected with TreePar [120] (see Table 3). Green background = Cretaceous; pink background = Paleogene; yellow background = Neogene.

The results of 12 diversification analyses with TreePar are summarized in Table 3. Figure 8 shows representative lineage through time plots for analyses with Groves05+taxon sampling. Eleven of 12 diversification analyses supported a significant rate increase in the late Miocene/Pliocene (mean = 7.0 Ma). Analyses with the AUTOsoft timetree, whether with Groves93, Groves95, or Groves05+taxon sampling, supported a second rate increase at 19.8 Ma. Two analyses (IRhard and IRsoft with Groves05+taxon sampling) supported a second rate increase in the Pleistocene (0.6−0.5 Ma). Finally, 12/12 analyses supported a rate decrease in the Pleistocene between 1.9 and 0.1 Ma (mean = 0.6 Ma). Analyses based on the same timetree but with alternate taxon sampling (Groves93, Groves05, Groves05+) showed only minor differences, whereas differences were more apparent in analyses with different timetrees (i.e., AUTOhard, AUTOsoft, IRhard, IRsoft) and equivalent taxon sampling.

**Table 3:** Diversification rate shifts detected by TreePar [120] with different combinations of four timetrees and three taxonomies.

	Groves93 Taxonomy	Groves05 Taxonomy	Groves05+Taxonomy
AUTOhard Timetree	8.0 Ma (increase) 1.9 Ma (decrease)	8.0 Ma (increase) 0.5 Ma (decrease)	8.0 Ma (increase) 0.5 Ma (decrease)
AUTOsoft Timetree	19.8 Ma (increase) 8.7 Ma (increase) 1.7 Ma (decrease)	19.8 Ma (increase) 8.1 Ma (increase) 0.5 Ma (decrease)	19.8 Ma (increase) 8.5 Ma (increase) 0.5 Ma (decrease)
IRhard Timetree	3.4 Ma (increase) 0.4 Ma (decrease)	6.3 Ma (increase) 0.3 Ma (decrease)	6.3 Ma (increase) 0.6 Ma (increase) 0.1 Ma (decrease)
IRsoft Timetree	5.7 Ma (increase 0.3 Ma (decrease)	5.8 Ma (increase) 0.2 Ma (decrease)	0.5 Ma (increase) 0.1 Ma (decrease)

## Discussion

### Primate Phylogeny

The phylogeny for 367 primates (Figs. 1, 2, 3, 4, 5) provides the most complete molecular phylogeny to date for primate genera and species. Phylogenetic relationships in Figures 1, 2, 3, 4, 5 are largely confirmatory of recent studies on higher-level relationships among primates [3], [12], [15], [21], [22], as well as relationships within Lorisiformes [3], [12], [15], , Lemuriformes [3], [7], [8], [11], [12], [15], [25]–[38], Tarsiiformes [39], [40], Catarrhini [3], [12], [15], [41]–[50], and Platyrrhini [3], [12], [15], [51]–[62]. Our supermatrix expands upon these previous studies by bringing together sequences from a wide array of molecular studies, supplemented by new data for four genes, to yield a single primate phylogeny with strong support for the monophyly of most primate genera, families, and higher-level taxa (Primates, Strepsirrhini, Lemuriformes, Lorisiformes, Haplorhini, Tarsiiformes, Simiiformes, Catarrhini, Platyrrhini). This phylogeny combines a robust framework for primate families and most genera based on nuclear genes with the expanded taxonomic coverage for genera and species that results from the inclusion of mitochondrial DNA sequences. Our primate phylogeny also provides an appropriate framework for timetree and diversification analyses of Primates.

Missing data (including gaps) comprise ∼69% of the total data set, but the distribution of missing data is decidedly nonrandom. Rather, families and most genera have one or more exemplars with high gene completeness (Fig. S2), whereas gene sampling among species with only one or a few sequences is focused on a few mitochondrial genes so that gene overlap is generally high among congeneric species and closely related genera. Wiens [63] suggested that it should be possible to design phylogenetic analyses that will resolve higher-level relationships with large numbers of slow-evolving characters and then place additional taxa on this scaffold through the use of a smaller number of more rapidly evolving characters. Along these lines, Sánchez-Gracia and Castresana [64] suggested that combined data sets may be more efficient than nuclear only or mitochondrial only data sets of the same size for species tree inference when there are both deep and shallow divergences. This design characterizes the present study, which includes a large number of slow-evolving characters (i.e., nuclear loci) for families and genera, and a smaller number of more rapidly evolving characters (i.e., mitochondrial loci) that are added onto this scaffold for an expanded assemblage of primate species. At the same time, nuclear and mitochondrial loci require heterogeneous substitution models to avoid problems with model mis-specification and it is important to analyze combined nuclear+mitochondrial supermatrices with mixed model partitioning.

The ML tree based on mitochondrial genes (Text S2) failed to recover the monophyly of several higher-level clades, including Primates and Haplorhini. These problems have previously occurred in analyses with mitochondrial DNA [15], [65], [66], but are eliminated when the mitochondrial and nuclear DNA data are combined. In addition, the separate mitochondrial and nuclear trees (Text S2) failed to recover the monophyly of *Pithecia* (mitochondrial tree) and *Lepilemur* (nuclear tree), respectively, probably because of problems with nonoverlapping sequences [67]. However, these problems were eliminated through the inclusion of both mitochondrial and nuclear DNA sequences.

All of the relationships among genera and families that were strongly supported in Perelman et al’s [12] analysis were also recovered in the present study. Differences between Perelman et al.’s [[12, fig. 1] phylogeny for primate families and genera and the phylogeny in Figure 1 pertain to clades with bootstrap support that was less than 90% in one or both studies. Perelman et al. [12] included 61 genera in their study (60 if *Mico* and *Cebuella* are treated as subgenera of *Callithrix* [6] and *Tarsius* is split into three genera (*Tarsius*, *Carlito*, *Cephalopachus*) following Groves and Shekelle [68]), whereas the present analysis includes ten additional genera (*Allocebus*, *Euoticus*, *Hoolock*, *Indri*, *Phaner*, *Procolobus*, *Prolemur*, *Rungwecebus*, *Simias*, *Tarsius* [sensu 68]), many of which were positioned with robust support relative to their sister taxa and closest outgroups: (1) *Allocebus* is the sister taxon to *Microcebus*+*Mirza* in Cheirogaleidae; (2) *Euoticus* groups with other galagids; (3) *Hoolock* groups with other hylobatids, although relationships among the four hylobatid genera are not well supported; (4) *Indri* is the sister taxon to *Avahi*+*Propithecus* in Indriidae; (5) *Phaner* groups with other cheirogaleids on the bootstrap tree, although not on the best ML tree; (6) *Procolobus* is the sister taxon to *Piliocolobus* in Colobini; (7) *Prolemur* groups with *Lemur* and *Hapalemur* in Lemuridae, although relationships among these three genera are not well resolved; (8) *Rungwebus* groups with *Papio* in Papionini; (9) *Simias* and *Nasalis* are sister taxa in Presbytini; and (10) *Tarsius* is the sister taxon to *Cephalopachus*+*Carlito* in Tarsiidae. The only genera missing from the present analysis are the atelid *Oreonax* and the lorisid *Pseudopotto*, both of which are monotypic and remain to be sampled for gene sequences. Further, *Pseudopotto martini* is a controversial taxon and is based on only two specimens of uncertain provenance [69].

Three genera (*Galago*, *Cercopithecus*, *Trachypithecus*) were recovered as paraphyletic in our analyses, but in each case these results are consistent with previous studies that have included broad taxon sampling within these taxa. Chatterjee et al. [15] and Fabre et al. [3] recovered *Otolemur* and *Euoticus* inside of *Galago* based on their supermatrix studies. Perelman et al. [12] also recovered *Otolemur* inside of *Galago*, but *Euoticus* was not included in their study. Our analyses placed *Otolemur* inside of *Galago*, and *Euoticus* as the sister taxon to *Galago*+*Otolemur*. However, bootstrap support for the monophyly of *Galago*+*Otolemur* to the exclusion of *Euoticus* was<50%. Numerous studies have recovered *Cercopithecus* as paraphyletic [3], [12], [43], [70]. Our results agree with these studies by placing *Erythrocebus* and *Chlorocebus*, both of which are terrestrial, in a clade with terrestrial *Cercopithecus* species. This collective group is the sister taxon to an arboreal *Cercopithecus* clade [3]. Retroposon insertions [70] and Perelman et al.’s [12] supermatrix further suggest that *Miopithecus* is nested inside of *Cercopithecus*, specifically as the sister taxon to arboreal *Cercopithecus*, whereas Fabre et al. [3] recovered *Miopithecus* as the sister taxon to *Cercopithecus*+*Chlorocebus*+*Erythrocebus*. Our results suggest that *Miopithecus* and *Allenopithecus* are sister taxa and together comprise the sister taxon to *Cercopithecus*+*Chlorocebus*+*Erythrocebus*. Finally, *Trachypithecus* paraphyly, with one or more species of *Semnopithecus* nested inside of this genus, has been recovered with mitochondrial data, Y chromosomal data, retroposon insertions, and nuclear supermatrices [12], [45]. Taxonomic revisions are required to parcel these paraphyletic genera, as well as their embedded subtaxa (i.e., *Otolemur* in *Galago*, *Erythrocebus* and *Chlorocebus* in *Cercopithecus*, *Semnopithecus* in *Trachypithecus*) into monophyletic units. In some cases these revisions are underway. For example, Karanth [71] has suggested a classification wherein langurs of the Indian subcontinent are placed in *Semnopithecus*, whereas leaf monkeys of Southeast Asia are placed in *Trachypithecus*. However, the placement of northern species remains less clear [71].

A potential shortcoming of supermatrices that include both mitochondrial and nuclear data is that combined analyses mask genuine cytonuclear dissociation (i.e., mito-nuclear discordance), which occurs when nuclear and mitochondrial genes have different evolutionary histories owing to processes such as introgression of mtDNA, demographic disparities, and sex-biased asymmetries including male-biased dispersal [72]–[75]. It will be important in future studies to improve both gene and taxon sampling for primate species, to construct separate trees based on mitochondrial and nuclear loci, and to tease apart differences that arise from incomplete lineage sorting versus other types of discordance including introgression and sex-biased dispersal [75]–[77]. Among primates, mito-nuclear discordance has been suggested for Asian colobines belonging to *Presbytis*, *Trachypithecus*, and *Semnopithecus* [46], [77], [78], and the Malagasy mouse lemurs *Microcebus griseorufus* and *M. murinus* [73].

### Primate Divergence Times

Relaxed clock methods have previously been used to estimate divergence times among primate lineages, but these studies were based on smaller data sets (taxa and genes) and employed fewer calibrations than the present study. Further, we performed analyses with two different models for evolutionary rates (autocorrelated, independent). There were only six instances of timetree dates that were younger than minimum constraints or older than maximum constraints in soft-bounded analyses. The mean violation was 3.6 Ma, which suggests that there are only minor inconsistencies among the calibrations that were employed in this study.

We estimate that crown primates last shared a common ancestor ∼71–63 million years ago. This date is slightly older than Chatterjee et al.’s [15] relaxed clock date of 63.7 Ma for Primates, but markedly younger than most timetree dates (Table 4), some of which are as old at 85–90 Ma [12], [25]. Latest Cretaceous dates for Strepsirrhini and Haplorhini are also common among relaxed clock studies, but our analyses suggest that strepsirrhines last shared a common ancestor 55.3−53.3 Ma near the Paleocene-Eocene boundary, and that haplorhines last shared a common ancestor 64.2−58.4 Ma in the Paleocene. Both of these dates are in good agreement with Meredith et al.’s [22] timetree estimates for these clades. With the exception of the basal split among extant Primates, which is latest Cretaceous in age in some analyses, our timetree estimates are concordant with the hypothesis that the KPg mass extinction opened up ecospace that promoted intraordinal diversification [22]. Our timetree estimates also reduce the disagreement between paleontological and molecular estimates for the most recent common ancestor of Primates, and agree with Steiper and Seiffert’s [79] estimated divergence times (70−63 Ma). Steiper and Seiffert [79] showed that molecular evolutionary rates in primates are inversely correlated with three life history variables (body size, absolute endocranial volume, relative endocranial volume), and that the last common ancestor of living primates had a very small body size, absolute endocranial volume, and relative endocranial volume. They subsequently reconstructed a timescale for primates by predicting molecular rates from the reconstructed phenotypic values for a large phylogeny of living and extinct primates. Importantly, Steiper and Seiffert’s [79] analysis was an attempt at correcting for the effects of convergent rate slowdowns in primates [80]–[83]. Our results suggest that relaxed clock methods can also address convergent rate slowdowns, as is the case for primates [79]–[83], provided that there are sufficient calibrations throughout the tree.

**Table 4:** A comparison of mean divergence dates for select nodes in the present study and previous studies. Dates are in millions of years.

Clade	This study	Meredithet al. [22]	Perelmanet al. [12]	Fabreet al. [3]	Chatterjeeet al. [15]	Steiper andYoung [83]
Primates	67.8	71.5	87.2	82.2	63.7	77.5
Strepsirrhini	54.2	55.1	68.7	71.9	51.6	57.1
Lemuriformes+Chiromyiformes	50.0	51.6	58.6	63.3	46.2	NI
Lemuriformes	31.8	29.6	38.6	44.8	32.4	40.9
Lorisiformes	34.7	35.4	40.3	40.9	37.5	NI
Haplorhini	61.2	62.4	81.3	74.5	NI	NI
Simiiformes	40.6	30.1	43.5	39.8	42.8	42.9
Catarrhini	25.1	20.6	31.6	25.0	29.3	30.5
Hominoidea	17.4	14.4	20.3	19.5	21.5	NI
Hominidae	15.1	NA	16.5	15.4	15.9	18.3
Homininae	8.0	NA	8.3	10.1	10.7	8.6
*Homo*+*Pan*	6.7	NA	6.6	7.5	8.1	6.6
Cercopithecoidea	13.2	NA	17.6	14.5	23.4	NI
Colobinae	8.9	NA	12.3	9.6	18.4	NI
Cercopithecinae	8.4	NA	11.5	10.5	NA	9.9
Platyrrhini	23.3	14.6	24.8	15.9	26.6	20.8

NI, divergence date not included in study.

We recovered divergence dates within Strepsirrhini, including Lorisiformes, Madagascan Strepsirrhini, and Lemuriformes that are similar to dates from Meredith et al. [22], but younger than dates from other studies (Table 4). Divergence dates for Simiiformes and its subclades, in turn, are generally in good agreement with other relaxed clock studies including Chatterjee et al. [15], Fabre et al. [3], and Perelman et al. [12] (Table 4). For example, our mean date for Simiiformes based on four different analyses is ∼41 Ma whereas Chatterjee et al. [15], Fabre et al. [3], and Perelman et al. [12] recovered dates of ∼43, ∼38, and ∼43 Ma, respectively. By contrast, Meredith et al. [22] recovered younger dates for Simiiformes, Catarrhini, Hominoidea, and Platyrrhini (Table 4). Meredith et al.’s [22] younger dates for these clades may reflect the difficulties of timetree analyses for clades such as Mammalia that include taxa with a wide range of life history characters (e.g., baleen whales versus muroid rodents) and a correspondingly wide range of molecular evolutionary rates.

The mean timetree date for Platyrrhini based on our analyses was 23.3 million years, which is slightly older than Hodgson et al.’s [59] date of 19.5 Ma that was obtained with complete mitochondrial genome sequences and Thorne and Kishino’s [84] multidivtime program. Hodgson et al. [59] concluded that the fossil platyrrhines *Dolichocebus*, *Tremacebus*, and *Chilecebus*, which date to ∼20 Ma, must be stem platyrrhines because they are older than Hodgson et al.’s [59] estimate for the most recent common ancestor of extant platyrrhines at 19.5 Ma. Poux et al. [85] and Fabre et al. [3] also recovered dates for Platyrrhini that are too young for the inclusion of the aforementioned fossil in crown Platyrrhini. However, our timetree dates for Platyrrhini leave open the possibility that these fossil taxa are members of the crown clade.

Our results also demonstrate that timetree analyses are sensitive to different clock models (i.e., autocorrelated versus independent rates), as Meredith et al. [22] previously reported in their analyses of divergence times among mammalian families. In comparison to analyses with an autocorrelated rates model, timetree dates based on an independent rates model yielded older dates for the base of Primates, similar dates for the suborders Strepsirrhini and Haplorhini, and younger dates for almost all other primate nodes (Table S1).

Finally, we note that the potential complication of modeling older divergences with nuclear data and more recent divergences with mitochondrial data, as in the present study, can be addressed in future studies with increased nuclear sampling at the species level.

### Ancestral-Area Reconstructions

Beard [16] suggested an Asian origin for crown primates based on an analysis that coded the total geographic area of five extant (Strepsirrhini, Tarsiidae, Simiiformes, Cynocephalidae, Scandentia) and eight extinct (Paromomyidae, Micromomyidae, Carpolestidae, Plesiadapidae, *Saxonella*, *Altanius*, Omomyidae, *Altiatlasius*) euarchontan lineages in three different areas (Africa, Asia, North America). As discussed by Silcox [2], Beard’s [16] topology was not based on an explicit matrix, but instead was cobbled together from different sources. Silcox [2] expanded Beard’s [16] taxonomic sampling and used a matrix-based topology as the framework for her ancestral area reconstructions. She concluded that an origin for crown primates in Africa, Asia, Europe, or North America was possible. Bloch et al. [1] performed analyses using the total geographic distributions of the taxa in questions, as in Beard [16] and Silcox [2], as well as the location of a taxon’s first fossil occurrence. Bloch et al. [1] recovered Asia or North America as the ancestral area for crown primates based on the former approach, and North America as the ancestral area based on the latter approach.

These authors all used Fitch parsimony to infer ancestral areas. Fitch parsimony was coopted from phylogenetics rather than developed explicitly for historical biogeographic reconstruction. A disadvantage of monomorphic ancestral area reconstruction methods, including Fitch parsimony, is that these methods disallow geographic ranges at ancestral nodes that include more than one area [18]. By contrast, an advantage of both DEC and MAC parsimony is that these methods explicitly allow ancestors to have geographic ranges that encompass two or more areas. The application of DEC and MAC parsimony with our molecular phylogeny suggests that Asia played a prominent role in the early evolutionary history of crown primates. Asia was reconstructed as the ancestral area of Primates, Haplorhini, Catarrhini, and Hominoidea, and was among multiple areas or one of the alternate reconstructions for Strepsirrhini, Simiiformes, and Cercopithecoidea. As discussed below, these results must be tempered against the backdrop of the fossil record given that ancestral area reconstructions are sensitive to taxon sampling and the inclusion of fossils [18], whereas our molecular phylogeny is *sans* fossils.

Previous studies [1], [2], [16] that included Asia as one of the possible ancestral areas for crown Primates are generally consistent with our results. DEC and MAC parsimony reconstructions that place the most recent common ancestor of haplorhines in Asia are also consistent with prevailing views based on the fossil record [86].

By contrast, ancestral reconstructions that place the most recent common ancestors of Simiiformes, Catarrhini, Hominoidea, Hominidae, and Cercopithecoidea in Asia are in varying degrees of conflict with predominant views based on the fossil record. The oldest simiiform fossils are eosimiids from Asia, and it is generally held that stem simiiforms originated in Asia before migrating to Africa in the Middle or Late Eocene [87], [88]. Stem catarrhines, in turn, are exclusively Afro-Arabic with the possible exception of Asian amphipithecids [86], [88]. In the case of both Simiiformes and Catarrhini, DEC and MAC parsimony results are potentially impacted by the absence of key fossil taxa in our molecular phylogeny including Oligopithecidae, Parapithecoidea, Proteopithecidae, Propliopithecidae, and Saadaniidae, all of which are stem simiiforms and/or stem catarrhines [89], [90].

Most authors have also concluded that Africa is the ancestral area for crown Hominoidea, and that Eurasian hylobatids and ponginines represent independent dispersals from Africa [91], [92]. However, Stewart and Disotell [93] and Begun [94] suggested that crown hominids originated in Asia and that the ancestor of African apes and humans (Homininae) re-entered Africa in the late Miocene. Folinsbee and Brooks’ [95] PACT analysis of area cladograms for hominoids, proboscideans, and hyaenids provides additional support for the hypothesis that crown hominoids last shared a common ancestor in Asia, and that the ancestor of crown Hominidae re-entered Africa. Finally, our results suggest that Africa and Asia are equally likely ancestral areas for Cercopithecoidea, whereas the fossil record suggests that Old World monkeys have a most recent common ancestor in Africa [96].

In two instances the results of DEC analyses suggest that dispersal events occurred on older branches than implied by any of the MAC parsimony reconstructions. First, DEC results suggest that the most recent common ancestor of Simiiformes occupied a geographic range that included Asia and the New World, and that the ancestral range of this taxon was sundered in conjunction with the cladogenic separation of catarrhines and platyrrhines. These results imply that Primates were present in South America prior to the most recent common ancestor of Simiiformes, ∼44−37 Ma, whereas the oldest primate fossils from South America are from the early Miocene and are either stem [59] or crown platyrrhines [97]. Putative stem Simiiformes, in turn, are known from Asia and Africa [98]–[104]. We favor the MAC parsimony results, which are more compatible with the fossil record and support dispersal to the New World after platyrrhines diverged from catarrhines. However, MAC parsimony results suggest that dispersal occurred from Asia to South America rather than from Africa to South America as is more commonly assumed [85]. An ancestral area of Asia for crown Simiiformes may be an artifact of not including stem simiiforms in our analyses. Second, DEC results suggest an earlier dispersal of macaques to Asia than MAC parsimony. DEC results suggest that dispersal to Asia occurred in the common ancestor of crown *Macaca*, and that this ancestor occupied both Africa and Asia. MAC parsimony results, in turn, suggest that the ancestor of Asian macaques dispersed from Africa to Asia after *Macaca sylvanus* separated from the latter clade. The fossil record of *Macaca sylvanus* includes northern Africa (e.g., Tunisia) and much of Europe (e.g., Italy, France, Germany, Austria) [96] and supports the DEC hypothesis that the common ancestor of living macaques dispersed from Africa to Asia (or at least Eurasia) before *M. sylvanus* diverged from other *Macaca* species.

Extinct taxa were not included in our molecular phylogeny, but are critical for understanding the historical biogeography of crown primates. MAC parsimony analyses with phylogenetic trees from Seiffert et al. [20] that included fossil and living taxa provide additional support for the hypothesis that crown primates last shared a common ancestor that resided in Eurasia in analyses that allowed for five areas, but were equivocal in analyses with six areas. Unfortunately, existing morphological data sets with extensive taxon sampling for key fossil groups are difficult to combine with molecular data sets because extant taxa are not well sampled in the former. Future analyses of primate historical biogeography will benefit from combined sets that include a broad assemblage of fossils in the morphological partition, and broad, overlapping taxon sampling between the molecular and morphological partitions for extant taxa.

### Diversification Analyses

Analyses with Groves 93, Groves05, and Groves 05+all provide support for a diversification rate increase in the late Miocene/Pliocene (mean = 7 Ma). This rate shift corresponds to the onset of an increase in fossil primate diversity at approximately the same time (Fig. 8). The late Miocene was characterized by elevated global mean temperatures [105], [106], which may have promoted primate diversification. The increase in fossil diversity beginning at ∼7 Ma is attributable, in part, to an increase in the diversity of Old World monkeys, which became more widespread in Africa and occur for the first time in Eurasia in the late Miocene [107].

All of our diversification analyses provide support for a Pleistocene rate slowdown, although in two cases this rate slowdown was preceded by a slightly earlier Pleistocene rate increase. Pleistocene rate slowdowns have been documented in other diversification studies and may result from our inability to recognize recently separated lineages [108]. Viewed from the perspective of species concepts, rate decreases in the recent past may be expected when lineages are defined by the biological species concept (BSC) because incipient lineages on the road to eventual reproductive isolation have had insufficient time to acquire the hallmarks of “good” biological species. The proclivity of the BSC to discount incipient lineages suggests that de Queiroz’ [109], [110] general lineage concept of species, wherein alternate species concepts represent different stages in the speciation process, provides a more appropriate framework for defining lineages in diversification analyses. Along these lines the “diagnosable” version of the phylogenetic species concept (PSC), in which a species is defined as “the smallest diagnosable cluster of individuals within which there is a pattern of ancestry and descent” [111], represents the earliest recognizable stage in the emergence of a new lineage. Even the diversification analyses with Groves05+taxonomy, which has been strongly influenced by widespread adoption of the PSC by primate taxonomists [5], [6], [9], [11], detected rate decreases in the Pleistocene. However, the average age of the Pleistocene rate decrease becomes progressively younger from Groves93 (1.1 Ma) to Groves05+(0.3 Ma), which suggests that the PSC is effective at discriminating among all but the most recent incipient lineages. The discovery and proper vetting of newly proposed primate species, especially in groups with rapidly increasing taxonomic diversity such as Malagasy lemurs, is critical for obtaining accurate estimates of diversification rates in the recent past.

Two of four diversification analyses with Groves05+provide support for a rate increase in the Pleistocene that precedes the subsequent rate decrease. The possibility of a rate increase in the Pleistocene is consistent with the Pleistocene Refugia hypothesis. Living primates have distributions that are centered in four geographic regions: Africa, Madagascar, Asia, and South America. Each of these regions includes tropical/subtropical habitats that may have contracted and expanded in synchrony with Pleistocene glaciation cycles [112]–[117]. Pleistocene glaciations in Asia may have promoted speciation among primates through the lowering of sea level, which facilitated travel among Asian islands, rather than through isolation as in Africa and South America [118]. Another possible cause for the Pleistocene rate increase is the “pull of the Recent”, which is a consequence of an excess of newly emergent lineages that have insufficient time to go extinct [119]. Stadler’s [120] method explicitly accounts for the pull of the Recent, but may still detect a rate increase if there is artificial oversplitting. Clearly, diversification analyses of lineages through time are fundamentally linked to our concept of “lineage”, and conclusions pertaining to relatively recent rate increases or decreases must confront this issue.

**Figure 9:**
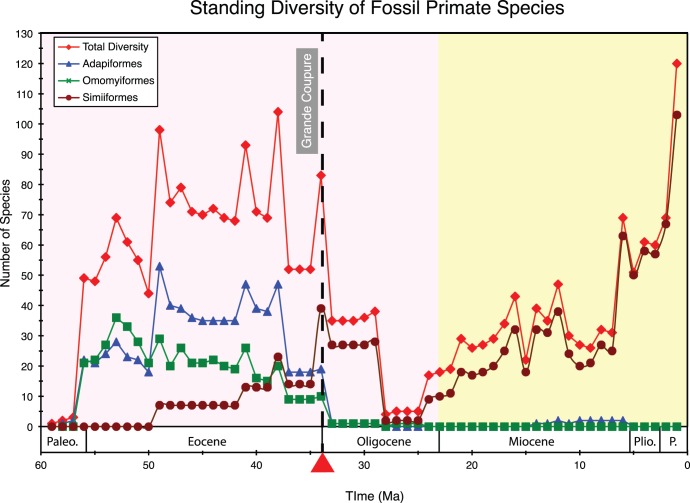
Standing diversity of fossil primate species throughout the Cenozoic. Total primate diversity includes all crown primate fossils found within the Paleobiology Database and Hartwig [149]. Adapiformes and Omomyiformes are extinct lineages. The position of the Grande Coupure at ∼ 33.9 Ma is indicated by a dashed black line. Pink background = Paleogene; yellow background = Neogene.

None of our diversification analyses detected pre-Miocene rate increases or decreases, even when the timetrees were truncated at the beginning of the Pleistocene (2.6 Ma) before running TreePar analyses to address the possibility that Pleistocene rate changes are obscuring pre-Pleistocene dynamics. This result contrasts with the fossil record, which suggests that primate diversity was relatively high in the early Eocene, plummeted in the Oligocene, and gradually rebounded thereafter (Fig. 9). The high diversity of primates in the Eocene is associated with warm temperatures and an expansion of tropical and subtropical habitats during this epoch. The decrease in primate diversity at the Eocene-Oligocene boundary corresponds to the “Grande Coupure” in Europe and its temporal equivalents in Africa, Asia (“Mongolian Remodeling”), and North America [121] (Fig. 9). The Grande Coupure represents a major terrestrial faunal turnover and coincides with the onset of Oligocene glaciation and cooling [122], [123]. The Oligocene decrease in diversity was largely driven by the demise of two clades, Adapiformes and Omomyiformes, which were the most diverse crown primate groups during much of the Cenozoic [89], [124]. The post-Oligocene rebound in fossil diversity, in turn, owes mainly to an increase in the number of simiiform species. TreePar analyses with four Groves05+timetrees that compared one-rate models to two-rate models, with the additional requirement that the rate shift was forced to occur within 0.5 Ma of the Eocene-Oligocene boundary (i.e, 33.9+/−0.5 Ma), resulted in log likelihood differences (0.90 to 4.26) that were insignificant after adjusting the Type 1 error rate to α = 0.05 (Methods). In addition, we compared diversification rates based on the fossil record with diversification rates based on molecular timetrees for 11 consecutive five million year intervals from 55 to 0 Ma, and in every case *r* values were insignificant (Table 5).

**Table 5:** Diversification rates ([speciation – extinction]/lineage/million years) for 11 five million years intervals based on molecular timetrees and the fossil record.

Time Interval (Ma)	Fossil Rate	AUTOhard Timetree Rate	AUTOsoft Timetree Rate	IRhard TimetreeRate	IRsoft TimetreeRate
5 to 0	1.39E-01	2.11E-02	2.98E-01	−3.63E-04	−2.95E-01
10 to 5	2.86E-02	2.68E-01	5.91E-02	2.71E-01	2.98E-01
15 to 10	4.84E-02	1.64E-02	6.60E-02	4.41E-04	−2.23E-01
20 to 15	1.65E-01	8.00E-02	8.01E-05	6.94E-02	2.10E-01
25 to 20	1.25E-01	4.19E-02	1.03E-02	5.66E-02	−2.95E-04
30 to 25	−1.14E-01	2.03E-02	8.68E-04	1.60E-02	−1.11E-01
35 to 30	−5.34E-02	5.97E-02	3.35E-03	2.08E-01	1.29E-01
40 to 35	−1.96E-03	1.24E-02	7.52E-03	1.89E-01	2.23E-06
45 to 40	−1.28E-02	9.16E-02	8.03E-04	1.70E-01	6.34E-02
50 to 45	1.36E-02	1.01E-06	6.64E-03	1.76E-01	1.07E-01
55 to 50	2.25E-01	1.64E-01	1.43E-03	2.15E-01	4.54E-02
Correlation coefficient, *r*		0.249	0.272	−0.087	−0.019

Clearly there is a discrepancy between the fossil record, which provides evidence of a major turnover event in primate diversity beginning with the Grande Coupure, and diversification analyses of living taxa that failed to uncover evidence for this turnover. McInnes et al. [125] have also discussed the inadequacies of molecular phylogenies for detecting ancient diversification shifts. By contrast, Morlon et al. [126] have shown that diversification dynamics inferred from molecular phylogenies can be concordant with the fossil record if rate variation through time and among major taxonomic groups is taken into account. However, the application of Morlon et al.’s [126] approach is arbitrary and presently lacks a systematic framework for implementation. Also, unlike Cetacea where taxonomic groups inferred by Morlon et al. [126] to have declining diversity still have extant representatives, Adapiformes and Omomyiformes are extinct clades that are entirely missing from molecular chronograms for extant Primates. Rabosky [127] has previously shown that phylogenetically clustered extinction events (i.e., entire clades) erase the signature of extinction from molecular phylogenies.

Apparent discrepancies between fossil diversity and the signature of molecular phylogenies may also be attributed to sampling bias in the fossil record given that rates of fossil preservation have varied through time and in different geographic regions [128]–[132]. The fossil record of primates is a case in point and it has been estimated that only ∼7% of Cenozoic primate species have so far been discovered [133], [134]. Sampling biases in the primate fossil record include better sampling in North America and Europe than in Africa and Asia [132], and notoriously poor fossil preservation during the middle of the Oligocene, especially in Africa [132], [135]. The latter sampling bias may account for the decrease in diversity across the early Oligocene-middle Oligocene transition (∼29−28 Ma, Fig. 9), whereas the earlier plummet in diversity at ∼34 (i.e., Grande Coupure, Fig. 9) represents a major terrestrial faunal turnover associated with real climatic changes at the Eocene**–**Oligocene transition [133]. A challenge for future studies is to assess whether or not molecular phylogenies retain any signature of major turnover events such as the Grande Coupure that impacted Primates and other mammalian taxa, while at the same time accounting for sampling bias in the fossil record.

## Materials and Methods

### Gene and Taxon Sampling

We assembled a data set comprising segments of 69 nuclear genes and ten mitochondrial genes. Sequences for 54 nuclear genes were taken from Perelman et al.’s [12] nexus file with modifications to eliminate problems with probable contaminants and misidentified sequences (Table S2); sequences for 15 additional nuclear genes (*ABO*, *CXCR4*, *CXCR5*, Epsilon globin, *FGA*, *IRBP* intron 1, *IRBP* intron 3, *MC1R*, *NRAMP*, *PRNP*, *VWF* intron 11) were obtained from GenBank; and new sequences (JX856181-JX856283, JX869897-JX869930) for exons of four nuclear genes (*GHR*, *IRBP*, *VWF, TTN*) were combined with previously published GenBank sequences for these loci. Mitochondrial sequences included eight protein-coding genes (*COB*, *COI*, *COII*, *COIII*, *ND2*, *ND3*, *ND4*, *ND4L*) and two RNA genes (*12S rRNA*, *16S rRNA*). Accession numbers for previously published sequences are provided in Table S3.

Groves recognized 233, 354, and 376 primate species in his 1993, 2001, and 2005 classifications, respectively [4]–[6]. More recent studies suggest that the number of extant primate species is a least 450 (Text S1). We searched GenBank for mitochondrial and nuclear gene sequences belonging to these taxa, which resulted in a final data set that included at least one gene for 367 of 450 primate species. We excluded mitochondrial protein-coding sequences with numt (nuclear mitochondrial DNA) signatures including frameshift mutations and stop codons. A few taxa were represented by a single sequence in GenBank, but were omitted from our data set because of potential problems with non-overlapping sequences when there was no sequence overlap with congeneric species. Outgroup taxa were the same as Perelman et al. [12] and included two dermopterans (*Cynocephalus volans*, *Galeopterus variegatus*), two scandentians (*Tupaia glis*, *T. minor*), and a composite lagomorph, all of which are from the same superordinal group (Euarchontoglires) that also includes Primates [22]. Of these, dermopterans are the sister taxon to primates [22].

Given that there have been significant changes to primate taxonomy during the history of GenBank, we updated older GenBank entries to the newer Groves 05+taxonomy. This frequently involved elevating taxa with subspecific status on GenBank to full species status. There are also GenBank entries that lack new species names, which can only be found in the original papers, e.g., numerous *Lepilemur* species of Louis et al. [36]. In some cases older GenBank sequences for primates were avoided because they could not be satisfactorily updated to the new Groves05+taxonomy.

### PCR and DNA Sequencing

Methods and primers described by Meredith et al. [22] were used to amplify segments of *IRBP*, *GHR*, *TTN*, and *VWF* for 32 (*TTN*), 34 (*IRBP, VWF*), and 35 (*GHR*) primate species.

### Alignments and Data Concatenation

We employed unaltered alignments or alignments with minor adjustments for 53/54 nuclear genes that were included in Perelman et al.’s [12] nexus file. *ADORA3* sequences were aligned from scratch given numerous problems with Perelman et al.’s [12] alignment (Table S2) and the availability of additional *ADORA3* sequences for Lemuriformes. Alignments for all of the other nuclear genes, as well as mitochondrial genes, were performed with Se-Al [136]. Ambiguous regions of the *12S* and *16S rRNA* genes were excluded prior to phylogenetic analysis. Alignments for 79 different partitions were combined with SequenceMatrix 1.7.8 [137]. The resulting alignment comprised 61199 bp and has been deposited in TreeBase (ID 13451).

### Phylogenetic Analyses

Maximum likelihood analyses were performed with RAxML 7.2.8 on Cipres [138], [139]. Each of seventy-nine different gene segments was allowed to have its own parameter estimates under the GTR+Γ model of sequence evolution. Rapid bootstrapping (500 replications) and a search for the best ML tree were performed in a single program run. Bootstrap replications were performed with a GTRCAT approximation to the GAMMA distribution and 25 distinct rate categories. The search for the best tree was conducted under GTRGAMMA rather than GTRCAT. Default parameters were employed for all other settings.

### Timetree Analyses

The mcmctree program in PAML 4.4b [140] was used to perform molecular dating analyses. Mcmctree implements the MCMC algorithms of Rannala and Yang [141]. Gene segments were binned into eight categories: mitochondrial protein-coding genes, mitochondrial rRNA, X-linked nuclear genes, Y-linked nuclear genes, and four groups of autosomal genes based on rates of evolution (Table S4). Analyses with usedata = 3, which is used to calculate Hessian matrices, allowed each of the eight partitions to have its own GTR+Γ model. Analyses with usedata = 2 were performed with autocorrelated rates and hard-bounded constraints (AUTOhard), autocorrelated rates and soft-bounded constraints (AUTOsoft), independent rates and hard-bounded constraints (IRhard), and independent rates and soft-bounded constraints (IRsoft). We set 1 time unit = 100 million years following Rannala and Yang [141]. Analyses were run with cleandata = 0 in PAML. Shape (α) and scale (β) parameters for rgene_gamma, which describes the gamma prior for the overall rate parameter μ, were calculated as in Meredith et al. [22]. Calculations for the shape and scale parameters for sigma_gamma, which describes the gamma prior for the rate-drift parameter (σ^2^), assumed an age of 85.9 million years for the most recent common ancestor of Euarchontoglires [22]. Analyses were run for 100,000 generations after a burn-in of 10,000 generations. Chains were sampled every 20 generations. Each analysis was run twice to check for convergence.

Minimum and maximum fossil calibrations were applied to 14 nodes (Text S3), seven of which were taken from Meredith et al. [22]. Minimum ages were based on the age of the oldest unequivocal fossils belonging to each clade. Maximum ages were based on the maximum of stratigraphic bounding, phylogenetic bracketing, and phylogenetic uncertainty [18], [22], [142]. Stratigraphic bounding encompassed two successive chronologic units that did not contain any fossils from the clade of interest. Dates used in stratigraphic bounding are from Gradstein and Ogg [143]. We recognized the following chronologic units in succession from youngest to oldest: Pleistocene, Pliocene, late Miocene (Tortonian+Messian stages), middle Miocene (Langhian+Serravallian stages), early Miocene (Aquitanian+Burdigalian stages), late Oligocene (Chattian stage), early Oligocene (Rupelian stage), late Eocene (Priabonian stage), late middle Eocene (Bartonian stage), early middle Eocene (Lutetian stage), early Eocene (Ypresian stage), late Paleocene (Thanetian stage), middle Paleocene (Selandian stage), and early Paleocene (Danian stage). Phylogenetic bracketing encompassed the age of the oldest stem fossils that were up to two nodes below the divergence event. Phylogenetic bracketing allowed for the possibility that taxa of uncertain phylogenetic affinities belong to the crown clade, first outgroup, or second outgroup.

### Ancestral-Area Reconstructions

Ancestral areas were reconstructed with dispersal-extinction-cladogenesis (DEC) [17], [19] and minimum area change (MAC) parsimony [18]. The DEC analysis used the timetree based on autocorrelated rates and soft-bounded constraints. DEC is a likelihood-based approach that takes account of both the topology and branch lengths, whereas MAC parsimony ignores branch lengths and only takes into account the underlying topology. We recognized four geographic areas (Africa, Madagascar, Asia [including the Arabian Peninsula], New World) for extant primates and scored the presence or absence of living primate species in each of these four areas. Geographic area information for most species was extracted from Groves [6]. Species distributions for recently described species were culled from additional sources [9], [11], [68], [144]–[148]. Ancestral area reconstructions were performed with a maximum of two areas per internal node given that all living primates excepting *Homo sapiens* have distributions that are restricted to one or at most two areas. *H. sapiens* was coded as present in Africa and Asia given that our own species occupied these areas before migrating to Madagascar or the New World. We also performed MAC parsimony ancestral area reconstructions for taxa that were included in Seiffert et al.’s [20] phylogenetic analysis of fossil and living primates. These analyses were performed with eight phylogenetic trees from Seiffert et al. [20, fig. 2], all of which were rooted with *Tupaia*, and either five (North America, Africa, Eurasia, Madagascar, South America) or six (North America, Africa, Europe, Madagascar, Asia, South America) geographic areas with a maximum of two areas per internal node. Area coding for fossil taxa was based on Hartwig [149] and the Paleobiology Database (http://paleodb.org).

### Diversification Analyses

TreePar [120] was used to detect temporal shifts in the diversification rate under a birthdeath model. Analyses were executed with four different timetrees (autocorrelated rates with hard-bounded constraints, autocorrelated rates with soft-bounded constraints, independent rates with hard-bounded constraints, independent rates with soft-bounded constraints). We performed analyses with the full complement of primate species (i.e., Groves05+taxonomy) and with pruned trees that eliminated species that were not included in Grove05 and Groves93. Taxon completeness for these analyses was 81.6% (367/450 species) for Groves05+, 81.1% for Groves05 (305/376 species), and 90.6% (211/233 species) for Groves93. Likelihood ratio tests were employed to detect statistically significant rate shifts at P≤0.05. The results of simulations under a constant rate birthdeath model were employed to assess and correct for the real Type 1 error rate, i.e., how often the constant model was rejected using a likelihood ratio test.

### Fossil Diversity

The standing diversity of fossil primate species throughout the Cenozoic was estimated with a database for crown primates that combined temporal ranges for species from the Paleobiology Database (http://paleodb.org; data downloaded on 16 October 2011) with additional taxon ranges from Hartwig [149] for species that were missing from the Paleobiology Database. The combined database was pruned to exclude taxa that were not identified to the species level except in cases where all occurrences of a genus were species indeterminate, in which case the genus was considered to represent a single species. Species ranges were taken to include the complete time interval spanned by first and last occurrence data.

## Supporting Information

Figure S1 Node labels for Table S1.
(PDF)

Figure S2 **Graphical representation of gene and taxon completeness.** Black ×  = gene present in data set; red ×  = gene present in data set, but sequence length<50% of the length of the longest sequence.
(PDF)

Table S1 Mean, median, and 95% credibility intervals for four different methods. Node numbers are given in Figure S1.
(XLS)

Table S2 Compendium of modifications to Perelman et al.’s [12] data set.
(DOCX)

Table S3 List of GenBank accession numbers.
(DOC)

Table S4 List of genes that were included in eight different mcmctree partitions.
(DOCX)

Text S1 Compendium of primate species that have been recognized subsequent to Groves [6].
(DOCX)

Text S2 **Timetrees and RAxML phylograms in Newick format.** 1. Newick timetree with autocorrelated rates and hard-bounded constraints. 2. Newick timetree with autocorrelated rates and soft-bounded constraints. 3. Newick timetree with independent rates and hard-bounded constraints. 4. Newick timetree with independent rates and soft-bounded constraints. 5. RAxML phylogram based on the 61199 bp concatenation of 69 nuclear and ten mitochondrial loci. 6. RAxML phylogram based on ten mitochondrial genes. 7. RAxML phylogram based on 69 nuclear genes.
(DOCX)

Text S3 **Fourteen minimum and 14 maximum calibrations that were employed in primate timetree analyses with mcmctree** [**140**], [**141**] **.** Minimum calibrations were based on the oldest crown fossil belong to each clade. Maximum calibrations were based on stratigraphic bounding (two chronologic units), phylogenetic bracketing (two outgroups), and phylogenetic uncertainty [18], [22].
(DOCX)
