# Supplementary material for: Macroevolutionary Dynamics and Historical Biogeography of Primate Diversification Inferred from a Species Supermatrix
Source: PLoS One. 2012 Nov 16;7(11):e49521. doi: 10.1371/journal.pone.0049521 (PMC3500307; doi:10.1371/journal.pone.0049521)
Supplement: Table S2 — Compendium of modifications to Perelman et al.’s [12] data set. (DOCX) [file pone.0049521.s004.docx]

Table S2. We made several corrections and modifications to Perelman et al.’s (2011) data set, all of which are outlined below. All of these modifications were incorporated into our supermatrix.

Segment 1 (ABCA1): *Nycticebus pygmaeus* (HM765363) is 99% identical to *Homo sapiens* sequences on GenBank, but only 73-74% identical to other *Nycticebus* spp. This sequence was therefore deleted.

Segment 2 (ADORA3): Several sequences in the nexus matrix do not match GenBank entries that should have been in the matrix. *Ateles belzebuth* from Perelman et al.’s (2011) nexus matrix is 100% identical to *Canis lupus familiaris* and does not match Perelman et al.’s (2011) GenBank sequence for *Ateles belzebuth* (HM765181). *Ateles hybridus* from Perelman et al.’s (2011) nexus matrix is 100% identical to *Sus scrofa* (e.g., AK239615). There is no GenBank sequence for *Ateles hybridus* ADORA3. *Ateles geoffroyi* from the nexus matrix is most similar to *Tamias striatus* (AY011207; 92% similar for 321 region of overlap) and does not match Perelman et al.’s *Ateles geoffroyi* sequence in GenBank (HM765184; 80% similarity). *Alouatta belzebul* is most similar to xenarthran sequences on GenBank (e.g., *Euphractus sexcinctus*, AY011193, 91% similar for 321 bp overlap) and does not match Perelman et al.’s *Alouatta belzebul* sequence (HM765171, 83% similarity). *Arctocebus calabarensis* ADORA3 from Perelman et al.’s nexus matrix is 100% identical to *Loxodonta africana* (AY011204, 321 bp overlap) and does not match Perelman et al.’s GenBank sequence (HM765244) for *Arctocebus*. Lagomorpha ADORA3 sequence in Perelman et al.’s matrix is 100% identical to *Rattus norvegicus* (NM_012896). Given these problems, and also because additional Lemuriformes sequences are available on Genbank (e.g., *Microcebus* spp.), we constructed an entirely new ADORA3 alignment based on GenBank sequences.

Segment 5 (APP): We realigned portions of *Ateles belzebul*.

Segment 6 (AXIN1): Segment should end at position 3673 instead of 3674. Otherwise there are three sequences (*Eulemur rufus, Eulemur mongoz, Callicebus caligatus*) that are entirely missing except for the last base (which is “T” in all three).

Segment 7 (BCOR): Sequence boundary should start on 3674 instead of 3675. *Hapalemur occidentalis* (HM764049) is only 77-78% identical to other primate sequences (77% identical to *Hapalemur griseus*). Given this high level of mismatch we deleted *H. occidentalis* from the BCOR dataset.

Segment 9 (BRCA2): Realigned 3’ end of *Cebus olivaceus* and *Semnopithecus hector*.

Segment 10 (CFTR): Fixed alignments at 3’ ends of sequences for *Lepilemur jamesorum*, *Lepilemur dorsalis*, *Varecia variegata*, and *Varecia rubra* (EU57281). The *V. rubra* sequence is not from Perelman et al., but there are no Perelman et al. sequences for *V. rubra* in GenBank.

Segment 11 (CHRNA1): Realigned 5’ “A” in *Lepilemur ankaranensis*. Changed end from 7430 to 7431.

Segment 12 (CNR1): Changed start from 7431 to 7432.

Segment 17 (FBN1): Changed end from 11707 to 11706.

Segment 18 (FES): Changed start from 11708 to 11707.

Segment 20 (GHR): *Otolemur garnettii* (HM761377) is 99% identical to *Sus scrofa* (EU435144) but only ~80% identical to other primate sequences. This sequence was deleted.

Segment 22 (LRPPRC_169): *Mirza zaza* (HM761170) is 99% identical to *Galeopterus variegata* (HM761070), but longer. This sequence was deleted. The 3’ end *of Cercopithecus hamlyni* was realigned. A six bp region at the 3’ end of *Macaca silenius* was also realigned.

Segment 23 (LRPPRC_171): The sequence for *Tupaia glis* (HM761069) is 96% identical to sequences for three *Pygathrix* spp. and less similar to increasingly divergent primates. This sequence was deleted.

Segment 33 (RPGRIP1): Changed end from 22037 to 22040.

Segment 34 (SGMS1): Changed start from 22038 to 22041; changed end from 22635 to 22636.

Segment 35 (SIM1): Changed start from 22636 to 22637.

Segment 37 (SMCY): Changed end from 24551 to 24550.

Segment 38 (SRY): Changed started from 24552 to 24551; changed end from 25018 to 25017.

Segment 39 (TEX2): Changed start from 25019 to 25018; changed end from 25174 to 25173.

Segment 45 (ZFY): Realigned *Hylobates lar*.

Segment 48 (BCHE): *Cercopithecus mona* sequence is 99% identical to Perelman et al.’s GenBank sequence for *Cheirogaleus medius* (HM764123), but shorter. There is no GenBank sequence for *Cercopithecus mona*.

Segment 50 (FAM123B): *Nycticebus pygmaeus* (HM762171) is 100% identical to Perelman et al.’s *Homo sapiens* sequence (HM762062), but shorter. This sequence was deleted.

Segment 51 (PNOC): *Mirza zaza* (HM759683) is 100% identical to *Alouatta palliata*. This sequence was deleted.

Segment 53 (RAB6IP1): Three *Hylobates* spp. were realigned.
