# Supplementary material for: Macroevolutionary Dynamics and Historical Biogeography of Primate Diversification Inferred from a Species Supermatrix
Source: PLoS One. 2012 Nov 16;7(11):e49521. doi: 10.1371/journal.pone.0049521 (PMC3500307; doi:10.1371/journal.pone.0049521)
Supplement: Table S3 — List of GenBank accession numbers. (DOC) [file pone.0049521.s005.doc]

Table S3. GenBank accession numbers and Ensembl/Trace files used in this study. Asterisks indicate sequences new to this study.

**1. Nuclear Genes:**

**melanocortin 1 receptor (*MC1R*):** AB296170, AB296176, AB296184, AB296192, AB296195, AB296199, AB296201, AB296204, AB296210, AB296217, AB296220-AB296222, AB296229-AB296230, AB296233-AB296234, AB296236-AB296237, AB296239, AB296241, AF326275, AY205087-AY205094, AY205096-AY205103, AY205105-AY205128, AY205130, AY205132-AY205140, AY205142-AY205143, *Microcebus murinus* Ensembl 61, NM_001164589

**prion protein gene (*PRNP*):** U08291-U08297, U08299-U08306, U08308-U08311, U75384-U75388, AY133034, AY382293, AY458651, AY765381-AY765384, AY765388-AY765390, AY765392, DQ014540, EF455529, EU311599, NM_001131072, U08312

**titin (TTN):** EU142211, EU142212, EU213056, EU213056-EU213058, EU213057, EU213058, JN632935, JN632935-JN632936, JN632936, JQ772097, JQ772097, NG_011618, NG_011618, XM_002712218, XM_002712218, XM_002806903XM_002806903, XM_002808012, XM_002808012, JX869896, new sequences (JX869897-JX869930)

**natural resistance-associated macrophage protein 1 (*NRAMP1*):** AF353027, AF353031, AF353035, AF353039, AF353049, AF353051, AF353058, AF353062-AF353063, AF353070, AF353073, AF353078-AF353079, EU342283-EU342304, HM071484-HM071485, HM071488, HM071490-HM071491

**growth hormone receptor (*GHR*):** AF332019, AF339061, AF540625, AF540627, AF540642, *Macaca mulatta* Ensembl 56, *Nomascus leucogenys* Ensembl 56, *Otolemur garnettii* Ensembl 56, JN414745-JN414753, NM_001082636, NM_001112616, X06562, XM_002815527, Pan_troglodytes Ensembl 56, HM761354-HM761496, new sequences (JX856181-JX856215)

**C-X-C chemokine receptor type 4 (*CXCR4*):** AB015943, AF051906, AF172210-AF172239, AF172241-AF172243, AF178084, AF229128, AF291672, AF452613, BC020968, EU258276, FJ638411, HQ537551, U73740, NM_001009047, NM_001112630, XM_002812445

**adora3:** AF535033, AY011226, EF052580, EU342219-EU342242, EU825254, EU835914, EU835922, FJ648394, GQ243456, GQ243469, GU230906, GU230981, GU231057, GU231096, GU231138, GU231192, GU231199, GU231217, GU231227, GU231233, GU231263, GU231305, GU231321, HM765079-HM765100, HM765102-HM765129, HM765131-HM765151, HM765153-HM765169, HM765156-HM765169, HM765171-HM765174, HM765176-HM765188, HM765190-HM765200, HM765202-HM765215, HM765218, HM765221, HM765223, HM765225, HM765230, HM765236, HM765239-HM765242, HM765246-HM765247, HM765249, HM765252-HM765254, HM765253-HM765254, NM_001082058, XM_002810439

**alpha fibrinogen (*FGA*):** DQ003394, EU342244-EU342268, EU825382, EU825408, EU825449, EU835911, EU835927, GQ243501, GU231725, GU231755, GU231867, GU231913, GU231921, GU231940, GU231981, GU232007, GU232025, GU232044, GU232069, GU232106, GU232123

**C-X-C chemokine receptor type 5 (*CXCR5*):** AF005660, AF035214, AF035216-AF035219, AF035221-AF035222, AF051905, AF075442-AF075451, AF081577, AF081579, AF105288, AF105291, AF141639, AF161887, AF161934-AF161945, AF161946, AF161948, AF161957, AF161997, AF162011, AF162014, AF162037, AF162054, AF177876, AF177877, AF177880-AF177892, AF177896-AF177899, AF177903, AF212100-AF212102, AF252551-AF252553, AF349682, AY278742, AY278745-AY278747, AY278749, AY278751-AY278752, AY344067, DQ402085, DQ499968, NM_001112629, NM_001135497

**ABO blood group protein gene (*ABO*):** AB041525, AB196679, AB196681, AB196697, AH006697, AH008219, AH008334, AH008342, AY091958, *Callithrix jacchus* Ensembl 61, *Homo sapiens* Ensembl 61, *Microcebus murinus* Ensembl 61, *Pan troglodytes* Ensembl 61, EU597772, EU597782, EU597784, EU597787, EU597791-EU597792, EU597796-EU597797, FJ377681-FJ377683, FJ377686, FJ377688-FJ377690, FJ377692, FJ377694-FJ377697, FJ377702

**epsilon globin:** AY920456-AY920457, AY920460-AY920463, AY927301, AY927305-AY927309, AY927311-AY927312, FJ769153, FJ769155, FJ769156, L25355-L25359, L25361-L25367, L25369-L25371, M15735, M36304, M81361-M81364, M93966-M93967, OC100438, U11711-U11714, U18608, U18610, U18616, U18618, U64617, U97024-U97025, U97029, V00508, X13285, XM_002822126

**von Willebrand factor (*VWF*) Intron 11:** AY434038-AY434052, AY434053, EU342339-EU342345, EU825537, EU825555, EU835917, EU835925, GQ243550, GQ243565, GU232137, GU232151, GU232227, GU232245, GU232257, GU232287, GU232317, GU232349, GU232389, GU232407, GU232423, GU232445, GU232449, GU232457

**von Willebrand factor (*VWF*) Exon 28:** AF061059, AF061064, AJ410292-AJ410302, AJ891087, AJ891092, *Homo sapiens* Ensembl 56, *Nomascus leucogenys* Ensembl 56, *Otolemur garnettii* Ensembl 56, *Pan troglodytes* Ensembl 56, JN415063, JN415069-JN415072, U31606, U31618, XM_002822802, AJ891095, new sequences (JX856250-JX856283)

**interphotoreceptor retinoid binding protein precursor (*IRBP*) Exon 1:** AF081064-AF081065, AF271414-AF271415, AF271417, AF271421, AF271423-AF271424, AJ313467-AJ313469, AJ313474, AJ313470-AJ313473, AJ313475, AJ313477-AJ313478, AY434080-AY434081, AY434084-AY434084, AY434087, *Homo sapiens* Ensembl 56, *Macaca mulatta* Ensembl 56, *Oryctolagus cuniculus* Ensembl 59, EU342269, EU342271-EU342272, EU342275-EU342277, EU342279-EU342280, EU342282, JN414800, JN414806, JN414808-JN414809, *Nomascus leucogenys* Trace Files, Z11806-Z11808, new sequences (JX856216-JX856249)

**interphotoreceptor retinoid binding protein precursor (*IRBP*) Intron 1:** U18601-U18607, U18609, U18611, U18613-U18615, U18617, U18619, U19748-U19750, U19752-U19753, U70995-U70997, AF057437-AF057443, AF057446-AF057448, AF362379-AF362381

**interphotoreceptor retinoid binding protein precursor (*IRBP*) Intron 3:** AY224272, AY224274-AY224276, AY224286, AY224288, AY224290-AY224291, AY224294, AY224301, AY224303-AY224306, AY224309, AY224311, AY224313-AY224315, AY224317-AY224322, JF293130, JF293134-JF293137, JF293139-JF293144

**Sequences for the following genes were taken from Perelman et al.’s (2011) nexus file:**

*ABCA1*, *AFF2*, *AFF2*, *APP*, *ATXN7*, *AXIN1*, *BCHE*, *BCOR*, *BDNF*, *BRCA2*, *CFTR*, *CHRNA1*, CNR1, *CREM*, *DACH1*, *DCTN2*, *DMRT1*, *S1PR1*, *ERC2*, *FAM123B*, *FBN1*, *FES*, *FOXP1*, *KCNMA1*, *LRPPRC*, *LRPPRC*, *LUC7L*, MAPKAP1, *MBD5*, *NEGR1*, *NPAS3*, *NPAS3*, *PLCB4*, *PNOC*, *POLA1*, *DENND5A*, *RAG1*, *RAG2*, *RPGRIP1*, SGMS1, *SIM1, KDM5C*, *KDM5D*, *SRY*, *TEX2*, *TTR*, *TYR*, *USH2A*, *UTY*, *ZFX*, *ZFY*, *ZIC3*

**2. Mitochondrial Genes:**

***16S rRNA***: AB049471, AB107212, AB116026, AB371085-AB371086, AB371088-AB371090, AB371092-AB371095, AB504748-AB504750, AB572419, AF072412-AF072413, AF072415-AF072418, AF072421, AF072423-AF072425, AF072427, AF072429-AF072430AF203726, AF212954, AF212956, AF212960-AF212961, AF348159, AF420037, AF420039, AF420046, AF420048-AF420049, AF424945, AF424947, AF424976, AF435501-AF435503, (AF435504, AF424977), AF435506, AF435507-AF435508, AF435517, AF435523-AF435525, AF460846, AJ309865-AJ309867, AM905040, AY011161, AY011164, AY011167, AY224244-AY224245, AY224245, AY224249-AY224250, AY224258-AY224260, AY224262-AY224263, AY224266, AY224270, AY665622-AY665627, AY665630, AY773978-AY773979, AY863425-AY863427, AY897398, DQ069713, DQ078115, DQ334826, DQ355297-DQ355302, DQ355492, EF597500, EF597502, EU004480, EU294187, EU497259, EU497272, EU497277, EU497280, EU497282, EU497286, EU497288-EU497289, EU497294, FJ640066-FJ640067, FJ769145, FJ769146-FJ769147, FJ785422-FJ785426, FJ906803, HM015213, HM070254, HQ622779, HQ622782, HQ622788, HQ622802, HQ622807, NC_001643, NC_001913, NC_001992, NC_002082-NC_002083, NC_004025, NC_005943, NC_011120, NC_014051, NC_012920, D38115, NC_011053 , U39008, U38997, U39002, U39006, U39007

***12S rRNA*:** (AY224245, AY685902), (AY685872, AY685872), (AY685877, AF424944), (AY685911, AY224264), AB371085-AB371086, AB371088-AB371090, AB371092, AB371094-AB371095, AB504748, AB504749, AB572419, AF069964-AF069965, AF069971-AF069972, AF069975, AF069979-AF069980, AF069983, AF164872, AF164874, AF164879, AF164881, AF175775-AF175776, AF175787, AF175791, AF175797, AF175799, AF212950, AF348159, AF394269-AF394272, AF420044, AF420048-AF420049, AF424947, AF424976-AF424977, AF460846, AF474186, AF474192, AF474196, AF474199, AF474201, AF474213, AF474215, AF474229, AF474234, AF474236-AF474239, AF474241, L35182, L35193, L35199, L35191, L35197, AJ309865-AJ309867, AJ429628, AM905040, AY012132, AY012135, AY043335, AY043337, AY043340, AY043342, AY192614, AY192626, AY224249, AY224250, AY224254, AY224258-AY224261, AY224265, AY224269, AY232665, AY232668, AY254047, AY582676, AY582682, AY582687, AY582694, AY582701, AY582711-AY582713, AY582717, AY585736, AY665614-AY665615, AY665618, AY665620-AY665621, AY685887, AY773973, AY862175, AY863425-AY863427, L35208, DQ069713, DQ073479-DQ073481, DQ073491, DQ073497, DQ073514, DQ073520, DQ073523, DQ355297-DQ355302, DQ355493, DQ529312, DQ529327, DQ529336, DQ529357, DQ529365, DQ529370, DQ529401, DQ529412, DQ529433, DQ529444-DQ529445, EF597500, EF597502, EU004480, EU294187, EU680968, FJ750680, FJ750684, FJ750694, FJ750702, FJ750705-FJ750706, FJ785422, FJ785424-FJ785426, FJ906803, GU068062, HM015213, HM070254, HM470206, HM470215, HM470218, HM470227, HM470229, HQ622779, HQ622782, HQ622788, HQ622802, HQ622807, HQ622808, L35202, L35204, (L35196, AF420039), NC_001643, NC_001992, NC_002082-NC_002083, NC_004025, NC_005943, NC_011120, FJ785423, JQ724861, NC_001913, NC_014051, AB371093, NC_011053, AB504750, AY012129, D38115, L35200

***ND2*:** AB286049, AB371085-AB371086, AB371088-AB371090, AB371092-AB371095, AB504748-AB504750, AB572419, AF348159, AF460846, AJ309865-AJ309867, AM905040, AY863425-AY863427, DQ069713, DQ355297-DQ355302, EF597500, EF597502, EU294187, FJ785422-FJ785426, FJ906803, HM015213, HM070254, HQ622779, HQ622782, HQ622788, HQ622802, HQ622807, HQ622808, NC_001643, NC_001913, NC_001992, NC_002082-NC_002083, NC_004025, NC_005943, NC_011120, NC_012920, NC_014051, D38115

***COI*:** AB016730-AB016732, AB286049, AB371085-AB371086, AB371088-AB371090, AB371092-AB371095, AB504748-AB504750, AB572419, AF312704, AF312708, AF348159, AF460846, AJ309865-AJ309867, AM905040, AY632376, AY671787, AY671789, AY671793, AY685713, AY685726, AY685746, AY863425-AY863427, AY972666, AY972678, AY972682, AY972695, AY972781, AY972790, AY972807, AY972809, D38115, DQ069713, DQ355297-DQ355302, EF568610, EF568626, EF597500, EF597502, EU179510-EU179513, EU185724, EU185726, EU185730, EU294187, FJ402884, FJ713433-FJ713434, FJ750674, FJ750677, FJ785422-FJ785426, FJ906803, GQ144555, GQ144564, GQ144597, GQ259899, GQ259901-GQ259903, GU068066, HM015213, HM070254, HQ005472, HQ005474, HQ005478, HQ005481, HQ005483, HQ005485-HQ005486, HQ005488, HQ622779, HQ622782, HQ622788, HQ622802, HQ622807, NC_001643, NC_001913, NC_001992, NC_002082-NC_002083, NC_005943, NC_011120, NC_012920, NC_014051

***COII*:** AB286049, AB371085-AB371086, AB371088-AB371090, AB371092-AB371095, AB504748-AB504750, AB572419, AF054300, AF081041-AF081044, AF081046, AF181086, AF216226, AF216232, AF216234, AF216241, AF216250, AF348159, AF396460, AF460846, AJ309865-AJ309867, L22774, AM905040, AY118180, AY118192, AY118195, AY321459, AY434077, AY515558-AY515559, AY569193, AY569204-AY569205, AY584482-AY584485, AY585740, AY685761, AY685781, AY685786, AY686129, AY686145-AY686146, AY686150, AY863425-AY863426, AY863427, D38115, DQ069713, DQ118291, DQ355297-DQ355302, DQ381471, EF065250, EF065254, EF065268, EF122246, EF597500, EF597502, EF999917-EF999918, EU294187, EU810352, EU810354, EU825472, EU825481, EU825511, EU835915, EU835931, FJ713417, FJ713422, FJ750644, FJ750650, FJ785422-FJ785426, FJ906803, GU212711, GU326978, GU326994, GU327017, GU327028, GU327034, GU327056, GU327062, GU327080, GU327096, GU327105, GU327108, GU327111, GU327128, GU327136, HM015213, HM057579, HM057595, HM057602, HM070254, HM631738, HQ005472, HQ005474, HQ005478, HQ005481, HQ005483, HQ005485-HQ005486, HQ005488, HQ622779, HQ622782, HQ622788, HQ622802, HQ622807, L22780, L22782, L22785, M74004, M74006, NC_001643, NC_001913, NC_001992, NC_002082-NC_002083, NC_005943, NC_011120, NC_012920, NC_014051

***COIII*:** AB286049, AB371085-AB371086, AB371088-AB371090, AB371092-AB371095, AB504748-AB504750, AB572419, AF224525, AF224531, AF224563, AF224571, AF224576, AF224581, AF224583, AF224590, AF224598-AF224601, AF224617, AF224620-AF224621, AF224624, AF348159, AF460846, AJ309865-AJ309867, AM905040, AY582554, AY582556, AY582560, AY582562, AY582572, AY582585, AY582588, AY582594, AY582601, AY582604, AY582610, AY582620, AY582628, AY582633, AY582636, AY582647, AY582659, AY582662, AY582667, AY685792, AY685795, AY685808, AY685818, AY685828, AY863425-AY863427, DQ069713, DQ355297-DQ355302, DQ529602, DQ529617, DQ529626, DQ529647, DQ529655, DQ529660, DQ529678, DQ529693, DQ529704, DQ529725, DQ529736-DQ529737, DQ534982, DQ535002, DQ535017, DQ856049, DQ856111, DQ856115, EF175230, EF175253, EF552592-EF552593, EF552599, EF552609-EF552610, EF597500, EF597502, EU294187, EU779960-EU779961, EU779971, FJ785422-FJ785426, FJ906803, HM015213, HM070254, HQ622779, HQ622782, HQ622788, HQ622802, HQ622807, HQ622808, NC_001643, NC_001913, NC_001992, NC_002082-NC_002083, NC_004025, NC_005943, NC_011120, NC_012920, NC_014051, D38115

***CYTB*:** AB075974, AB286049, AB371085-AB371086, AB371088-AB371090, AB371092-AB371095, AB504748-AB504750, AB572419, AF001931, AF020411, AF020588, (AF044061, AF245089), AF044062, AF081048, AF081050, AF175846, AF175858, AF212964, AF212966, AF212970, AF245047-AF245052, AF271409, AF289988-AF289989, AF294618, AF294620, AF294622, AF294626, AF295577-AF295578, AF295580, AF295583, AF301612, AF348159, AF350392, AF350395, AF350399, AF350404, AF460846, AF524885, AF524887, AF524890, AJ304499, AJ309865-AJ309867, AJ315389, AJ421451, AJ428969, AJ428980, AJ428986, AJ489759, AJ489759, AM905040, AY065881, AY065883-AY065884, AY065887, AY065907, AY151109, AY204830, AY204831-AY204833, AY226183, AY226185, AY226189, AY232661, AY321641, AY374345, AY374378, AY441446-AY441448, AY441450, AY441455-AY441456, AY441461, AY441466-AY441470, AY441472, AY441474, AY441477, AY519449, AY519451-AY519452, AY519462, AY671799, AY685858, AY687891, AY738634, AY863425-AY863427, AY998832, D38115, DQ069713, DQ098872-DQ098873, DQ109034, DQ143883, DQ234881-DQ234882, DQ234886, DQ234888-DQ234889, DQ234894, DQ337707, DQ355297-DQ355302, DQ444302, DQ529457, DQ679774, DQ859973, DQ859977, EF065195, EF065204, EF065211, EF103293, EF103295-EF103296, EF103319, EF103325-EF103326, EF103330, EF465126, EF465147, EF597500, EF597502, EF686711, EU004473, EU004475, EU004480, EU200438, EU232702, EU232708, EU232712, EU294187, EU560411, EU560414, EU810362, EU825334, EU825337, EU835932, EU885440, EU885449, EU885452, EU885476, FJ460174, FJ529066, FJ529104, FJ529106, FJ531641, FJ531658, FJ531667-FJ531668, FJ614306, FJ614371, FJ785422-FJ785426, FJ906803, GQ243489, GQ243499, GU321248, GU321256, GU321287, GU321290, GU327166, GU327180, GU327216, GU327241, GU327251, GU327262-GU327263, GU327294, GU327297, GU327306, GU327319, GU327329, GU327353, GU327355, GU594996, HM015213, HM070254, HM071114, HM071116-HM071118, HM071121, HM071123, HM115975, HM222708, HM367997, HM368005, HM368012, HM368020, HM368035, HM368046, HM368074, HM368077-HM368078, HQ005492, HQ005494, HQ005498, HQ005503, HQ005506, HQ005508, HQ622779, HQ622782, HQ622788, HQ622802, HQ622807, HQ731525, HQ731529, HQ731535, HQ731538, HQ731548, L02753, L44587, L44589-L44591, NC_001643, NC_001913, NC_001992, NC_002082-NC_002083, NC_005943, NC_011120, NC_012920, NC_014051, AF081052, AY441463, DQ109021, Q10900, U53576-U53577

***ND3*:** AB286049, AB371085-AB371086, AB371088-AB371090, AB371092-AB371095, AB504748-AB504750, AB572419, AF091409-AF091410, AF091412-AF091413, AF091422, AF091424, AF091427-AF091429, AF224525, AF224531, AF224563, AF224571, AF224576, AF224581, AF224583, AF224590, AF224598-AF224601, AF224617, AF224620-AF224621, AF224624, AF348159, AF460846, AJ309865-AJ309867, AM905040, AY582554, AY582556, AY582560, AY582562, AY582572, AY582585, AY582588, AY582594, AY582601, AY582604, AY582610, AY582620, AY582628, AY582633, AY582636, AY582647, AY582659, AY582662, AY582667, AY860949, AY863425-AY863427, AY961034, DQ069713, DQ355297-DQ355302, DQ529602, DQ529617, DQ529626, DQ529647, DQ529655, DQ529660, DQ529678, DQ529693, DQ529704, DQ529725, DQ529736, DQ529737, DQ534982, DQ535002, DQ535017, DQ856049, DQ856111, DQ856115, EF175230, EF175253, EF552592-EF552593, EF552599, EF552609-EF552610, EF597500, EF597502, EU294187, EU580047, EU580053, EU580054, EU580056, EU580061-EU580062, EU580067, EU580069, EU580072, EU580075, EU580078, EU580080, EU580082, EU779960-EU779961, EU779971, FJ785422-FJ785426, FJ906803, HM015213, HM070254, HQ622779, HQ622782, HQ622788, HQ622802, HQ622807, NC_001643, NC_001913, NC_001992, NC_002082-NC_002083, NC_004025, NC_005943, NC_011120, NC_012920, NC_014051, D38115, U92955, U92960, U92968, U92970

***ND4*:** AB286049, AB371085-AB371086, AB371088-AB371090, AB371092-AB371095, AB504748-AB504750, AB572419, AF053685-AF053686, AF053688-AF053691, AF053694, AF091409-AF091410, AF091412-AF091413, AF091422, AF091424, AF091426-AF091427, AF091429, AF224525, AF224531, AF224563, AF224571, AF224576, AF224581, AF224583, AF224590, AF224598-AF224601, AF224617, AF224620-AF224621, AF224624, AF348159, AF460846, AJ309865-AJ309867, AM905040, AY488131-AY488132, AY582554, AY582556, AY582560, AY582562, AY582572, AY582585, AY582588, AY582594, AY582601, AY582604, AY582610, AY582620, AY582628, AY582633, AY582636, AY582647, AY582659, AY582662, AY582667, AY860949, AY863425-AY863427, AY961034, D85270, D85280, D85282-D85284, D85286, D85291, DQ069713, DQ355297-DQ355302, DQ529602, DQ529617, DQ529626, DQ529647, DQ529655, DQ529660, DQ529678, DQ529693, DQ529704, DQ529725, DQ529736-DQ529737, DQ534982, DQ535002, DQ535017, DQ856049, DQ856111, DQ856115, EF175230, EF175253, EF552592-EF552593, EF552599, EF552609-EF552610, EF597500, EF597502, EU294187, EU580047, EU580053, EU580054, EU580056, EU580061-EU580062, EU580067, EU580069, EU580072, EU580075, EU580078, EU580080, EU580082, EU779960, EU779961, EU779971, EU885810, FJ531515, FJ785422-FJ785426, FJ881876, FJ906803, GU068080, HM015213, HM070254, HQ622779, HQ622782, HQ622788, HQ622802, HQ622807, NC_001643, NC_001913, NC_001992, NC_002082-NC_002083, NC_004025, NC_005943, NC_011120, NC_012920, NC_014051, D38115, U92955, U92960, U92968, U92970

***ND4L*:** AB286049, AB371085-AB371086, AB371088-AB371090, AB371092-AB371095, AB504748-AB504750, AB572419, AF091409-AF091410, AF091412-AF091413, AF091422, AF091424-AF091427, AF091429, AF224525, AF224531, AF224563, AF224571, AF224576, AF224581, AF224583, AF224590, AF224598-AF224601, AF224617, AF224620-AF224621, AF224624, AF348159, AF460846, AJ309865-AJ309866, AJ309867, AM905040, AY582554, AY582556, AY582560, AY582562, AY582572, AY582585, AY582588, AY582594, AY582601, AY582604, AY582610, AY582620, AY582628, AY582633, AY582636, AY582647, AY582659, AY582662, AY582667, AY860949, AY863425-AY863427, AY961034, DQ069713, DQ355297-DQ355302, DQ529602, DQ529617, DQ529626, DQ529647, DQ529655, DQ529660, DQ529678, DQ529693, DQ529704, DQ529725, DQ529736-DQ529737, DQ534982, DQ535002, DQ535017, DQ856049, DQ856111, DQ856115, EF175230, EF175253, EF552592, EF552593, EF552599, EF552609-EF552610, EF597500, EF597502, EU294187, EU580047, EU580053-EU580056, EU580061-EU580062, EU580067, EU580069, EU580072, EU580075, EU580078, EU580080, EU580082, EU779960, EU779961, EU779971, FJ785422-FJ785426, FJ906803, HM015213, HM070254, HQ622779, HQ622782, HQ622788, HQ622802, HQ622807, HQ622808, NC_001643, NC_001913, NC_001992, NC_002082-NC_002083, NC_004025, NC_005943, NC_011120, NC_012920, NC_014051, EU580061, U92955, U92960, U92968, U92970
