# Supplementary material for: Macroevolutionary Dynamics and Historical Biogeography of Primate Diversification Inferred from a Species Supermatrix
Source: PLoS One. 2012 Nov 16;7(11):e49521. doi: 10.1371/journal.pone.0049521 (PMC3500307; doi:10.1371/journal.pone.0049521)
Supplement: Table S4 — List of genes that were included in eight different mcmctree partitions. (DOCX) [file pone.0049521.s006.docx]

Table S4. List of genes that were included in eight different partitions for mcmctree analyses.

Autosomal Rate1: ush2a, mbd2, sgms1, sim1, negr1, bdnf, npas3, kcnma1, atxn7, fbn1, CXCR4, TTN, tex2, mapkap1, dmrt1

Autosomal Rate2: app, plcb4, dach1, GHRmeredith, dctn2, rag1, axin1, rpgrip1, bche, edg1, npas3_2, rag2, foxp1, NRAMP, cnr1

Autosomal Rate3: luc71, lrpprc_169, adora3, tyr, erc2, lrpprc_171, crem, CXCR5, Prion, FGA, cftr, rab6ip1, GHRpereoman, brca2, irbpEXON

Autosomal Rate4: EpsilonGlobin, chrna1, VWFexon, VWFintron, fes, pnoc, IRBPintron3, ttr, MCR1, abca1, ABO, IRBPintron1

Mitochondrial Protein-Coding: COB, COI, COII, COIII, ND2, ND3, ND4, ND4L

Mitochondrial RNA = 12S rRNA, 16S rRNA

X-linked: aff2, aff22, bcor, fam123b, pola1, smcx, zfx, zic3

Y-linked: smcy, sry, uty, zfy
