# Supplementary material for: Macroevolutionary Dynamics and Historical Biogeography of Primate Diversification Inferred from a Species Supermatrix
Source: PLoS One. 2012 Nov 16;7(11):e49521. doi: 10.1371/journal.pone.0049521 (PMC3500307; doi:10.1371/journal.pone.0049521)
Supplement: Text S1 — Compendium of primate species that have been recognized subsequent to Groves [6]. (DOCX) [file pone.0049521.s007.docx]

Text S1. List of 74 primate species not included in Groves 2005 [1].

Cheirogaleidae (+14). Groves [1] recognized eight species of *Microcebus*. Additional *Microcebus* species have been recognized by Kappeler et al. [2], Andriantompohavana et al. [3], Louis et al. [4,5], Oliveri et al. [6], Mittermeier et al. [7], Radespiel et al. [8,9], and Weisrock et al. [10]. In addition to the 16 species enumerated by Mittermeier et al. [7] we recognized the following eight taxa: *M. margotmarshae* [5,9] = sp. nova #5 of Andriantompohavana [3]; *M. arnholdi* [5,9] = *M*. sp. nova #6 of Andriantompohvana [3] = *M*. sp. Montagne e’Ambre of Weisrock et al. [10]; *M. gerpi* [9]; *M*. sp. nova 1 [4]; *M*. sp. nova 3 [4]; *M*. sp. nova 3 [8] from Makira, 003y06hely from Anjiahely; *M*. sp. Marolambo [9,10]; and *M*. sp. Manantantely/Ivorona [9,10]. The number of species in *Cheirogaleus* has been reduced from seven to four [11]. One species of *Mirza* (*M. zaza*) was described by Kappeler et al. [2].

Lemuridae (+3). Groves recognized 11 species of *Eulemur*. Mittermeier et al. [7] recognized 12 species of *Eulemur* (*E. rufifrons*, *E. flavifrons*). *E. rufifrons* was split apart from *E. rufus*, *E. macaco flavifrons* was elevated to full species status, and *E. albocollaris* was recognized as a junior synonym of *E*. *cinereiceps*. Two additional species of *Hapalemur* (*H. meridionalis*, *H. gilberti*) were also recognized by Mittermeier et al. [7].

Lepilemuridae (+17). Groves [1]recognized eight *Lepilemur* species, whereas Mittermeier et al. [7] recognized 25 species of *Lepilemur*.

Indriidae (+6): Groves [1] recognized three species of *Avahi*. Mittermeier et al. [7] recognized nine species. Groves [1] recognized seven species of *Propithecus*. Mittermeier et al. [7] elevated *P. diadema candidus* and *P. deckenii coronatus* to full species. Subsequently, Rumpler et al. [12] modified Mittermeier et al.’s [7] classification by lumping *P. deckenii* and *P.* *coronatus* into *P. verreauxi* based on mitochondrial and chromosome data.

Lorisidae (+2). Groves [1] recognized three species of *Nycticebus*, but Nekaris et al. [13] recognized two additional species of *Nycticebus* (*N. javanicus*, *N. menagensis*).

Tarsiidae (+6). Groves recognized seven species of *Tarsius*. Groves and Shekelle [14] divided *Tarsius* into three genera (*Carlito*, *Cephalopachus*, *Tarsius*). *Carlito* and *Cephalopachus* are both monotypic, but *Tarisus* includes nine named species, two species that are currently in the process of description, and up to six or more additional species [14] Table I in Shekelle et al. [15] lists 16 different acoustic forms of *Tarsius*, each of which may represent a distinct species [14]. We provisionally recognize that there are at least 13 tarsiers (1 *Carlito*, 1 *Cephalopachus*, 11 *Tarsius*), and that this number is likely to increase with additional fieldwork and molecular studies.

Cebidae (+5). *Callithrix Mico rondoni* was described by Ferrari et al. [16]. *C. rondoni* was previously synonymized with *C. emiliae* [16]. Matauschek et al. [17] proposed a revised taxonomy for five species (*tripartitus, melanoleucas, graellsi, nigricollis, fuscicollis*) in the *Saguinus* *nigricollis* species group. Their proposed classification increases the number of species from five to eight including subdividing *fuscicollis* into multiple species. *Cebus querozi* is a new species [18].

Pitheciidae (+3). *Callicebus caquetensis* was described by Defler et al. [19]. *Cacajao ayresi* and *C. hosomi* were described by Boubli et al. [20].

Aotidae (+5). Defler and Bueno [21] recognized seven species in the gray-necked group of *Aotus* based on karyotypes. *A. brumbacki*, *A. griseimembra*, *A. zonalis*, and *A. jorgehernandezi* are in addition to taxa recognized as species by Groves [1]. We also recognized *A. infulatus* as a distinct species following Menezes et al. [22].

Cercopithecidae (+9): *Macaca munzala* was described by Sinha et al. [23]. We also recognized *M. brunnescens* following Riley [24]. Other new cercopithecids include *Rungwecebus kipunji* [25], *Rhinopithecus strykeri* [26], and five additional species of *Presbytis* (*P. siberu, P. federicae, P. mitrata, P. bicolor, P. sumatrana*) [27].

Hylobatidae (+4): Thinh et al. [28] recognized *Hoolock leuconedys*, *Nomascus nasutus*, *Hylobates funereus*, and *H. abbotti* as distinct species.

**References**

1. Groves CP (2005) Order Primates. In: Wilson DE, Reeder DM, eds. Mammal species of the world: a taxonomic and geographic reference, third edition. Baltimore: Johns Hopkins University Press. pp. 111-184.

2. Kappeler PM, Rasoloarison RM, Razafimanantsoa L, Walter L, Roos C (2005) Morphology, behavior and molecular evolution of giant mouse lemurs (*Mirza* spp.) Gray, 1870, with description of a new species. Primate Rep 71: 3-26.

3. Andriantompohavana R, Zaonarivelo JR, Engberg SE, Randriamapionona R, McGuire SM, et al. (2006) Mouse lemurs of northwestern Madagascar with a description of a new species at Lokobe Special Reserve. Occ Pap Mus Texas Tech Univ 259: 1-24.

4. Louis EE Jr, Engberg SE, Lei R, Geng H, Sommer JA, et al (2006) Molecular and morphological analyses of the sportive lemurs (Family Megaladapidae: Genus *Lepilemur*) reveals 11 previously unrecognized species. Spec Pub Mus Texas Tech Univ 49: 1-47.

5. Louis EE Jr, Engberg SE, McGuire SM, McCormick MJ, Randriamampionona R, et al (2008) Revision of the mouse lemurs, Microcebus (Primates, Lemuriformes), of northern and northwestern Madagascar with descriptions of two new species at Montagne d’Ambre National Park and Antafondro Classified Forest. Primate Cons 23: 19-38.

6. Olivieri G, Zimmermann E, Randrianambinina B, Rasoloharijaona S, Rakotondravony D, et al. (2007) The ever-increasing diversity in mouse lemurs: three new species in north and northwestern Madagascar. Mol Phylogenet Evol 43: 309-327.

7. Mittermeier RA, Ganzhorn JU, Konstant WR, Glander K, Tattersall I, et al (2008) Lemur diversity in Madagascar. Int J Primatol 29: 1607-1656.

8. Radespiel U, Olivieri G, Rasolofoson DW, Rakotondratsimba G, Rakotonirainy O, et al. (2008) Exceptional diversity of mouse lemurs (*Microcebus* spp.) in the Makira region with the description of one new species. Am J Primatol 70: 1033-1046.

9. Radespiel U, Ratsimbazafy JH, Rasoloharijaona S, Raveloson H, Andriaholinirina N, et al. (2012) First indications of a highland specialist among mouse lemurs (*Microcebus* spp.) and evidence for a new mouse lemur species from eastern Madagascar. Primates 53: 153-170.

10. Weisrock DW, Rasoloarison RM, Fiorentino I, Ralison JM, Goodman SM, et al. (2010) Delimiting species without nuclear monophyly in Madagascar's mouse lemurs. PLoS ONE 5: e9883.

11. Groeneveld LF, Blanco MB, Raharison JL, Rahalinarivo B, Rasoloarison RM, et al. (2010) MtDNA and nDNA corroborate existence of sympatric dwarf lemur species at Tsinjoarivo, eastern Madagascar. Mol Phylogenet Evol 55: 833-845.

12. Rumpler Y, Hauwy M, Fausser JL, Roos C, Zaramody A, et al. (2011) Comparing chromosomal and mitochondrial phylogenies of the Indriidae (Primates, Lemuriformes). Chromosome Res 19: 209-224.

13. Nekaris KAI, Blackham GV, Nijman V (2008) Conservation implications of low encounter rates of five primate species (*Nycticebus* spp.) in Asia. Biodivers Conserv 17: 733-747.

14. Groves C, Shekelle M (2010) The genera and species of Tarsiidae. Int J Primatol 31: 1071-1082.

15. Shekelle M, Meier R, Wahyu I, Wirdateti, Ting N (2010) Molecular phylogenetics and chronometrics of Tarsiidae based on 12S mtDNA haplotypes: evidence for Miocene origins of crown tarsiers and numerous species within the Sulawesian clade. Int J Primatol 31: 1083-1106.

16. Ferrari SF, Sena L, Schneider MPC, Silva JS Jr (2010) Rondon’s marmoset, *Mico rondoni* sp. n., from southwestern Brazilian Amazonia. Int J Primatol 31: 693-714.

17. Matauschek C, Roos C, Heymann EW (2011) Mitochondrial phylogeny of tamarins (*Saguinus*, Hoffmannsegg 1807) with taxonomic and biogeographic implications for the *S. nigricollis* species group. Am J Phys Anthropol 144: 564-574.

18. Pontes ARM, Malta A, Asfora PH (2006) A new species of capuchin monkey, genus *Cebus* Erxleben (Cebidae, Primates): found at the very brink of extinction in the Pernambuco Endemism Centre. Zootaxa 1200: 1-12.

19. Defler TR, Bueno ML, Garcia J (2010) *Callicebus caquetensis*: a new and critically endangered titi monkey from Southern Caqueta, Colombia. Primate Cons 25: 1-9.

20. Boubli JP, da Silva MN, Amado MV, Hrbek T, Pontual FB, et al. (2008) A taxonomic reassessment of *Cacajao melanocephalus* Humboldt (1811), with the description of two new species. Int J Primatol 29: 723-741.

21. Defler TR, Bueno ML (2007) *Aotus* diversity and the species problem. Primate Cons 22: 55-70.

22. Menezes AN, Bonvicino CR, Seuanez HN (2010) Identification, classification and evolution of owl monkeys (*Aotus*, Illiger 1811). BMC Evol Biol 10: 248.

23. Sinha A, Datta A, Madhusudan MD, Mishra C (2005). *Macaca munzala*: A new species from western Arunachal Pradesh, northeastern India. Int J Primatol 26: 977**–**989.

24. Riley EP (2010) The endemic seven: four decades of research on the Sulawesi macaques. Evol Anthropol 19: 22-36.

25. Zinner D, Arnold ML, Roos C (2009) Is the new primate genus *Rungwecebus* a baboon? PLoS ONE 4: e4859.

26. Geissmann T, Lwin N, Aung SS, Aung TH, Aung ZM, et al. (2011) A new species of snub-nosed monkey, genus *Rhinopithecus* Milne-Edwards, 1872 (Primates, Colobinae), from northern Kachin State, northeastern Myanmar. Am J Primatol 73: 96-107.

27. Meyer D, Rinaldi ID, Ramlee H, Perwitasari-Farajallah D, Hodges JK, et al. (2011) Mitochondrial phylogeny of leaf monkeys (genus *Presbytis*, Eschscholtz, 1821) with implications for taxonomy and conservation. Mol Phylogenet Evol 59: 311-319.

28. Thinh VN, Mootnick AR, Geissmann T, Li M, Ziegler T, et al. (2010) Mitochondrial evidence for multiple radiations in the evolutionary history of small apes. BMC Evol Biol 10: 74.
