# Supplementary material for: Macroevolutionary Dynamics and Historical Biogeography of Primate Diversification Inferred from a Species Supermatrix
Source: PLoS One. 2012 Nov 16;7(11):e49521. doi: 10.1371/journal.pone.0049521 (PMC3500307; doi:10.1371/journal.pone.0049521)
Supplement: Text S2 — Timetrees and RAxML phylograms in Newick format. 1. Newick timetree with autocorrelated rates and hard-bounded constraints. 2. Newick timetree with autocorrelated rates and soft-bounded constraints. 3. Newick timetree with independent rates and hard-bounded constraints. 4. Newick timetree with independent rates and soft-bounded constraints. 5. RAxML phylogram based on the 61199 bp concatenation of 69 nuclear and ten mitochondrial loci. 6. RAxML phylogram based on ten mitochondrial genes. 7. RAxML phylogram based on 69 nuclear genes. (DOCX) [file pone.0049521.s008.docx]

Text S2. Timetrees and RAxML phylograms in Newick format.

1. Newick timetree with autocorrelated rates and hard-bounded constraints. One unit = 100 million years.

(((Galeopterus_variegatus: 0.094249, Cynocephalus_volans: 0.094249): 0.567312, (((((Galago_thomasi: 0.181653, (Galago_demidoff: 0.174878, ((Galago_matschiei: 0.050230, (Galago_moholi: 0.009526, (Galago_gallarum: 0.007746, Galago_senegalensis: 0.007746): 0.001780): 0.040704): 0.104010, ((Galago_orinus: 0.091635, (Galago_granti: 0.023193, Galago_zanzibaricus: 0.023193): 0.068443): 0.046511, ((Otolemur_monteiri: 0.097561, (Otolemur_crassicaudatus: 0.037117, Otolemur_garnettii: 0.037117): 0.060444): 0.004800, (Galago_gabonensis: 0.006769, Galago_alleni: 0.006769): 0.095592): 0.035785): 0.016093): 0.020638): 0.006775): 0.045775, Euoticus_elegantulus: 0.227428): 0.146053, (((Loris_lydekkerianus: 0.004987, Loris_tardigradus: 0.004987): 0.260319, (Nycticebus_pygmaeus: 0.061218, (Nycticebus_menagensis: 0.039322, (Nycticebus_javanicus: 0.005626, (Nycticebus_bengalensis: 0.003721, Nycticebus_coucang: 0.003721): 0.001905): 0.033696): 0.021896): 0.204089): 0.080624, (Perodicticus_potto: 0.288524, (Arctocebus_calabarensis: 0.009421, Arctocebus_aureus: 0.009421): 0.279103): 0.057407): 0.027550): 0.159386, (Daubentonia_madagascariensis: 0.504863, (((Varecia_rubra: 0.008738, Varecia_variegata: 0.008738): 0.219145, ((Lemur_catta: 0.090545, (Prolemur_simus: 0.083355, (Hapalemur_aureus: 0.053212, (Hapalemur_occidentalis: 0.008426, (Hapalemur_alaotrensis: 0.002663, (Hapalemur_griseus: 0.001403, Hapalemur_meridionalis: 0.001403): 0.001260): 0.005763): 0.044786): 0.030143): 0.007190): 0.106820, (((((Eulemur_rufifrons: 0.020598, (Eulemur_fulvus: 0.013512, (Eulemur_albifrons: 0.011553, Eulemur_sanfordi: 0.011553): 0.001958): 0.007087): 0.007581, ((Eulemur_cinereiceps: 0.011954, Eulemur_collaris: 0.011954): 0.014061, Eulemur_rufus: 0.026016): 0.002164): 0.028171, Eulemur_mongoz: 0.056350): 0.008869, ((Eulemur_macaco: 0.011324, Eulemur_flavifrons: 0.011324): 0.046492, Eulemur_coronatus: 0.057816): 0.007403): 0.008732, Eulemur_rubriventer: 0.073952): 0.123414): 0.030518): 0.106967, (((Phaner_pallescens: 0.253929, ((((Lepilemur_aeeclis: 0.032805, (Lepilemur_randrianasoloi: 0.024193, (Lepilemur_hubbardorum: 0.015294, Lepilemur_ruficaudatus: 0.015294): 0.008899): 0.008612): 0.018637, (Lepilemur_leucopus: 0.008988, Lepilemur_petteri: 0.008988): 0.042454): 0.014594, ((Lepilemur_microdon: 0.035203, (Lepilemur_otto: 0.013161, (Lepilemur_grewcockorum: 0.009997, Lepilemur_edwardsi: 0.009997): 0.003164): 0.022043): 0.013692, (Lepilemur_septentrionalis: 0.027823, ((Lepilemur_ankaranensis: 0.008708, (Lepilemur_tymerlachsoni: 0.007884, Lepilemur_milanoii: 0.007884): 0.000824): 0.009887, ((Lepilemur_mittermeieri: 0.014705, (Lepilemur_sahamalazensis: 0.008934, Lepilemur_ahmansoni: 0.008934): 0.005772): 0.002654, Lepilemur_dorsalis: 0.017359): 0.001236): 0.009228): 0.021073): 0.017140): 0.035472, (Lepilemur_seali: 0.064254, (Lepilemur_wrighti: 0.040775, (Lepilemur_fleuretae: 0.027665, (Lepilemur_mustelinus: 0.015493, (Lepilemur_betsileo: 0.010335, Lepilemur_jamesorum: 0.010335): 0.005158): 0.012172): 0.013110): 0.023478): 0.037254): 0.152422): 0.040685, ((Allocebus_trichotis: 0.180897, ((Mirza_coquereli: 0.007865, Mirza_zaza: 0.007865): 0.138634, ((Microcebus_griseorufus: 0.051099, Microcebus_murinus: 0.051099): 0.022461, (((Microcebus_macarthurii: 0.026669, Microcebus_sp_nova3Radespiel08: 0.026669): 0.016444, (Microcebus_danfossi: 0.032273, (Microcebus_ravelobensis: 0.007859, Microcebus_bongolavensis: 0.007859): 0.024414): 0.010840): 0.015914, ((((Microcebus_rufus: 0.032609, ((Microcebus_sambiranensis: 0.011157, Microcebus_arnholdi: 0.011157): 0.015865, (Microcebus_mamiratra: 0.012137, Microcebus_margotmarshae: 0.012137): 0.014885): 0.005587): 0.005469, (((Microcebus_myoxinus: 0.016797, Microcebus_berthae: 0.016797): 0.011792, Microcebus_lehilahytsara: 0.028590): 0.007021, Microcebus_mittermeieri: 0.035611): 0.002467): 0.005289, (Microcebus_simmonsi: 0.038540, (((Microcebus_jollyae: 0.000511, (Microcebus_sp_nova1Louis06: 0.000289, Microcebus_sp_nova3Louis06: 0.000289): 0.000221): 0.029231, (Microcebus_gerpi: 0.010315, Microcebus_spDWW2010aMarolambo: 0.010315): 0.019427): 0.004308, Microcebus_spDWW2010aManantantel: 0.034049): 0.004491): 0.004826): 0.005833, Microcebus_tavaratra: 0.049199): 0.009828): 0.014533): 0.072939): 0.034398): 0.056430, (Cheirogaleus_sibreei: 0.116011, (Cheirogaleus_crossleyi: 0.093055, (Cheirogaleus_major: 0.063992, Cheirogaleus_medius: 0.063992): 0.029063): 0.022956): 0.121316): 0.057287): 0.029412, (Indri_indri: 0.212060, (((((Avahi_ramanantsoavani: 0.013607, Avahi_meridionalis: 0.013607): 0.006969, (Avahi_peyrierasi: 0.007417, Avahi_betsilio: 0.007417): 0.013159): 0.013974, Avahi_laniger: 0.034550): 0.044520, (Avahi_occidentalis: 0.019273, (Avahi_cleesei: 0.016883, Avahi_unicolor: 0.016883): 0.002389): 0.059797): 0.095449, ((Propithecus_perrieri: 0.033641, (Propithecus_edwardsi: 0.018394, Propithecus_diadema: 0.018394): 0.015247): 0.039388, ((Propithecus_tattersalli: 0.019016, Propithecus_coquereli: 0.019016): 0.016834, Propithecus_verreauxi: 0.035850): 0.037180): 0.101490): 0.037541): 0.111967): 0.010823): 0.170013): 0.028005): 0.094794, (((Tarsius_dentatus: 0.073761, ((Tarsius_wallacei: 0.023381, Tarsius_tarsier: 0.023381): 0.024433, (Tarsius_sangirensis: 0.037029, Tarsius_lariang: 0.037029): 0.010784): 0.025948): 0.175698, (Tarsius_bancanus: 0.060703, Tarsius_syrichta: 0.060703): 0.188756): 0.336564, ((((((Cacajao_calvus: 0.034422, (Cacajao_melanocephalus: 0.010613, (Cacajao_hosomi: 0.002114, Cacajao_ayresi: 0.002114): 0.008499): 0.023810): 0.036360, ((Chiropotes_israelita: 0.011654, Chiropotes_chiropotes: 0.011654): 0.021098, Chiropotes_utahicki: 0.032752): 0.038030): 0.072241, (Pithecia_pithecia: 0.038859, (Pithecia_monachus: 0.005128, Pithecia_irrorata: 0.005128): 0.033731): 0.104164): 0.064111, ((Callicebus_lugens: 0.032802, Callicebus_torquatus: 0.032802): 0.046558, (((Callicebus_personatus: 0.020245, Callicebus_coimbrai: 0.020245): 0.016016, Callicebus_nigrifrons: 0.036260): 0.035504, ((Callicebus_cupreus: 0.022518, (Callicebus_brunneus: 0.020697, (Callicebus_moloch: 0.009478, Callicebus_hoffmannsi: 0.009478): 0.011220): 0.001821): 0.009736, (Callicebus_caligatus: 0.005710, Callicebus_donacophilus: 0.005710): 0.026545): 0.039510): 0.007596): 0.127773): 0.033178, ((((Alouatta_pigra: 0.025391, (Alouatta_palliata: 0.004361, Alouatta_coibensis: 0.004361): 0.021030): 0.016791, ((Alouatta_caraya: 0.034425, (Alouatta_sara: 0.030768, (Alouatta_nigerrima: 0.005735, (Alouatta_seniculus: 0.000521, Alouatta_macconnelli: 0.000521): 0.005215): 0.025033): 0.003657): 0.003136, (Alouatta_guariba: 0.028204, Alouatta_belzebul: 0.028204): 0.009358): 0.004621): 0.130482, (((Brachyteles_hypoxanthus: 0.032681, Brachyteles_arachnoides: 0.032681): 0.082851, ((Lagothrix_cana: 0.005719, (Lagothrix_lagotricha: 0.004092, Lagothrix_poeppigii: 0.004092): 0.001627): 0.004840, Lagothrix_lugens: 0.010558): 0.104973): 0.017714, ((Ateles_fusciceps: 0.025320, (Ateles_hybridus: 0.024601, (Ateles_belzebuth: 0.018138, Ateles_geoffroyi: 0.018138): 0.006463): 0.000719): 0.010185, (Ateles_paniscus: 0.014374, (Ateles_marginatus: 0.005236, Ateles_chamek: 0.005236): 0.009138): 0.021132): 0.097741): 0.039418): 0.055489, ((((Saimiri_ustus: 0.013014, (Saimiri_sciureus: 0.008274, Saimiri_oerstedii: 0.008274): 0.004741): 0.006812, Saimiri_boliviensis: 0.019826): 0.160509, (((Cebus_libidinosus: 0.003468, Cebus_apella: 0.003468): 0.018589, (Cebus_xanthosternos: 0.019813, Cebus_robustus: 0.019813): 0.002244): 0.045377, (Cebus_capucinus: 0.018379, ((Cebus_kaapori: 0.006491, Cebus_olivaceus: 0.006491): 0.010234, Cebus_albifrons: 0.016725): 0.001654): 0.049055): 0.112900): 0.025303, ((((Saguinus_fuscicollis: 0.028238, ((Saguinus_melanoleucus: 0.019685, (Saguinus_graellsi: 0.002565, Saguinus_nigricollis: 0.002565): 0.017120): 0.001760, Saguinus_tripartitus: 0.021445): 0.006793): 0.046145, (((Saguinus_oedipus: 0.007123, Saguinus_geoffroyi: 0.007123): 0.047080, ((Saguinus_bicolor: 0.005948, Saguinus_martinsi: 0.005948): 0.022691, (Saguinus_niger: 0.010832, Saguinus_midas: 0.010832): 0.017806): 0.025564): 0.008820, ((Saguinus_imperator: 0.033836, (Saguinus_labiatus: 0.019102, Saguinus_mystax: 0.019102): 0.014735): 0.010631, Saguinus_leucopus: 0.044467): 0.018555): 0.011361): 0.072253, (((Leontopithecus_rosalia: 0.001229, Leontopithecus_chrysopygus: 0.001229): 0.004698, Leontopithecus_chrysomelas: 0.005926): 0.133361, (Callimico_goeldii: 0.122087, (Callithrix_saterei: 0.071806, ((Callithrix_aurita: 0.024005, (Callithrix_geoffroyi: 0.011909, (Callithrix_kuhlii: 0.009823, (Callithrix_jacchus: 0.007401, Callithrix_penicillata: 0.007401): 0.002422): 0.002086): 0.012097): 0.026899, ((Callithrix_argentata: 0.022147, (Callithrix_emiliae: 0.011582, (Callithrix_mauesi: 0.005561, Callithrix_humeralifera: 0.005561): 0.006021): 0.010565): 0.017960, (Callithrix_humilis: 0.024761, Callithrix_pygmaea: 0.024761): 0.015346): 0.010797): 0.020902): 0.050281): 0.017200): 0.007349): 0.054989, ((Aotus_vociferans: 0.032635, ((Aotus_lemurinus: 0.019789, Aotus_griseimembra: 0.019789): 0.009665, Aotus_trivirgatus: 0.029454): 0.003181): 0.004722, (Aotus_nancymaae: 0.035945, (Aotus_nigriceps: 0.013060, (Aotus_infulatus: 0.004314, Aotus_azarae: 0.004314): 0.008746): 0.022885): 0.001411): 0.164269): 0.004012): 0.022515): 0.012159): 0.187684, ((((Pongo_abelii: 0.016911, Pongo_pygmaeus: 0.016911): 0.156277, (((Pan_troglodytes: 0.021921, Pan_paniscus: 0.021921): 0.054363, Homo_sapiens: 0.076284): 0.018424, (Gorilla_beringei: 0.021831, Gorilla_gorilla: 0.021831): 0.072877): 0.078479): 0.029005, ((Hoolock_hoolock: 0.026215, Hoolock_leuconedys: 0.026215): 0.051212, ((Symphalangus_syndactylus: 0.063339, (Hylobates_pileatus: 0.037134, (((Hylobates_klossii: 0.020284, Hylobates_moloch: 0.020284): 0.005183, ((Hylobates_agilis: 0.009644, Hylobates_albibarbis: 0.009644): 0.012577, (Hylobates_abbotti: 0.013504, (Hylobates_funereus: 0.010415, Hylobates_muelleri: 0.010415): 0.003089): 0.008716): 0.003247): 0.003002, Hylobates_lar: 0.028469): 0.008665): 0.026205): 0.006160, ((((Nomascus_leucogenys: 0.003218, Nomascus_siki: 0.003218): 0.006804, Nomascus_gabriellae: 0.010022): 0.004295, Nomascus_concolor: 0.014317): 0.015321, (Nomascus_nasutus: 0.020867, Nomascus_hainanus: 0.020867): 0.008772): 0.039861): 0.007928): 0.124765): 0.075070, ((((Macaca_sylvanus: 0.046727, ((((Macaca_nemestrina: 0.021440, Macaca_silenus: 0.021440): 0.000395, (Macaca_pagensis: 0.017233, (Macaca_siberu: 0.010531, Macaca_leonina: 0.010531): 0.006702): 0.004602): 0.008354, ((Macaca_hecki: 0.018772, (Macaca_nigrescens: 0.017776, (Macaca_tonkeana: 0.014559, Macaca_nigra: 0.014559): 0.003217): 0.000997): 0.000785, (Macaca_maura: 0.017091, (Macaca_ochreata: 0.008444, Macaca_brunnescens: 0.008444): 0.008647): 0.002466): 0.010631): 0.008426, ((Macaca_arctoides: 0.029671, (((Macaca_thibetana: 0.005585, Macaca_assamensis: 0.005585): 0.013789, Macaca_sinica: 0.019374): 0.004855, (Macaca_radiata: 0.010399, Macaca_munzala: 0.010399): 0.013830): 0.005441): 0.001978, ((Macaca_cyclopis: 0.019030, (Macaca_mulatta: 0.017611, Macaca_fuscata: 0.017611): 0.001419): 0.008589, Macaca_fascicularis: 0.027619): 0.004029): 0.006966): 0.008113): 0.032155, ((Theropithecus_gelada: 0.039900, (((Papio_papio: 0.012946, (Papio_hamadryas: 0.012198, ((Papio_cynocephalus: 0.007397, Papio_ursinus: 0.007397): 0.003762, Papio_anubis: 0.011159): 0.001039): 0.000748): 0.005196, Rungwecebus_kipunji: 0.018142): 0.014680, (Lophocebus_albigena: 0.002408, Lophocebus_aterrimus: 0.002408): 0.030414): 0.007077): 0.028336, ((Mandrillus_leucophaeus: 0.011763, Mandrillus_sphinx: 0.011763): 0.034756, ((Cercocebus_chrysogaster: 0.004381, (Cercocebus_galeritus: 0.002552, Cercocebus_agilis: 0.002552): 0.001829): 0.031123, (Cercocebus_atys: 0.010074, Cercocebus_torquatus: 0.010074): 0.025430): 0.011014): 0.021718): 0.010646): 0.020377, ((((Cercopithecus_campbelli: 0.049302, (Chlorocebus_cynosuros: 0.021500, (Chlorocebus_sabaeus: 0.020316, (Chlorocebus_pygerythrus: 0.011984, (Chlorocebus_aethiops: 0.009458, Chlorocebus_tantalus: 0.009458): 0.002525): 0.008332): 0.001185): 0.027802): 0.007631, ((Cercopithecus_solatus: 0.036489, (Cercopithecus_preussi: 0.012398, Cercopithecus_lhoesti: 0.012398): 0.024091): 0.015797, Erythrocebus_patas: 0.052286): 0.004647): 0.014201, (Cercopithecus_hamlyni: 0.062480, ((Cercopithecus_neglectus: 0.056258, (Cercopithecus_mona: 0.023077, (Cercopithecus_pogonias: 0.014482, Cercopithecus_wolfi: 0.014482): 0.008595): 0.033181): 0.002642, (Cercopithecus_diana: 0.054136, ((Cercopithecus_nictitans: 0.028582, (Cercopithecus_albogularis: 0.007624, Cercopithecus_mitis: 0.007624): 0.020958): 0.012001, ((Cercopithecus_ascanius: 0.020192, (Cercopithecus_erythrogaster: 0.015423, (Cercopithecus_erythrotis: 0.005460, Cercopithecus_cephus: 0.005460): 0.009963): 0.004768): 0.016565, Cercopithecus_petaurista: 0.036757): 0.003826): 0.013553): 0.004765): 0.003580): 0.008654): 0.005261, (Allenopithecus_nigroviridis: 0.072431, (Miopithecus_ogouensis: 0.036236, Miopithecus_talapoin: 0.036236): 0.036195): 0.003965): 0.022864): 0.056418, ((((((Semnopithecus_priam: 0.009962, Trachypithecus_vetulus: 0.009962): 0.016035, (((Semnopithecus_hector: 0.011494, Semnopithecus_entellus: 0.011494): 0.007888, Trachypithecus_johnii: 0.019382): 0.004329, (Trachypithecus_geei: 0.002014, Trachypithecus_pileatus: 0.002014): 0.021697): 0.002286): 0.031495, ((Trachypithecus_laotum: 0.014439, (Trachypithecus_hatinhensis: 0.006045, (Trachypithecus_delacouri: 0.005066, (Trachypithecus_francoisi: 0.003650, Trachypithecus_poliocephalus: 0.003650): 0.001416): 0.000980): 0.008394): 0.008795, ((Trachypithecus_auratus: 0.007873, (Trachypithecus_cristatus: 0.006743, Trachypithecus_germaini: 0.006743): 0.001130): 0.013208, ((Trachypithecus_barbei: 0.014133, Trachypithecus_obscurus: 0.014133): 0.005823, Trachypithecus_phayrei: 0.019956): 0.001124): 0.002154): 0.034258): 0.018561, (((Rhinopithecus_bieti: 0.025331, (Rhinopithecus_roxellana: 0.020932, Rhinopithecus_avunculus: 0.020932): 0.004398): 0.002440, Rhinopithecus_brelichi: 0.027771): 0.035008, ((Simias_concolor: 0.025114, Nasalis_larvatus: 0.025114): 0.033830, (Pygathrix_nigripes: 0.020333, (Pygathrix_cinerea: 0.003117, Pygathrix_nemaeus: 0.003117): 0.017216): 0.038611): 0.003835): 0.013274): 0.002503, (((Presbytis_siberu: 0.008407, Presbytis_potenziani: 0.008407): 0.006398, ((Presbytis_sumatranu: 0.009486, ((Presbytis_bicolor: 0.006944, Presbytis_rubicunda: 0.006944): 0.001197, Presbytis_melalophos: 0.008142): 0.001344): 0.001707, ((Presbytis_fredericae: 0.004944, Presbytis_comata: 0.004944): 0.004655, Presbytis_mitrata: 0.009599): 0.001595): 0.003613): 0.027192, ((Presbytis_hosei: 0.034764, (Presbytis_frontata: 0.029833, Presbytis_chrysomelas: 0.029833): 0.004931): 0.005544, (Presbytis_thomasi: 0.035999, Presbytis_femoralis: 0.035999): 0.004309): 0.001690): 0.036558): 0.023016, ((((((Piliocolobus_foai: 0.009034, (Piliocolobus_tephrosceles: 0.006764, Piliocolobus_rufomitratus: 0.006764): 0.002271): 0.007216, (Piliocolobus_kirkii: 0.007881, Piliocolobus_gordonorum: 0.007881): 0.008369): 0.016488, ((Piliocolobus_pennantii: 0.004189, Piliocolobus_preussi: 0.004189): 0.021888, Piliocolobus_tholloni: 0.026078): 0.006660): 0.003769, Piliocolobus_badius: 0.036507): 0.034632, Procolobus_verus: 0.071139): 0.008387, (Colobus_satanas: 0.043501, (((Colobus_polykomos: 0.001692, Colobus_vellerosus: 0.001692): 0.015202, Colobus_guereza: 0.016894): 0.009281, Colobus_angolensis: 0.026175): 0.017326): 0.036025): 0.022046): 0.054105): 0.121586): 0.150733): 0.158028): 0.041638): 0.033900): 0.011954, (Lagomorpha: 0.649810, (Tupaia_glis: 0.076343, Tupaia_minor: 0.076343): 0.573468): 0.023705);

2. Newick timetree with autocorrelated rates and soft-bounded constraints. One unit = 100 million years.

(((Galeopterus_variegatus: 0.086546, Cynocephalus_volans: 0.086546): 0.617496, (((((Galago_thomasi: 0.154339, (Galago_demidoff: 0.148420, ((Galago_matschiei: 0.040856, (Galago_moholi: 0.007868, (Galago_gallarum: 0.006480, Galago_senegalensis: 0.006480): 0.001388): 0.032988): 0.088232, ((Galago_orinus: 0.076728, (Galago_granti: 0.019514, Galago_zanzibaricus: 0.019514): 0.057214): 0.039096, ((Otolemur_monteiri: 0.079856, (Otolemur_crassicaudatus: 0.032069, Otolemur_garnettii: 0.032069): 0.047787): 0.003309, (Galago_gabonensis: 0.006288, Galago_alleni: 0.006288): 0.076877): 0.032659): 0.013264): 0.019332): 0.005918): 0.048937, Euoticus_elegantulus: 0.203275): 0.111140, (((Loris_lydekkerianus: 0.006073, Loris_tardigradus: 0.006073): 0.193427, (Nycticebus_pygmaeus: 0.049135, (Nycticebus_menagensis: 0.031167, (Nycticebus_javanicus: 0.004511, (Nycticebus_bengalensis: 0.002964, Nycticebus_coucang: 0.002964): 0.001548): 0.026656): 0.017968): 0.150365): 0.090379, (Perodicticus_potto: 0.216022, (Arctocebus_calabarensis: 0.011517, Arctocebus_aureus: 0.011517): 0.204505): 0.073857): 0.024536): 0.219562, (Daubentonia_madagascariensis: 0.503395, (((Varecia_rubra: 0.007325, Varecia_variegata: 0.007325): 0.208977, ((Lemur_catta: 0.086925, (Prolemur_simus: 0.079744, (Hapalemur_aureus: 0.053658, (Hapalemur_occidentalis: 0.007386, (Hapalemur_alaotrensis: 0.003078, (Hapalemur_griseus: 0.002061, Hapalemur_meridionalis: 0.002061): 0.001018): 0.004308): 0.046272): 0.026086): 0.007180): 0.098998, (((((Eulemur_rufifrons: 0.019289, (Eulemur_fulvus: 0.012770, (Eulemur_albifrons: 0.010898, Eulemur_sanfordi: 0.010898): 0.001872): 0.006519): 0.007049, ((Eulemur_cinereiceps: 0.011120, Eulemur_collaris: 0.011120): 0.013142, Eulemur_rufus: 0.024261): 0.002077): 0.025928, Eulemur_mongoz: 0.052266): 0.008378, ((Eulemur_macaco: 0.010257, Eulemur_flavifrons: 0.010257): 0.043584, Eulemur_coronatus: 0.053841): 0.006803): 0.008715, Eulemur_rubriventer: 0.069359): 0.116564): 0.030379): 0.095159, (((Phaner_pallescens: 0.202671, ((((Lepilemur_aeeclis: 0.028565, (Lepilemur_randrianasoloi: 0.020944, (Lepilemur_hubbardorum: 0.013481, Lepilemur_ruficaudatus: 0.013481): 0.007463): 0.007621): 0.014898, (Lepilemur_leucopus: 0.007528, Lepilemur_petteri: 0.007528): 0.035936): 0.013558, ((Lepilemur_microdon: 0.031756, (Lepilemur_otto: 0.011809, (Lepilemur_grewcockorum: 0.009166, Lepilemur_edwardsi: 0.009166): 0.002643): 0.019947): 0.011377, (Lepilemur_septentrionalis: 0.024971, ((Lepilemur_ankaranensis: 0.008157, (Lepilemur_tymerlachsoni: 0.007397, Lepilemur_milanoii: 0.007397): 0.000760): 0.008167, ((Lepilemur_mittermeieri: 0.012909, (Lepilemur_sahamalazensis: 0.007829, Lepilemur_ahmansoni: 0.007829): 0.005080): 0.002272, Lepilemur_dorsalis: 0.015181): 0.001143): 0.008648): 0.018161): 0.013888): 0.027004, (Lepilemur_seali: 0.055016, (Lepilemur_wrighti: 0.034397, (Lepilemur_fleuretae: 0.023844, (Lepilemur_mustelinus: 0.012914, (Lepilemur_betsileo: 0.009008, Lepilemur_jamesorum: 0.009008): 0.003906): 0.010930): 0.010553): 0.020619): 0.029009): 0.118646): 0.068397, ((Allocebus_trichotis: 0.174581, ((Mirza_coquereli: 0.006572, Mirza_zaza: 0.006572): 0.131429, ((Microcebus_griseorufus: 0.046392, Microcebus_murinus: 0.046392): 0.024071, (((Microcebus_macarthurii: 0.025551, Microcebus_sp_nova3Radespiel08: 0.025551): 0.014972, (Microcebus_danfossi: 0.030811, (Microcebus_ravelobensis: 0.007257, Microcebus_bongolavensis: 0.007257): 0.023553): 0.009713): 0.016339, ((((Microcebus_rufus: 0.031962, ((Microcebus_sambiranensis: 0.011345, Microcebus_arnholdi: 0.011345): 0.015126, (Microcebus_mamiratra: 0.012605, Microcebus_margotmarshae: 0.012605): 0.013867): 0.005491): 0.005266, (((Microcebus_myoxinus: 0.015712, Microcebus_berthae: 0.015712): 0.012488, Microcebus_lehilahytsara: 0.028200): 0.006726, Microcebus_mittermeieri: 0.034926): 0.002302): 0.004992, (Microcebus_simmonsi: 0.037302, (((Microcebus_jollyae: 0.000825, (Microcebus_sp_nova1Louis06: 0.000521, Microcebus_sp_nova3Louis06: 0.000521): 0.000304): 0.027806, (Microcebus_gerpi: 0.010785, Microcebus_spDWW2010aMarolambo: 0.010785): 0.017845): 0.004508, Microcebus_spDWW2010aManantantel: 0.033139): 0.004163): 0.004918): 0.005394, Microcebus_tavaratra: 0.047614): 0.009248): 0.013600): 0.067538): 0.036580): 0.047289, (Cheirogaleus_sibreei: 0.097325, (Cheirogaleus_crossleyi: 0.078906, (Cheirogaleus_major: 0.052977, Cheirogaleus_medius: 0.052977): 0.025929): 0.018419): 0.124544): 0.049200): 0.030872, (Indri_indri: 0.198412, (((((Avahi_ramanantsoavani: 0.012425, Avahi_meridionalis: 0.012425): 0.007159, (Avahi_peyrierasi: 0.007305, Avahi_betsilio: 0.007305): 0.012279): 0.012823, Avahi_laniger: 0.032407): 0.043628, (Avahi_occidentalis: 0.018713, (Avahi_cleesei: 0.016419, Avahi_unicolor: 0.016419): 0.002295): 0.057321): 0.083868, ((Propithecus_perrieri: 0.031372, (Propithecus_edwardsi: 0.016611, Propithecus_diadema: 0.016611): 0.014761): 0.034752, ((Propithecus_tattersalli: 0.017534, Propithecus_coquereli: 0.017534): 0.015246, Propithecus_verreauxi: 0.032780): 0.033343): 0.093779): 0.038510): 0.103529): 0.009519): 0.191935): 0.030583): 0.133088, (((Tarsius_dentatus: 0.078837, ((Tarsius_wallacei: 0.024721, Tarsius_tarsier: 0.024721): 0.024282, (Tarsius_sangirensis: 0.036965, Tarsius_lariang: 0.036965): 0.012038): 0.029834): 0.151317, (Tarsius_bancanus: 0.064227, Tarsius_syrichta: 0.064227): 0.165926): 0.402771, ((((((Cacajao_calvus: 0.034244, (Cacajao_melanocephalus: 0.011078, (Cacajao_hosomi: 0.002283, Cacajao_ayresi: 0.002283): 0.008795): 0.023166): 0.036340, ((Chiropotes_israelita: 0.012026, Chiropotes_chiropotes: 0.012026): 0.020812, Chiropotes_utahicki: 0.032838): 0.037746): 0.072680, (Pithecia_pithecia: 0.039559, (Pithecia_monachus: 0.005142, Pithecia_irrorata: 0.005142): 0.034417): 0.103705): 0.065520, ((Callicebus_lugens: 0.033298, Callicebus_torquatus: 0.033298): 0.045773, (((Callicebus_personatus: 0.020001, Callicebus_coimbrai: 0.020001): 0.016027, Callicebus_nigrifrons: 0.036028): 0.035404, ((Callicebus_cupreus: 0.022429, (Callicebus_brunneus: 0.020667, (Callicebus_moloch: 0.008781, Callicebus_hoffmannsi: 0.008781): 0.011887): 0.001762): 0.009886, (Callicebus_caligatus: 0.005456, Callicebus_donacophilus: 0.005456): 0.026859): 0.039118): 0.007638): 0.129714): 0.034670, ((((Alouatta_pigra: 0.024923, (Alouatta_palliata: 0.003571, Alouatta_coibensis: 0.003571): 0.021352): 0.016294, ((Alouatta_caraya: 0.033751, (Alouatta_sara: 0.030483, (Alouatta_nigerrima: 0.005490, (Alouatta_seniculus: 0.000360, Alouatta_macconnelli: 0.000360): 0.005131): 0.024993): 0.003268): 0.003192, (Alouatta_guariba: 0.027307, Alouatta_belzebul: 0.027307): 0.009636): 0.004274): 0.131710, (((Brachyteles_hypoxanthus: 0.032138, Brachyteles_arachnoides: 0.032138): 0.083526, ((Lagothrix_cana: 0.005488, (Lagothrix_lagotricha: 0.003977, Lagothrix_poeppigii: 0.003977): 0.001511): 0.003834, Lagothrix_lugens: 0.009322): 0.106342): 0.017298, ((Ateles_fusciceps: 0.024635, (Ateles_hybridus: 0.023930, (Ateles_belzebuth: 0.017253, Ateles_geoffroyi: 0.017253): 0.006678): 0.000705): 0.009943, (Ateles_paniscus: 0.013557, (Ateles_marginatus: 0.005442, Ateles_chamek: 0.005442): 0.008115): 0.021022): 0.098383): 0.039965): 0.057834, ((((Saimiri_ustus: 0.012710, (Saimiri_sciureus: 0.007871, Saimiri_oerstedii: 0.007871): 0.004838): 0.006690, Saimiri_boliviensis: 0.019400): 0.161302, (((Cebus_libidinosus: 0.003426, Cebus_apella: 0.003426): 0.016351, (Cebus_xanthosternos: 0.017097, Cebus_robustus: 0.017097): 0.002680): 0.046311, (Cebus_capucinus: 0.018087, ((Cebus_kaapori: 0.005318, Cebus_olivaceus: 0.005318): 0.011251, Cebus_albifrons: 0.016569): 0.001518): 0.048001): 0.114614): 0.026313, ((((Saguinus_fuscicollis: 0.028807, ((Saguinus_melanoleucus: 0.020120, (Saguinus_graellsi: 0.002356, Saguinus_nigricollis: 0.002356): 0.017764): 0.001719, Saguinus_tripartitus: 0.021839): 0.006969): 0.044242, (((Saguinus_oedipus: 0.006919, Saguinus_geoffroyi: 0.006919): 0.045918, ((Saguinus_bicolor: 0.005418, Saguinus_martinsi: 0.005418): 0.021474, (Saguinus_niger: 0.008928, Saguinus_midas: 0.008928): 0.017964): 0.025944): 0.008981, ((Saguinus_imperator: 0.032868, (Saguinus_labiatus: 0.018209, Saguinus_mystax: 0.018209): 0.014659): 0.009894, Saguinus_leucopus: 0.042762): 0.019056): 0.011231): 0.072915, (((Leontopithecus_rosalia: 0.001508, Leontopithecus_chrysopygus: 0.001508): 0.004501, Leontopithecus_chrysomelas: 0.006009): 0.132533, (Callimico_goeldii: 0.120895, (Callithrix_saterei: 0.073056, ((Callithrix_aurita: 0.023418, (Callithrix_geoffroyi: 0.011703, (Callithrix_kuhlii: 0.009681, (Callithrix_jacchus: 0.007358, Callithrix_penicillata: 0.007358): 0.002323): 0.002022): 0.011714): 0.026723, ((Callithrix_argentata: 0.022066, (Callithrix_emiliae: 0.011926, (Callithrix_mauesi: 0.005698, Callithrix_humeralifera: 0.005698): 0.006228): 0.010140): 0.017481, (Callithrix_humilis: 0.023808, Callithrix_pygmaea: 0.023808): 0.015740): 0.010593): 0.022916): 0.047839): 0.017646): 0.007422): 0.056972, ((Aotus_vociferans: 0.031846, ((Aotus_lemurinus: 0.019194, Aotus_griseimembra: 0.019194): 0.009397, Aotus_trivirgatus: 0.028591): 0.003255): 0.004763, (Aotus_nancymaae: 0.035135, (Aotus_nigriceps: 0.012916, (Aotus_infulatus: 0.004106, Aotus_azarae: 0.004106): 0.008810): 0.022219): 0.001475): 0.166326): 0.004079): 0.023747): 0.012693): 0.201167, ((((Pongo_abelii: 0.016631, Pongo_pygmaeus: 0.016631): 0.163137, (((Pan_troglodytes: 0.021095, Pan_paniscus: 0.021095): 0.058193, Homo_sapiens: 0.079289): 0.018233, (Gorilla_beringei: 0.021659, Gorilla_gorilla: 0.021659): 0.075863): 0.082246): 0.029478, ((Hoolock_hoolock: 0.025589, Hoolock_leuconedys: 0.025589): 0.052236, ((Symphalangus_syndactylus: 0.063068, (Hylobates_pileatus: 0.036369, (((Hylobates_klossii: 0.019798, Hylobates_moloch: 0.019798): 0.005138, ((Hylobates_agilis: 0.009564, Hylobates_albibarbis: 0.009564): 0.012079, (Hylobates_abbotti: 0.013515, (Hylobates_funereus: 0.009753, Hylobates_muelleri: 0.009753): 0.003763): 0.008128): 0.003292): 0.002927, Hylobates_lar: 0.027863): 0.008506): 0.026699): 0.006534, ((((Nomascus_leucogenys: 0.003299, Nomascus_siki: 0.003299): 0.006662, Nomascus_gabriellae: 0.009961): 0.004429, Nomascus_concolor: 0.014391): 0.015805, (Nomascus_nasutus: 0.020956, Nomascus_hainanus: 0.020956): 0.009239): 0.039407): 0.008224): 0.131420): 0.078541, ((((Macaca_sylvanus: 0.045198, ((((Macaca_nemestrina: 0.020488, Macaca_silenus: 0.020488): 0.000395, (Macaca_pagensis: 0.016366, (Macaca_siberu: 0.009621, Macaca_leonina: 0.009621): 0.006745): 0.004517): 0.008194, ((Macaca_hecki: 0.018042, (Macaca_nigrescens: 0.017054, (Macaca_tonkeana: 0.014031, Macaca_nigra: 0.014031): 0.003023): 0.000988): 0.000734, (Macaca_maura: 0.016377, (Macaca_ochreata: 0.007841, Macaca_brunnescens: 0.007841): 0.008536): 0.002399): 0.010301): 0.008204, ((Macaca_arctoides: 0.028632, (((Macaca_thibetana: 0.005317, Macaca_assamensis: 0.005317): 0.013205, Macaca_sinica: 0.018522): 0.004666, (Macaca_radiata: 0.009921, Macaca_munzala: 0.009921): 0.013267): 0.005445): 0.001957, ((Macaca_cyclopis: 0.018575, (Macaca_mulatta: 0.017168, Macaca_fuscata: 0.017168): 0.001407): 0.008126, Macaca_fascicularis: 0.026702): 0.003887): 0.006692): 0.007917): 0.032226, ((Theropithecus_gelada: 0.039389, (((Papio_papio: 0.012580, (Papio_hamadryas: 0.011817, ((Papio_cynocephalus: 0.007104, Papio_ursinus: 0.007104): 0.003738, Papio_anubis: 0.010842): 0.000975): 0.000763): 0.005031, Rungwecebus_kipunji: 0.017611): 0.014686, (Lophocebus_albigena: 0.003052, Lophocebus_aterrimus: 0.003052): 0.029244): 0.007092): 0.027134, ((Mandrillus_leucophaeus: 0.011057, Mandrillus_sphinx: 0.011057): 0.035117, ((Cercocebus_chrysogaster: 0.004149, (Cercocebus_galeritus: 0.002304, Cercocebus_agilis: 0.002304): 0.001845): 0.030894, (Cercocebus_atys: 0.010414, Cercocebus_torquatus: 0.010414): 0.024628): 0.011131): 0.020349): 0.010901): 0.020626, ((((Cercopithecus_campbelli: 0.047292, (Chlorocebus_cynosuros: 0.020576, (Chlorocebus_sabaeus: 0.019519, (Chlorocebus_pygerythrus: 0.011610, (Chlorocebus_aethiops: 0.009094, Chlorocebus_tantalus: 0.009094): 0.002517): 0.007909): 0.001056): 0.026717): 0.008248, ((Cercopithecus_solatus: 0.035917, (Cercopithecus_preussi: 0.012368, Cercopithecus_lhoesti: 0.012368): 0.023549): 0.014980, Erythrocebus_patas: 0.050897): 0.004644): 0.014505, (Cercopithecus_hamlyni: 0.061547, ((Cercopithecus_neglectus: 0.055533, (Cercopithecus_mona: 0.023385, (Cercopithecus_pogonias: 0.016183, Cercopithecus_wolfi: 0.016183): 0.007202): 0.032148): 0.002471, (Cercopithecus_diana: 0.053233, ((Cercopithecus_nictitans: 0.028277, (Cercopithecus_albogularis: 0.007436, Cercopithecus_mitis: 0.007436): 0.020841): 0.011413, ((Cercopithecus_ascanius: 0.019537, (Cercopithecus_erythrogaster: 0.015070, (Cercopithecus_erythrotis: 0.005607, Cercopithecus_cephus: 0.005607): 0.009463): 0.004467): 0.016562, Cercopithecus_petaurista: 0.036099): 0.003592): 0.013542): 0.004772): 0.003542): 0.008499): 0.005470, (Allenopithecus_nigroviridis: 0.072109, (Miopithecus_ogouensis: 0.035120, Miopithecus_talapoin: 0.035120): 0.036990): 0.003406): 0.022535): 0.058825, ((((((Semnopithecus_priam: 0.009542, Trachypithecus_vetulus: 0.009542): 0.016224, (((Semnopithecus_hector: 0.011178, Semnopithecus_entellus: 0.011178): 0.007916, Trachypithecus_johnii: 0.019094): 0.004342, (Trachypithecus_geei: 0.002369, Trachypithecus_pileatus: 0.002369): 0.021067): 0.002330): 0.029635, ((Trachypithecus_laotum: 0.014252, (Trachypithecus_hatinhensis: 0.006139, (Trachypithecus_delacouri: 0.005145, (Trachypithecus_francoisi: 0.003622, Trachypithecus_poliocephalus: 0.003622): 0.001523): 0.000994): 0.008113): 0.008336, ((Trachypithecus_auratus: 0.007755, (Trachypithecus_cristatus: 0.006532, Trachypithecus_germaini: 0.006532): 0.001223): 0.012608, ((Trachypithecus_barbei: 0.013535, Trachypithecus_obscurus: 0.013535): 0.005756, Trachypithecus_phayrei: 0.019291): 0.001072): 0.002224): 0.032814): 0.021335, (((Rhinopithecus_bieti: 0.025475, (Rhinopithecus_roxellana: 0.020909, Rhinopithecus_avunculus: 0.020909): 0.004566): 0.002373, Rhinopithecus_brelichi: 0.027848): 0.035185, ((Simias_concolor: 0.024830, Nasalis_larvatus: 0.024830): 0.034401, (Pygathrix_nigripes: 0.019457, (Pygathrix_cinerea: 0.003199, Pygathrix_nemaeus: 0.003199): 0.016258): 0.039775): 0.003802): 0.013702): 0.002480, (((Presbytis_siberu: 0.008800, Presbytis_potenziani: 0.008800): 0.005948, ((Presbytis_sumatranu: 0.009442, ((Presbytis_bicolor: 0.006957, Presbytis_rubicunda: 0.006957): 0.001184, Presbytis_melalophos: 0.008140): 0.001302): 0.001793, ((Presbytis_fredericae: 0.005167, Presbytis_comata: 0.005167): 0.004395, Presbytis_mitrata: 0.009562): 0.001674): 0.003513): 0.027029, ((Presbytis_hosei: 0.034164, (Presbytis_frontata: 0.029045, Presbytis_chrysomelas: 0.029045): 0.005119): 0.005845, (Presbytis_thomasi: 0.035390, Presbytis_femoralis: 0.035390): 0.004619): 0.001769): 0.037438): 0.024013, ((((((Piliocolobus_foai: 0.009027, (Piliocolobus_tephrosceles: 0.006624, Piliocolobus_rufomitratus: 0.006624): 0.002403): 0.007068, (Piliocolobus_kirkii: 0.007878, Piliocolobus_gordonorum: 0.007878): 0.008216): 0.016613, ((Piliocolobus_pennantii: 0.004421, Piliocolobus_preussi: 0.004421): 0.021379, Piliocolobus_tholloni: 0.025800): 0.006908): 0.003619, Piliocolobus_badius: 0.036326): 0.035580, Procolobus_verus: 0.071907): 0.008279, (Colobus_satanas: 0.044513, (((Colobus_polykomos: 0.001766, Colobus_vellerosus: 0.001766): 0.015828, Colobus_guereza: 0.017594): 0.008955, Colobus_angolensis: 0.026549): 0.017964): 0.035672): 0.023043): 0.053647): 0.130913): 0.156833): 0.188303): 0.034142): 0.036976): 0.013219, (Lagomorpha: 0.691594, (Tupaia_glis: 0.076612, Tupaia_minor: 0.076612): 0.614982): 0.025666);

3. Newick timetree with independent rates and hard-bounded constraints. One unit = 100 million years.

(((Galeopterus_variegatus: 0.129417, Cynocephalus_volans: 0.129417): 0.673013, (((((Galago_thomasi: 0.139250, (Galago_demidoff: 0.136829, ((Galago_matschiei: 0.027217, (Galago_moholi: 0.008623, (Galago_gallarum: 0.007282, Galago_senegalensis: 0.007282): 0.001341): 0.018594): 0.083736, ((Galago_orinus: 0.043439, (Galago_granti: 0.014145, Galago_zanzibaricus: 0.014145): 0.029294): 0.042599, ((Otolemur_monteiri: 0.051118, (Otolemur_crassicaudatus: 0.028984, Otolemur_garnettii: 0.028984): 0.022134): 0.009673, (Galago_gabonensis: 0.003586, Galago_alleni: 0.003586): 0.057205): 0.025246): 0.024915): 0.025876): 0.002421): 0.109147, Euoticus_elegantulus: 0.248397): 0.125834, (((Loris_lydekkerianus: 0.003083, Loris_tardigradus: 0.003083): 0.184034, (Nycticebus_pygmaeus: 0.053344, (Nycticebus_menagensis: 0.024517, (Nycticebus_javanicus: 0.009782, (Nycticebus_bengalensis: 0.002935, Nycticebus_coucang: 0.002935): 0.006847): 0.014735): 0.028826): 0.133773): 0.162382, (Perodicticus_potto: 0.200711, (Arctocebus_calabarensis: 0.006296, Arctocebus_aureus: 0.006296): 0.194415): 0.148788): 0.024733): 0.178989, (Daubentonia_madagascariensis: 0.497975, (((Varecia_rubra: 0.006432, Varecia_variegata: 0.006432): 0.194406, ((Lemur_catta: 0.062856, (Prolemur_simus: 0.053527, (Hapalemur_aureus: 0.030072, (Hapalemur_occidentalis: 0.007694, (Hapalemur_alaotrensis: 0.003638, (Hapalemur_griseus: 0.001589, Hapalemur_meridionalis: 0.001589): 0.002049): 0.004056): 0.022378): 0.023455): 0.009329): 0.110836, (((((Eulemur_rufifrons: 0.013081, (Eulemur_fulvus: 0.008901, (Eulemur_albifrons: 0.006316, Eulemur_sanfordi: 0.006316): 0.002585): 0.004180): 0.004527, ((Eulemur_cinereiceps: 0.006180, Eulemur_collaris: 0.006180): 0.008925, Eulemur_rufus: 0.015104): 0.002503): 0.014557, Eulemur_mongoz: 0.032165): 0.005184, ((Eulemur_macaco: 0.006416, Eulemur_flavifrons: 0.006416): 0.026909, Eulemur_coronatus: 0.033325): 0.004023): 0.017098, Eulemur_rubriventer: 0.054447): 0.119245): 0.027146): 0.125634, (((Phaner_pallescens: 0.226892, ((((Lepilemur_aeeclis: 0.028625, (Lepilemur_randrianasoloi: 0.021684, (Lepilemur_hubbardorum: 0.012007, Lepilemur_ruficaudatus: 0.012007): 0.009677): 0.006940): 0.015620, (Lepilemur_leucopus: 0.008400, Lepilemur_petteri: 0.008400): 0.035845): 0.011578, ((Lepilemur_microdon: 0.024536, (Lepilemur_otto: 0.011815, (Lepilemur_grewcockorum: 0.008249, Lepilemur_edwardsi: 0.008249): 0.003566): 0.012721): 0.012147, (Lepilemur_septentrionalis: 0.020784, ((Lepilemur_ankaranensis: 0.009773, (Lepilemur_tymerlachsoni: 0.008116, Lepilemur_milanoii: 0.008116): 0.001657): 0.007188, ((Lepilemur_mittermeieri: 0.012395, (Lepilemur_sahamalazensis: 0.007830, Lepilemur_ahmansoni: 0.007830): 0.004566): 0.003363, Lepilemur_dorsalis: 0.015758): 0.001203): 0.003823): 0.015899): 0.019140): 0.026149, (Lepilemur_seali: 0.047237, (Lepilemur_wrighti: 0.030786, (Lepilemur_fleuretae: 0.018578, (Lepilemur_mustelinus: 0.011191, (Lepilemur_betsileo: 0.006960, Lepilemur_jamesorum: 0.006960): 0.004231): 0.007386): 0.012208): 0.016452): 0.034735): 0.144919): 0.054624, ((Allocebus_trichotis: 0.148708, ((Mirza_coquereli: 0.002930, Mirza_zaza: 0.002930): 0.123629, ((Microcebus_griseorufus: 0.035030, Microcebus_murinus: 0.035030): 0.033398, (((Microcebus_macarthurii: 0.020206, Microcebus_sp_nova3Radespiel08: 0.020206): 0.017779, (Microcebus_danfossi: 0.025641, (Microcebus_ravelobensis: 0.008251, Microcebus_bongolavensis: 0.008251): 0.017390): 0.012344): 0.018047, ((((Microcebus_rufus: 0.029605, ((Microcebus_sambiranensis: 0.011428, Microcebus_arnholdi: 0.011428): 0.012716, (Microcebus_mamiratra: 0.010231, Microcebus_margotmarshae: 0.010231): 0.013912): 0.005462): 0.003517, (((Microcebus_myoxinus: 0.011538, Microcebus_berthae: 0.011538): 0.012079, Microcebus_lehilahytsara: 0.023617): 0.006991, Microcebus_mittermeieri: 0.030608): 0.002514): 0.003654, (Microcebus_simmonsi: 0.032564, (((Microcebus_jollyae: 0.001880, (Microcebus_sp_nova1Louis06: 0.001541, Microcebus_sp_nova3Louis06: 0.001541): 0.000339): 0.020120, (Microcebus_gerpi: 0.009160, Microcebus_spDWW2010aMarolambo: 0.009160): 0.012840): 0.005199, Microcebus_spDWW2010aManantantel: 0.027199): 0.005366): 0.004211): 0.010215, Microcebus_tavaratra: 0.046991): 0.009041): 0.012396): 0.058131): 0.022148): 0.066782, (Cheirogaleus_sibreei: 0.084222, (Cheirogaleus_crossleyi: 0.062902, (Cheirogaleus_major: 0.050293, Cheirogaleus_medius: 0.050293): 0.012610): 0.021319): 0.131268): 0.066025): 0.032105, (Indri_indri: 0.171394, (((((Avahi_ramanantsoavani: 0.007104, Avahi_meridionalis: 0.007104): 0.006763, (Avahi_peyrierasi: 0.004105, Avahi_betsilio: 0.004105): 0.009762): 0.008395, Avahi_laniger: 0.022262): 0.031525, (Avahi_occidentalis: 0.014390, (Avahi_cleesei: 0.011838, Avahi_unicolor: 0.011838): 0.002553): 0.039397): 0.083581, ((Propithecus_perrieri: 0.022894, (Propithecus_edwardsi: 0.016006, Propithecus_diadema: 0.016006): 0.006887): 0.031506, ((Propithecus_tattersalli: 0.016115, Propithecus_coquereli: 0.016115): 0.006179, Propithecus_verreauxi: 0.022294): 0.032106): 0.082968): 0.034026): 0.142226): 0.012852): 0.171503): 0.055245): 0.151792, (((Tarsius_dentatus: 0.029282, ((Tarsius_wallacei: 0.014124, Tarsius_tarsier: 0.014124): 0.009254, (Tarsius_sangirensis: 0.017181, Tarsius_lariang: 0.017181): 0.006198): 0.005903): 0.105294, (Tarsius_bancanus: 0.076875, Tarsius_syrichta: 0.076875): 0.057700): 0.450813, ((((((Cacajao_calvus: 0.025305, (Cacajao_melanocephalus: 0.011561, (Cacajao_hosomi: 0.001858, Cacajao_ayresi: 0.001858): 0.009703): 0.013744): 0.035148, ((Chiropotes_israelita: 0.011754, Chiropotes_chiropotes: 0.011754): 0.010344, Chiropotes_utahicki: 0.022098): 0.038354): 0.066667, (Pithecia_pithecia: 0.031114, (Pithecia_monachus: 0.004102, Pithecia_irrorata: 0.004102): 0.027012): 0.096006): 0.069636, ((Callicebus_lugens: 0.017884, Callicebus_torquatus: 0.017884): 0.058065, (((Callicebus_personatus: 0.020154, Callicebus_coimbrai: 0.020154): 0.012553, Callicebus_nigrifrons: 0.032707): 0.032452, ((Callicebus_cupreus: 0.021124, (Callicebus_brunneus: 0.019015, (Callicebus_moloch: 0.014233, Callicebus_hoffmannsi: 0.014233): 0.004782): 0.002109): 0.009705, (Callicebus_caligatus: 0.006119, Callicebus_donacophilus: 0.006119): 0.024710): 0.034330): 0.010789): 0.120807): 0.036764, ((((Alouatta_pigra: 0.018155, (Alouatta_palliata: 0.004977, Alouatta_coibensis: 0.004977): 0.013179): 0.015574, ((Alouatta_caraya: 0.026342, (Alouatta_sara: 0.021007, (Alouatta_nigerrima: 0.004534, (Alouatta_seniculus: 0.000320, Alouatta_macconnelli: 0.000320): 0.004213): 0.016474): 0.005335): 0.002798, (Alouatta_guariba: 0.021993, Alouatta_belzebul: 0.021993): 0.007147): 0.004589): 0.102718, (((Brachyteles_hypoxanthus: 0.022156, Brachyteles_arachnoides: 0.022156): 0.062761, ((Lagothrix_cana: 0.004168, (Lagothrix_lagotricha: 0.003036, Lagothrix_poeppigii: 0.003036): 0.001132): 0.007657, Lagothrix_lugens: 0.011825): 0.073093): 0.011730, ((Ateles_fusciceps: 0.013973, (Ateles_hybridus: 0.013570, (Ateles_belzebuth: 0.007856, Ateles_geoffroyi: 0.007856): 0.005714): 0.000404): 0.008635, (Ateles_paniscus: 0.007694, (Ateles_marginatus: 0.003649, Ateles_chamek: 0.003649): 0.004045): 0.014914): 0.074040): 0.039799): 0.083822, ((((Saimiri_ustus: 0.008999, (Saimiri_sciureus: 0.005415, Saimiri_oerstedii: 0.005415): 0.003584): 0.007277, Saimiri_boliviensis: 0.016276): 0.154857, (((Cebus_libidinosus: 0.002980, Cebus_apella: 0.002980): 0.011361, (Cebus_xanthosternos: 0.011830, Cebus_robustus: 0.011830): 0.002510): 0.035889, (Cebus_capucinus: 0.014664, ((Cebus_kaapori: 0.005112, Cebus_olivaceus: 0.005112): 0.008374, Cebus_albifrons: 0.013486): 0.001179): 0.035565): 0.120904): 0.024675, ((((Saguinus_fuscicollis: 0.028266, ((Saguinus_melanoleucus: 0.017126, (Saguinus_graellsi: 0.002722, Saguinus_nigricollis: 0.002722): 0.014404): 0.003687, Saguinus_tripartitus: 0.020813): 0.007453): 0.034078, (((Saguinus_oedipus: 0.005505, Saguinus_geoffroyi: 0.005505): 0.036490, ((Saguinus_bicolor: 0.008580, Saguinus_martinsi: 0.008580): 0.015032, (Saguinus_niger: 0.012637, Saguinus_midas: 0.012637): 0.010975): 0.018382): 0.011482, ((Saguinus_imperator: 0.027658, (Saguinus_labiatus: 0.014956, Saguinus_mystax: 0.014956): 0.012702): 0.007629, Saguinus_leucopus: 0.035287): 0.018190): 0.008867): 0.074933, (((Leontopithecus_rosalia: 0.001620, Leontopithecus_chrysopygus: 0.001620): 0.002988, Leontopithecus_chrysomelas: 0.004607): 0.125990, (Callimico_goeldii: 0.109241, (Callithrix_saterei: 0.061549, ((Callithrix_aurita: 0.027751, (Callithrix_geoffroyi: 0.008673, (Callithrix_kuhlii: 0.007927, (Callithrix_jacchus: 0.004638, Callithrix_penicillata: 0.004638): 0.003288): 0.000746): 0.019079): 0.016431, ((Callithrix_argentata: 0.016208, (Callithrix_emiliae: 0.009062, (Callithrix_mauesi: 0.005544, Callithrix_humeralifera: 0.005544): 0.003519): 0.007146): 0.019812, (Callithrix_humilis: 0.019170, Callithrix_pygmaea: 0.019170): 0.016850): 0.008162): 0.017367): 0.047692): 0.021356): 0.006681): 0.054528, ((Aotus_vociferans: 0.025033, ((Aotus_lemurinus: 0.012425, Aotus_griseimembra: 0.012425): 0.008858, Aotus_trivirgatus: 0.021282): 0.003751): 0.006569, (Aotus_nancymaae: 0.026698, (Aotus_nigriceps: 0.012813, (Aotus_infulatus: 0.004128, Aotus_azarae: 0.004128): 0.008684): 0.013885): 0.004904): 0.160203): 0.004003): 0.024461): 0.013251): 0.143549, ((((Pongo_abelii: 0.009394, Pongo_pygmaeus: 0.009394): 0.120329, (((Pan_troglodytes: 0.011351, Pan_paniscus: 0.011351): 0.045192, Homo_sapiens: 0.056544): 0.009503, (Gorilla_beringei: 0.014924, Gorilla_gorilla: 0.014924): 0.051123): 0.063676): 0.017073, ((Hoolock_hoolock: 0.014961, Hoolock_leuconedys: 0.014961): 0.045301, ((Symphalangus_syndactylus: 0.043846, (Hylobates_pileatus: 0.024996, (((Hylobates_klossii: 0.013594, Hylobates_moloch: 0.013594): 0.003339, ((Hylobates_agilis: 0.006982, Hylobates_albibarbis: 0.006982): 0.007229, (Hylobates_abbotti: 0.009081, (Hylobates_funereus: 0.006435, Hylobates_muelleri: 0.006435): 0.002646): 0.005130): 0.002721): 0.002337, Hylobates_lar: 0.019270): 0.005726): 0.018850): 0.004017, ((((Nomascus_leucogenys: 0.002557, Nomascus_siki: 0.002557): 0.003729, Nomascus_gabriellae: 0.006287): 0.002007, Nomascus_concolor: 0.008294): 0.017669, (Nomascus_nasutus: 0.017686, Nomascus_hainanus: 0.017686): 0.008277): 0.021901): 0.012399): 0.086534): 0.077968, ((((Macaca_sylvanus: 0.032701, ((((Macaca_nemestrina: 0.012476, Macaca_silenus: 0.012476): 0.003553, (Macaca_pagensis: 0.012010, (Macaca_siberu: 0.006068, Macaca_leonina: 0.006068): 0.005942): 0.004019): 0.004739, ((Macaca_hecki: 0.012012, (Macaca_nigrescens: 0.011054, (Macaca_tonkeana: 0.009535, Macaca_nigra: 0.009535): 0.001519): 0.000958): 0.000453, (Macaca_maura: 0.010925, (Macaca_ochreata: 0.005379, Macaca_brunnescens: 0.005379): 0.005546): 0.001540): 0.008303): 0.006904, ((Macaca_arctoides: 0.019764, (((Macaca_thibetana: 0.006054, Macaca_assamensis: 0.006054): 0.007145, Macaca_sinica: 0.013199): 0.003216, (Macaca_radiata: 0.009655, Macaca_munzala: 0.009655): 0.006760): 0.003350): 0.003450, ((Macaca_cyclopis: 0.013437, (Macaca_mulatta: 0.012331, Macaca_fuscata: 0.012331): 0.001106): 0.006696, Macaca_fascicularis: 0.020132): 0.003083): 0.004457): 0.005028): 0.024762, ((Theropithecus_gelada: 0.035421, (((Papio_papio: 0.009355, (Papio_hamadryas: 0.008486, ((Papio_cynocephalus: 0.004940, Papio_ursinus: 0.004940): 0.002698, Papio_anubis: 0.007638): 0.000849): 0.000869): 0.007114, Rungwecebus_kipunji: 0.016469): 0.010758, (Lophocebus_albigena: 0.002131, Lophocebus_aterrimus: 0.002131): 0.025096): 0.008194): 0.013686, ((Mandrillus_leucophaeus: 0.008838, Mandrillus_sphinx: 0.008838): 0.024419, ((Cercocebus_chrysogaster: 0.003078, (Cercocebus_galeritus: 0.001612, Cercocebus_agilis: 0.001612): 0.001466): 0.023005, (Cercocebus_atys: 0.010445, Cercocebus_torquatus: 0.010445): 0.015638): 0.007174): 0.015849): 0.008356): 0.017253, ((((Cercopithecus_campbelli: 0.026959, (Chlorocebus_cynosuros: 0.011413, (Chlorocebus_sabaeus: 0.010926, (Chlorocebus_pygerythrus: 0.008428, (Chlorocebus_aethiops: 0.006820, Chlorocebus_tantalus: 0.006820): 0.001607): 0.002498): 0.000487): 0.015546): 0.006214, ((Cercopithecus_solatus: 0.017842, (Cercopithecus_preussi: 0.008709, Cercopithecus_lhoesti: 0.008709): 0.009132): 0.013164, Erythrocebus_patas: 0.031006): 0.002167): 0.013073, (Cercopithecus_hamlyni: 0.034448, ((Cercopithecus_neglectus: 0.030465, (Cercopithecus_mona: 0.016384, (Cercopithecus_pogonias: 0.009274, Cercopithecus_wolfi: 0.009274): 0.007111): 0.014081): 0.002205, (Cercopithecus_diana: 0.029823, ((Cercopithecus_nictitans: 0.017209, (Cercopithecus_albogularis: 0.004636, Cercopithecus_mitis: 0.004636): 0.012573): 0.005423, ((Cercopithecus_ascanius: 0.012403, (Cercopithecus_erythrogaster: 0.009114, (Cercopithecus_erythrotis: 0.004730, Cercopithecus_cephus: 0.004730): 0.004383): 0.003289): 0.007237, Cercopithecus_petaurista: 0.019640): 0.002992): 0.007190): 0.002848): 0.001778): 0.011797): 0.004520, (Allenopithecus_nigroviridis: 0.048103, (Miopithecus_ogouensis: 0.025637, Miopithecus_talapoin: 0.025637): 0.022466): 0.002663): 0.023950): 0.039131, ((((((Semnopithecus_priam: 0.007882, Trachypithecus_vetulus: 0.007882): 0.010067, (((Semnopithecus_hector: 0.007961, Semnopithecus_entellus: 0.007961): 0.005642, Trachypithecus_johnii: 0.013602): 0.002657, (Trachypithecus_geei: 0.003039, Trachypithecus_pileatus: 0.003039): 0.013221): 0.001689): 0.011143, ((Trachypithecus_laotum: 0.009585, (Trachypithecus_hatinhensis: 0.004130, (Trachypithecus_delacouri: 0.003375, (Trachypithecus_francoisi: 0.002553, Trachypithecus_poliocephalus: 0.002553): 0.000822): 0.000755): 0.005455): 0.004945, ((Trachypithecus_auratus: 0.006692, (Trachypithecus_cristatus: 0.005135, Trachypithecus_germaini: 0.005135): 0.001557): 0.005748, ((Trachypithecus_barbei: 0.006591, Trachypithecus_obscurus: 0.006591): 0.004178, Trachypithecus_phayrei: 0.010769): 0.001671): 0.002090): 0.014561): 0.027072, (((Rhinopithecus_bieti: 0.022650, (Rhinopithecus_roxellana: 0.016772, Rhinopithecus_avunculus: 0.016772): 0.005877): 0.003118, Rhinopithecus_brelichi: 0.025768): 0.020239, ((Simias_concolor: 0.019892, Nasalis_larvatus: 0.019892): 0.024023, (Pygathrix_nigripes: 0.010268, (Pygathrix_cinerea: 0.003192, Pygathrix_nemaeus: 0.003192): 0.007076): 0.033646): 0.002093): 0.010156): 0.001735, (((Presbytis_siberu: 0.010139, Presbytis_potenziani: 0.010139): 0.008305, ((Presbytis_sumatranu: 0.008324, ((Presbytis_bicolor: 0.005449, Presbytis_rubicunda: 0.005449): 0.001507, Presbytis_melalophos: 0.006957): 0.001367): 0.001655, ((Presbytis_fredericae: 0.005261, Presbytis_comata: 0.005261): 0.002859, Presbytis_mitrata: 0.008120): 0.001858): 0.008465): 0.016949, ((Presbytis_hosei: 0.027627, (Presbytis_frontata: 0.021413, Presbytis_chrysomelas: 0.021413): 0.006214): 0.005303, (Presbytis_thomasi: 0.026733, Presbytis_femoralis: 0.026733): 0.006198): 0.002462): 0.022505): 0.021035, ((((((Piliocolobus_foai: 0.011909, (Piliocolobus_tephrosceles: 0.008546, Piliocolobus_rufomitratus: 0.008546): 0.003363): 0.007700, (Piliocolobus_kirkii: 0.009897, Piliocolobus_gordonorum: 0.009897): 0.009712): 0.009926, ((Piliocolobus_pennantii: 0.004614, Piliocolobus_preussi: 0.004614): 0.018207, Piliocolobus_tholloni: 0.022821): 0.006714): 0.003795, Piliocolobus_badius: 0.033330): 0.019147, Procolobus_verus: 0.052477): 0.007319, (Colobus_satanas: 0.032351, (((Colobus_polykomos: 0.003329, Colobus_vellerosus: 0.003329): 0.012127, Colobus_guereza: 0.015456): 0.005115, Colobus_angolensis: 0.020571): 0.011779): 0.027446): 0.019137): 0.034913): 0.110917): 0.152306): 0.208320): 0.119624): 0.097418): 0.064265, (Lagomorpha: 0.851031, (Tupaia_glis: 0.125509, Tupaia_minor: 0.125509): 0.725522): 0.015664);

4. Newick timetree with independent rates and soft-bounded constraints. One unit = 100 million years.

(((Galeopterus_variegatus: 0.117777, Cynocephalus_volans: 0.117777): 0.677118, (((((Galago_thomasi: 0.128137, (Galago_demidoff: 0.125971, ((Galago_matschiei: 0.022025, (Galago_moholi: 0.007230, (Galago_gallarum: 0.005977, Galago_senegalensis: 0.005977): 0.001253): 0.014795): 0.079954, ((Galago_orinus: 0.036275, (Galago_granti: 0.011450, Galago_zanzibaricus: 0.011450): 0.024825): 0.042659, ((Otolemur_monteiri: 0.047559, (Otolemur_crassicaudatus: 0.027925, Otolemur_garnettii: 0.027925): 0.019634): 0.009439, (Galago_gabonensis: 0.003143, Galago_alleni: 0.003143): 0.053854): 0.021937): 0.023045): 0.023992): 0.002166): 0.099850, Euoticus_elegantulus: 0.227987): 0.099641, (((Loris_lydekkerianus: 0.003022, Loris_tardigradus: 0.003022): 0.160722, (Nycticebus_pygmaeus: 0.046885, (Nycticebus_menagensis: 0.020397, (Nycticebus_javanicus: 0.008682, (Nycticebus_bengalensis: 0.002726, Nycticebus_coucang: 0.002726): 0.005956): 0.011715): 0.026488): 0.116860): 0.141996, (Perodicticus_potto: 0.178426, (Arctocebus_calabarensis: 0.005874, Arctocebus_aureus: 0.005874): 0.172552): 0.127314): 0.021888): 0.221472, (Daubentonia_madagascariensis: 0.491954, (((Varecia_rubra: 0.005666, Varecia_variegata: 0.005666): 0.178755, ((Lemur_catta: 0.056517, (Prolemur_simus: 0.047452, (Hapalemur_aureus: 0.026504, (Hapalemur_occidentalis: 0.006834, (Hapalemur_alaotrensis: 0.003560, (Hapalemur_griseus: 0.002041, Hapalemur_meridionalis: 0.002041): 0.001520): 0.003273): 0.019671): 0.020948): 0.009065): 0.103406, (((((Eulemur_rufifrons: 0.012339, (Eulemur_fulvus: 0.008420, (Eulemur_albifrons: 0.005645, Eulemur_sanfordi: 0.005645): 0.002774): 0.003919): 0.004223, ((Eulemur_cinereiceps: 0.005648, Eulemur_collaris: 0.005648): 0.008409, Eulemur_rufus: 0.014057): 0.002505): 0.014921, Eulemur_mongoz: 0.031482): 0.005049, ((Eulemur_macaco: 0.005846, Eulemur_flavifrons: 0.005846): 0.027108, Eulemur_coronatus: 0.032954): 0.003577): 0.014022, Eulemur_rubriventer: 0.050553): 0.109371): 0.024497): 0.113546, (((Phaner_pallescens: 0.209337, ((((Lepilemur_aeeclis: 0.023901, (Lepilemur_randrianasoloi: 0.017868, (Lepilemur_hubbardorum: 0.010052, Lepilemur_ruficaudatus: 0.010052): 0.007817): 0.006033): 0.015606, (Lepilemur_leucopus: 0.007583, Lepilemur_petteri: 0.007583): 0.031924): 0.010532, ((Lepilemur_microdon: 0.021514, (Lepilemur_otto: 0.010652, (Lepilemur_grewcockorum: 0.007388, Lepilemur_edwardsi: 0.007388): 0.003263): 0.010863): 0.012926, (Lepilemur_septentrionalis: 0.018626, ((Lepilemur_ankaranensis: 0.009148, (Lepilemur_tymerlachsoni: 0.007716, Lepilemur_milanoii: 0.007716): 0.001432): 0.006158, ((Lepilemur_mittermeieri: 0.011145, (Lepilemur_sahamalazensis: 0.007184, Lepilemur_ahmansoni: 0.007184): 0.003961): 0.003066, Lepilemur_dorsalis: 0.014211): 0.001095): 0.003320): 0.015815): 0.015599): 0.022869, (Lepilemur_seali: 0.042334, (Lepilemur_wrighti: 0.027561, (Lepilemur_fleuretae: 0.016283, (Lepilemur_mustelinus: 0.010150, (Lepilemur_betsileo: 0.006170, Lepilemur_jamesorum: 0.006170): 0.003980): 0.006133): 0.011278): 0.014773): 0.030574): 0.136429): 0.048300, ((Allocebus_trichotis: 0.140392, ((Mirza_coquereli: 0.002696, Mirza_zaza: 0.002696): 0.113906, ((Microcebus_griseorufus: 0.034391, Microcebus_murinus: 0.034391): 0.029801, (((Microcebus_macarthurii: 0.016767, Microcebus_sp_nova3Radespiel08: 0.016767): 0.017816, (Microcebus_danfossi: 0.021688, (Microcebus_ravelobensis: 0.007018, Microcebus_bongolavensis: 0.007018): 0.014670): 0.012895): 0.018019, ((((Microcebus_rufus: 0.028287, ((Microcebus_sambiranensis: 0.010577, Microcebus_arnholdi: 0.010577): 0.011490, (Microcebus_mamiratra: 0.009093, Microcebus_margotmarshae: 0.009093): 0.012974): 0.006220): 0.003821, (((Microcebus_myoxinus: 0.010139, Microcebus_berthae: 0.010139): 0.011554, Microcebus_lehilahytsara: 0.021693): 0.007521, Microcebus_mittermeieri: 0.029214): 0.002894): 0.004046, (Microcebus_simmonsi: 0.031645, (((Microcebus_jollyae: 0.001661, (Microcebus_sp_nova1Louis06: 0.001377, Microcebus_sp_nova3Louis06: 0.001377): 0.000285): 0.017652, (Microcebus_gerpi: 0.008409, Microcebus_spDWW2010aMarolambo: 0.008409): 0.010904): 0.005462, Microcebus_spDWW2010aManantantel: 0.024775): 0.006870): 0.004509): 0.008120, Microcebus_tavaratra: 0.044274): 0.008328): 0.011590): 0.052411): 0.023789): 0.058354, (Cheirogaleus_sibreei: 0.076142, (Cheirogaleus_crossleyi: 0.057785, (Cheirogaleus_major: 0.046820, Cheirogaleus_medius: 0.046820): 0.010966): 0.018356): 0.122605): 0.058891): 0.028783, (Indri_indri: 0.156182, (((((Avahi_ramanantsoavani: 0.005936, Avahi_meridionalis: 0.005936): 0.005594, (Avahi_peyrierasi: 0.003728, Avahi_betsilio: 0.003728): 0.007802): 0.006446, Avahi_laniger: 0.017975): 0.030993, (Avahi_occidentalis: 0.013502, (Avahi_cleesei: 0.011329, Avahi_unicolor: 0.011329): 0.002173): 0.035466): 0.076767, ((Propithecus_perrieri: 0.019085, (Propithecus_edwardsi: 0.014006, Propithecus_diadema: 0.014006): 0.005079): 0.028998, ((Propithecus_tattersalli: 0.014427, Propithecus_coquereli: 0.014427): 0.005160, Propithecus_verreauxi: 0.019587): 0.028496): 0.077653): 0.030446): 0.130239): 0.011546): 0.193988): 0.057146): 0.164445, (((Tarsius_dentatus: 0.023840, ((Tarsius_wallacei: 0.009620, Tarsius_tarsier: 0.009620): 0.008075, (Tarsius_sangirensis: 0.012660, Tarsius_lariang: 0.012660): 0.005035): 0.006145): 0.107720, (Tarsius_bancanus: 0.071641, Tarsius_syrichta: 0.071641): 0.059919): 0.510234, ((((((Cacajao_calvus: 0.021866, (Cacajao_melanocephalus: 0.011163, (Cacajao_hosomi: 0.001890, Cacajao_ayresi: 0.001890): 0.009274): 0.010702): 0.033088, ((Chiropotes_israelita: 0.010803, Chiropotes_chiropotes: 0.010803): 0.008119, Chiropotes_utahicki: 0.018923): 0.036031): 0.062258, (Pithecia_pithecia: 0.028776, (Pithecia_monachus: 0.003703, Pithecia_irrorata: 0.003703): 0.025073): 0.088436): 0.063894, ((Callicebus_lugens: 0.014882, Callicebus_torquatus: 0.014882): 0.056207, (((Callicebus_personatus: 0.018492, Callicebus_coimbrai: 0.018492): 0.012749, Callicebus_nigrifrons: 0.031241): 0.029648, ((Callicebus_cupreus: 0.018135, (Callicebus_brunneus: 0.016260, (Callicebus_moloch: 0.012092, Callicebus_hoffmannsi: 0.012092): 0.004169): 0.001874): 0.009011, (Callicebus_caligatus: 0.005049, Callicebus_donacophilus: 0.005049): 0.022096): 0.033744): 0.010200): 0.110017): 0.034283, ((((Alouatta_pigra: 0.014905, (Alouatta_palliata: 0.003387, Alouatta_coibensis: 0.003387): 0.011519): 0.015029, ((Alouatta_caraya: 0.022653, (Alouatta_sara: 0.018009, (Alouatta_nigerrima: 0.004260, (Alouatta_seniculus: 0.000417, Alouatta_macconnelli: 0.000417): 0.003842): 0.013749): 0.004644): 0.002419, (Alouatta_guariba: 0.017626, Alouatta_belzebul: 0.017626): 0.007446): 0.004862): 0.095079, (((Brachyteles_hypoxanthus: 0.019123, Brachyteles_arachnoides: 0.019123): 0.059458, ((Lagothrix_cana: 0.003849, (Lagothrix_lagotricha: 0.002941, Lagothrix_poeppigii: 0.002941): 0.000907): 0.006061, Lagothrix_lugens: 0.009910): 0.068672): 0.010121, ((Ateles_fusciceps: 0.012515, (Ateles_hybridus: 0.012160, (Ateles_belzebuth: 0.006812, Ateles_geoffroyi: 0.006812): 0.005347): 0.000355): 0.007351, (Ateles_paniscus: 0.006740, (Ateles_marginatus: 0.003288, Ateles_chamek: 0.003288): 0.003453): 0.013125): 0.068836): 0.036312): 0.078320, ((((Saimiri_ustus: 0.007924, (Saimiri_sciureus: 0.004661, Saimiri_oerstedii: 0.004661): 0.003262): 0.006575, Saimiri_boliviensis: 0.014498): 0.143469, (((Cebus_libidinosus: 0.002312, Cebus_apella: 0.002312): 0.010175, (Cebus_xanthosternos: 0.010297, Cebus_robustus: 0.010297): 0.002190): 0.031704, (Cebus_capucinus: 0.013192, ((Cebus_kaapori: 0.004565, Cebus_olivaceus: 0.004565): 0.007619, Cebus_albifrons: 0.012184): 0.001008): 0.030999): 0.113776): 0.022722, ((((Saguinus_fuscicollis: 0.024047, ((Saguinus_melanoleucus: 0.013727, (Saguinus_graellsi: 0.002242, Saguinus_nigricollis: 0.002242): 0.011485): 0.003113, Saguinus_tripartitus: 0.016839): 0.007208): 0.032151, (((Saguinus_oedipus: 0.004979, Saguinus_geoffroyi: 0.004979): 0.032814, ((Saguinus_bicolor: 0.007562, Saguinus_martinsi: 0.007562): 0.012917, (Saguinus_niger: 0.009405, Saguinus_midas: 0.009405): 0.011074): 0.017314): 0.010372, ((Saguinus_imperator: 0.024008, (Saguinus_labiatus: 0.013340, Saguinus_mystax: 0.013340): 0.010668): 0.009437, Saguinus_leucopus: 0.033445): 0.014720): 0.008034): 0.069930, (((Leontopithecus_rosalia: 0.001191, Leontopithecus_chrysopygus: 0.001191): 0.002882, Leontopithecus_chrysomelas: 0.004073): 0.116132, (Callimico_goeldii: 0.100564, (Callithrix_saterei: 0.053951, ((Callithrix_aurita: 0.024137, (Callithrix_geoffroyi: 0.007710, (Callithrix_kuhlii: 0.007058, (Callithrix_jacchus: 0.004396, Callithrix_penicillata: 0.004396): 0.002662): 0.000652): 0.016426): 0.016095, ((Callithrix_argentata: 0.014885, (Callithrix_emiliae: 0.009149, (Callithrix_mauesi: 0.005994, Callithrix_humeralifera: 0.005994): 0.003155): 0.005735): 0.018877, (Callithrix_humilis: 0.016330, Callithrix_pygmaea: 0.016330): 0.017431): 0.006469): 0.013720): 0.046613): 0.019641): 0.005923): 0.050936, ((Aotus_vociferans: 0.020004, ((Aotus_lemurinus: 0.009958, Aotus_griseimembra: 0.009958): 0.007565, Aotus_trivirgatus: 0.017523): 0.002481): 0.006665, (Aotus_nancymaae: 0.021975, (Aotus_nigriceps: 0.010209, (Aotus_infulatus: 0.003422, Aotus_azarae: 0.003422): 0.006787): 0.011766): 0.004694): 0.150396): 0.003625): 0.022645): 0.012055): 0.158745, ((((Pongo_abelii: 0.008204, Pongo_pygmaeus: 0.008204): 0.112549, (((Pan_troglodytes: 0.010163, Pan_paniscus: 0.010163): 0.044215, Homo_sapiens: 0.054378): 0.008648, (Gorilla_beringei: 0.009318, Gorilla_gorilla: 0.009318): 0.053708): 0.057727): 0.015268, ((Hoolock_hoolock: 0.011684, Hoolock_leuconedys: 0.011684): 0.039955, ((Symphalangus_syndactylus: 0.037975, (Hylobates_pileatus: 0.020917, (((Hylobates_klossii: 0.011425, Hylobates_moloch: 0.011425): 0.002975, ((Hylobates_agilis: 0.005729, Hylobates_albibarbis: 0.005729): 0.006384, (Hylobates_abbotti: 0.007903, (Hylobates_funereus: 0.005344, Hylobates_muelleri: 0.005344): 0.002559): 0.004210): 0.002286): 0.002049, Hylobates_lar: 0.016448): 0.004468): 0.017059): 0.003439, ((((Nomascus_leucogenys: 0.002298, Nomascus_siki: 0.002298): 0.003345, Nomascus_gabriellae: 0.005643): 0.001754, Nomascus_concolor: 0.007397): 0.013256, (Nomascus_nasutus: 0.013737, Nomascus_hainanus: 0.013737): 0.006915): 0.020762): 0.010225): 0.084382): 0.076778, ((((Macaca_sylvanus: 0.027393, ((((Macaca_nemestrina: 0.010686, Macaca_silenus: 0.010686): 0.003115, (Macaca_pagensis: 0.010498, (Macaca_siberu: 0.005581, Macaca_leonina: 0.005581): 0.004917): 0.003303): 0.003838, ((Macaca_hecki: 0.010317, (Macaca_nigrescens: 0.009475, (Macaca_tonkeana: 0.008221, Macaca_nigra: 0.008221): 0.001254): 0.000841): 0.000398, (Macaca_maura: 0.009369, (Macaca_ochreata: 0.004546, Macaca_brunnescens: 0.004546): 0.004823): 0.001346): 0.006924): 0.005602, ((Macaca_arctoides: 0.016762, (((Macaca_thibetana: 0.005260, Macaca_assamensis: 0.005260): 0.005817, Macaca_sinica: 0.011077): 0.002748, (Macaca_radiata: 0.007961, Macaca_munzala: 0.007961): 0.005864): 0.002937): 0.002906, ((Macaca_cyclopis: 0.011610, (Macaca_mulatta: 0.010670, Macaca_fuscata: 0.010670): 0.000940): 0.005511, Macaca_fascicularis: 0.017121): 0.002547): 0.003573): 0.004152): 0.017742, ((Theropithecus_gelada: 0.023565, (((Papio_papio: 0.007569, (Papio_hamadryas: 0.006925, ((Papio_cynocephalus: 0.004043, Papio_ursinus: 0.004043): 0.002192, Papio_anubis: 0.006235): 0.000690): 0.000643): 0.004991, Rungwecebus_kipunji: 0.012560): 0.006763, (Lophocebus_albigena: 0.001750, Lophocebus_aterrimus: 0.001750): 0.017573): 0.004243): 0.014222, ((Mandrillus_leucophaeus: 0.007394, Mandrillus_sphinx: 0.007394): 0.019580, ((Cercocebus_chrysogaster: 0.002582, (Cercocebus_galeritus: 0.001403, Cercocebus_agilis: 0.001403): 0.001178): 0.018570, (Cercocebus_atys: 0.008526, Cercocebus_torquatus: 0.008526): 0.012625): 0.005822): 0.010813): 0.007348): 0.016952, ((((Cercopithecus_campbelli: 0.021641, (Chlorocebus_cynosuros: 0.009595, (Chlorocebus_sabaeus: 0.009175, (Chlorocebus_pygerythrus: 0.007083, (Chlorocebus_aethiops: 0.005668, Chlorocebus_tantalus: 0.005668): 0.001414): 0.002093): 0.000420): 0.012046): 0.006769, ((Cercopithecus_solatus: 0.014410, (Cercopithecus_preussi: 0.006600, Cercopithecus_lhoesti: 0.006600): 0.007810): 0.012017, Erythrocebus_patas: 0.026427): 0.001983): 0.011313, (Cercopithecus_hamlyni: 0.030614, ((Cercopithecus_neglectus: 0.026620, (Cercopithecus_mona: 0.014530, (Cercopithecus_pogonias: 0.006647, Cercopithecus_wolfi: 0.006647): 0.007883): 0.012091): 0.002082, (Cercopithecus_diana: 0.025991, ((Cercopithecus_nictitans: 0.014792, (Cercopithecus_albogularis: 0.003869, Cercopithecus_mitis: 0.003869): 0.010922): 0.004706, ((Cercopithecus_ascanius: 0.010929, (Cercopithecus_erythrogaster: 0.007607, (Cercopithecus_erythrotis: 0.004071, Cercopithecus_cephus: 0.004071): 0.003536): 0.003321): 0.006056, Cercopithecus_petaurista: 0.016984): 0.002513): 0.006493): 0.002711): 0.001912): 0.009109): 0.003582, (Allenopithecus_nigroviridis: 0.041101, (Miopithecus_ogouensis: 0.021251, Miopithecus_talapoin: 0.021251): 0.019851): 0.002204): 0.018781): 0.038587, ((((((Semnopithecus_priam: 0.006898, Trachypithecus_vetulus: 0.006898): 0.008650, (((Semnopithecus_hector: 0.006772, Semnopithecus_entellus: 0.006772): 0.004951, Trachypithecus_johnii: 0.011724): 0.002321, (Trachypithecus_geei: 0.002754, Trachypithecus_pileatus: 0.002754): 0.011291): 0.001503): 0.009269, ((Trachypithecus_laotum: 0.008679, (Trachypithecus_hatinhensis: 0.003698, (Trachypithecus_delacouri: 0.003005, (Trachypithecus_francoisi: 0.002201, Trachypithecus_poliocephalus: 0.002201): 0.000804): 0.000693): 0.004981): 0.003963, ((Trachypithecus_auratus: 0.005742, (Trachypithecus_cristatus: 0.004330, Trachypithecus_germaini: 0.004330): 0.001412): 0.004969, ((Trachypithecus_barbei: 0.005838, Trachypithecus_obscurus: 0.005838): 0.003404, Trachypithecus_phayrei: 0.009242): 0.001469): 0.001932): 0.012174): 0.025247, (((Rhinopithecus_bieti: 0.017493, (Rhinopithecus_roxellana: 0.013036, Rhinopithecus_avunculus: 0.013036): 0.004458): 0.002545, Rhinopithecus_brelichi: 0.020038): 0.020572, ((Simias_concolor: 0.016631, Nasalis_larvatus: 0.016631): 0.022146, (Pygathrix_nigripes: 0.009336, (Pygathrix_cinerea: 0.002900, Pygathrix_nemaeus: 0.002900): 0.006437): 0.029440): 0.001834): 0.009454): 0.001733, (((Presbytis_siberu: 0.008846, Presbytis_potenziani: 0.008846): 0.007121, ((Presbytis_sumatranu: 0.007412, ((Presbytis_bicolor: 0.004805, Presbytis_rubicunda: 0.004805): 0.001323, Presbytis_melalophos: 0.006128): 0.001284): 0.001501, ((Presbytis_fredericae: 0.004616, Presbytis_comata: 0.004616): 0.002667, Presbytis_mitrata: 0.007283): 0.001630): 0.007054): 0.017138, ((Presbytis_hosei: 0.023370, (Presbytis_frontata: 0.017503, Presbytis_chrysomelas: 0.017503): 0.005867): 0.006505, (Presbytis_thomasi: 0.023041, Presbytis_femoralis: 0.023041): 0.006834): 0.003230): 0.018692): 0.018566, ((((((Piliocolobus_foai: 0.009918, (Piliocolobus_tephrosceles: 0.006944, Piliocolobus_rufomitratus: 0.006944): 0.002974): 0.006759, (Piliocolobus_kirkii: 0.008198, Piliocolobus_gordonorum: 0.008198): 0.008480): 0.009229, ((Piliocolobus_pennantii: 0.003760, Piliocolobus_preussi: 0.003760): 0.014849, Piliocolobus_tholloni: 0.018608): 0.007298): 0.004142, Piliocolobus_badius: 0.030048): 0.016991, Procolobus_verus: 0.047039): 0.006799, (Colobus_satanas: 0.031251, (((Colobus_polykomos: 0.003002, Colobus_vellerosus: 0.003002): 0.010682, Colobus_guereza: 0.013683): 0.004365, Colobus_angolensis: 0.018048): 0.013203): 0.022588): 0.016525): 0.030310): 0.112126): 0.161335): 0.267659): 0.071752): 0.081349): 0.051301, (Lagomorpha: 0.830050, (Tupaia_glis: 0.113546, Tupaia_minor: 0.113546): 0.716504): 0.016146);

5. RAxML phylogram based on the 61199 bp concatenation of 69 nuclear and ten mitochondrial genes.

(((Galeopterus_variegatus:0.024460111494431558,Cynocephalus_volans:0.028412268735723223):0.12855725393225897,(((((Galago_thomasi:0.036201671626704734,(Galago_demidoff:0.026373213854154254,((Galago_matschiei:0.006489708261560256,(Galago_moholi:0.0024117777747945657,(Galago_gallarum:0.006699937316268334,Galago_senegalensis:8.999445691542096E-4):0.00142009464768178):0.0112098033490306):0.013024142283295292,((Galago_orinus:0.02123035351202862,(Galago_granti:0.0015876667505905773,Galago_zanzibaricus:0.004740002258704912):0.009330349877413269):0.004081089804949478,((Otolemur_monteiri:0.006370278893536363,(Otolemur_crassicaudatus:0.005226662541199856,Otolemur_garnettii:0.003643459699747831):0.010280737628535375):0.003130098024309358,(Galago_gabonensis:3.869519049293224E-6,Galago_alleni:0.001488226250031477):0.015638862039821677):0.004884232320691295):0.001983765537270532):0.004872175440242654):7.25504053598891E-4):0.010419291890286064,Euoticus_elegantulus:0.02781128270301636):0.022901086237258228,(((Loris_lydekkerianus:3.869519049293224E-6,Loris_tardigradus:3.869519049293224E-6):0.040226317557717994,(Nycticebus_pygmaeus:0.013596357981513207,(Nycticebus_menagensis:0.007265912929541207,(Nycticebus_javanicus:0.003365266316823101,(Nycticebus_bengalensis:6.019772570287074E-4,Nycticebus_coucang:8.020248447127565E-4):0.0014388099702713486):0.002442779298310438):0.008213559297947426):0.026425721927252066):0.02708288516494739,(Perodicticus_potto:0.033709122727562546,(Arctocebus_calabarensis:3.869519049293224E-6,Arctocebus_aureus:8.903661082684811E-4):0.044521284847671094):0.028234269516780652):0.00439772630113916):0.10865772755783282,(Daubentonia_madagascariensis:0.07498935424376574,(((Varecia_rubra:0.0026255285012658502,Varecia_variegata:0.0017710598812168965):0.031460949456281195,((Lemur_catta:0.013227228720332218,(Prolemur_simus:0.01607963294950787,(Hapalemur_aureus:0.010830532493154077,(Hapalemur_occidentalis:0.0025754895708267056,(Hapalemur_alaotrensis:2.284210796829611E-4,(Hapalemur_griseus:3.869519049293224E-6,Hapalemur_meridionalis:0.013562508922382976):0.0015225444618189865):0.0012509859101434428):0.008817150433806265):0.004630429239763922):0.0014524324379868725):0.016134395673688073,(((((Eulemur_rufifrons:0.0023617477813132615,(Eulemur_fulvus:0.0014371252009461877,(Eulemur_albifrons:0.0011909751139957403,Eulemur_sanfordi:0.0019982037679092124):6.513740465649009E-4):9.271674812598718E-4):8.04056694069355E-4,((Eulemur_cinereiceps:0.002316397920177793,Eulemur_collaris:0.0014144620668898322):0.0027034340150114966,Eulemur_rufus:0.0027107900961115816):6.874427962038632E-4):0.003941594832844231,Eulemur_mongoz:0.008703312834016097):0.0014081073763704821,((Eulemur_macaco:0.002564187470257273,Eulemur_flavifrons:0.0032062684101942796):0.006232548275228511,Eulemur_coronatus:0.009291419833456549):0.0014306495710285638):0.0021440287675187353,Eulemur_rubriventer:0.009334161858743861):0.01657453315756824):0.006274290188726328):0.01746604814590963,(((Phaner_pallescens:0.06102841467618281,((((Lepilemur_aeeclis:0.011442698338215374,(Lepilemur_randrianasoloi:0.0045503245596650865,(Lepilemur_hubbardorum:0.0071108625853908425,Lepilemur_ruficaudatus:0.002451075113619583):0.001974751247387274):0.0020705631557333404):0.004648381711761684,(Lepilemur_leucopus:0.0011841874524946006,Lepilemur_petteri:0.002440875901826789):0.01279036859654853):0.0028460687447241506,((Lepilemur_microdon:0.0071346589783709,(Lepilemur_otto:0.003288441124635666,(Lepilemur_grewcockorum:0.00305808906283489,Lepilemur_edwardsi:0.0014527235296662444):4.634290427867338E-4):0.006032948816871719):0.0028889929366331946,(Lepilemur_septentrionalis:0.007982004488058803,((Lepilemur_ankaranensis:0.0012657006094698442,(Lepilemur_tymerlachsoni:0.003455421305870232,Lepilemur_milanoii:0.002033664242216071):5.799020931804844E-4):0.0021473550916266126,((Lepilemur_mittermeieri:0.0044797735755287005,(Lepilemur_sahamalazensis:0.003141430143690227,Lepilemur_ahmansoni:0.003488337708585032):0.0013125145660095039):0.0012808352859899896,Lepilemur_dorsalis:0.004285183125797121):4.118651054064726E-4):0.0037104283471069976):0.004128912332315937):0.0037013651917697787):0.008478864568596312,(Lepilemur_seali:0.01624468497498477,(Lepilemur_wrighti:0.008350195716025688,(Lepilemur_fleuretae:0.005322677673635923,(Lepilemur_mustelinus:0.005406781318979426,(Lepilemur_betsileo:9.154180857975369E-4,Lepilemur_jamesorum:0.0026826192945244576):8.380883228578662E-4):0.0015202990871979183):0.004064695653432149):0.0032643031233591246):0.008099740635124808):0.033554336236647164):0.004478277693704791,((Allocebus_trichotis:0.026274161120755812,((Mirza_coquereli:0.0011880219879467635,Mirza_zaza:4.688145787035136E-4):0.0340162260120776,((Microcebus_griseorufus:0.00986330337964142,Microcebus_murinus:0.011004217050325116):0.005387323074369954,(((Microcebus_macarthurii:0.00530179849397544,Microcebus_sp_nova3Radespiel08:0.006024482664444053):0.0040964489437108065,(Microcebus_danfossi:0.0058036614790234475,(Microcebus_ravelobensis:0.0028977213330029783,Microcebus_bongolavensis:0.0010386548444438204):0.005833917160502411):0.002144708435525944):0.00220391629958816,((((Microcebus_rufus:0.003795749472232768,((Microcebus_sambiranensis:0.002635924044600557,Microcebus_arnholdi:0.0026953961907491553):0.004405651581910475,(Microcebus_mamiratra:0.0020469978445299963,Microcebus_margotmarshae:0.0025297565635049035):0.003630358316825455):0.0010827577149475354):0.0016201822719874581,(((Microcebus_myoxinus:0.004427800415802929,Microcebus_berthae:0.0032141953789174282):0.0011859507416616122,Microcebus_lehilahytsara:0.004725781024508546):0.0019666138670138,Microcebus_mittermeieri:0.006226127797839154):8.453498005052684E-4):0.0010274769395874905,(Microcebus_simmonsi:0.007513483294244783,(((Microcebus_jollyae:5.548896653884139E-5,(Microcebus_sp_nova1Louis06:0.002829974549531933,Microcebus_sp_nova3Louis06:3.869519049293224E-6):0.001432779669616302):0.0026556742252243914,(Microcebus_gerpi:0.004312573395670205,Microcebus_spDWW2010aMarolambo:0.003622620226688078):0.0083712100798638):9.820450500686029E-4,Microcebus_spDWW2010aManantantel:0.0073624824285386525):0.0012477682274721347):9.368742978276279E-4):0.0013760403217325523,Microcebus_tavaratra:0.010807748417842156):0.0026863478403246766):0.0046928668318251265):0.015748315399865598):0.00713090727460286):0.015381074925101157,(Cheirogaleus_sibreei:0.014617376627814482,(Cheirogaleus_crossleyi:0.013188256621701477,(Cheirogaleus_major:0.007285533127930455,Cheirogaleus_medius:0.01168207032155305):0.004541271652717727):0.005622200518451248):0.013966995903222312):0.01397792270096132):0.0063268424471802365,(Indri_indri:0.02491136599655941,(((((Avahi_ramanantsoavani:0.0017006998211907542,Avahi_meridionalis:0.001398058878864905):0.0011483228226087383,(Avahi_peyrierasi:7.232669247607126E-4,Avahi_betsilio:9.562516532063969E-4):0.0019569472486241635):0.0018206888113658781,Avahi_laniger:0.0037762563248130987):0.010444339398347713,(Avahi_occidentalis:0.003043645510386883,(Avahi_cleesei:0.0023560233196160407,Avahi_unicolor:0.0027824004024963322):2.3774167011092473E-4):0.009770622687750141):0.02171764148140959,((Propithecus_perrieri:0.0015967079516689409,(Propithecus_edwardsi:0.0028296727772075814,Propithecus_diadema:0.0029555194939079876):0.0015395774440559173):0.006962203861934968,((Propithecus_tattersalli:0.0022729525852934196,Propithecus_coquereli:0.0017246789206251667):0.003754248337494215,Propithecus_verreauxi:0.006500253415133883):0.005490181958915374):0.012213914877138743):0.008402503976762343):0.01665233689629081):0.003259712090164457):0.034335261110837956):0.011806746934640233):0.04147249737078823,(((Tarsius_dentatus:0.0013545943446121589,((Tarsius_wallacei:0.004083882520921067,Tarsius_tarsier:0.005024358756334668):0.001449646747348876,(Tarsius_sangirensis:0.01680053255196723,Tarsius_lariang:0.0028866896150784305):9.368416121796619E-4):0.0017841051891434034):0.013892256554290827,(Tarsius_bancanus:0.021502663009338452,Tarsius_syrichta:0.028555409421938682):0.0380915778962283):0.1145468090334309,((((((Cacajao_calvus:0.004507406316606066,(Cacajao_melanocephalus:0.0024807618548043364,(Cacajao_hosomi:4.83142716425633E-4,Cacajao_ayresi:3.048243278996887E-4):0.00236069232693914):0.004436961100114711):0.007239691937522941,((Chiropotes_israelita:0.0026974278996187984,Chiropotes_chiropotes:0.0014597848765103838):0.001907331188517991,Chiropotes_utahicki:0.001683086630595021):0.01101695280479742):0.011952700878113287,(Pithecia_pithecia:0.00731496412980015,(Pithecia_monachus:6.064845002322627E-4,Pithecia_irrorata:6.93707970763846E-4):0.00522711025600886):0.016557026906421246):0.013319979134395199,((Callicebus_lugens:0.00210157002703687,Callicebus_torquatus:0.0048861134150643415):0.006531333119858096,(((Callicebus_personatus:0.005246494852319594,Callicebus_coimbrai:0.004666082642012681):0.0033385284879134947,Callicebus_nigrifrons:0.007525928841211005):0.00788336809316148,((Callicebus_cupreus:0.0026103005571767035,(Callicebus_brunneus:0.013844467324818477,(Callicebus_moloch:0.0035617927224306234,Callicebus_hoffmannsi:0.006464575338829548):0.0018015869566951892):9.73598092041339E-4):0.00246039686640126,(Callicebus_caligatus:0.005244470214913921,Callicebus_donacophilus:0.0010363445633534973):0.006010077271485947):0.007689377225700178):0.0042695319294451806):0.019883097077316836):0.006550640072575442,((((Alouatta_pigra:0.004191257198543685,(Alouatta_palliata:7.660682793931839E-4,Alouatta_coibensis:6.35849947009659E-4):0.0033727346645367473):0.0036210560675711823,((Alouatta_caraya:0.006208493259261705,(Alouatta_sara:0.003697117014167528,(Alouatta_nigerrima:8.379580682573717E-4,(Alouatta_seniculus:3.869519049237713E-6,Alouatta_macconnelli:3.869519049237713E-6):7.24100555419438E-4):0.003633398507920771):0.0017552066682632872):0.0010083845253488488,(Alouatta_guariba:0.004300106528677017,Alouatta_belzebul:0.005573338116782456):9.22816272248328E-4):0.0014979190356621541):0.021709592779517672,(((Brachyteles_hypoxanthus:0.0074697412299088994,Brachyteles_arachnoides:0.003261930188309725):0.012812695976800015,((Lagothrix_cana:8.007866827982046E-4,(Lagothrix_lagotricha:0.0015117992954639115,Lagothrix_poeppigii:0.0025269484725422564):1.5673939071725318E-4):0.002148864645253712,Lagothrix_lugens:0.0010017742833797194):0.012027470426006548):0.0034876299356717544,((Ateles_fusciceps:0.0030283851809762563,(Ateles_hybridus:0.0046416592090461894,(Ateles_belzebuth:0.0032798650864113155,Ateles_geoffroyi:0.003837267849098769):9.037742148056971E-4):3.9824669822197123E-4):0.0014105315909749838,(Ateles_paniscus:0.002196019304650243,(Ateles_marginatus:0.0034504612908585752,Ateles_chamek:4.589805494756849E-4):0.0018832283182773635):0.003125282548946562):0.012623188653299755):0.006294798793733736):0.009839220959381612,((((Saimiri_ustus:0.004640349500674268,(Saimiri_sciureus:0.002083862759036492,Saimiri_oerstedii:0.002105958254085716):6.73665953416891E-4):0.0015433623480756165,Saimiri_boliviensis:0.004108488518295372):0.03776956363286332,(((Cebus_libidinosus:6.137243812863735E-4,Cebus_apella:9.221226113096392E-4):0.004303069911582136,(Cebus_xanthosternos:0.002180102509429127,Cebus_robustus:0.005156678112251234):0.0011048274735753205):0.006593783463563652,(Cebus_capucinus:0.0034862909914673557,((Cebus_kaapori:0.0015564913694956495,Cebus_olivaceus:0.0010259230634074301):0.0012522152858815416,Cebus_albifrons:0.003512843108928243):5.575242532275815E-4):0.0077831110110400115):0.022338388757073613):0.005035327332091555,((((Saguinus_fuscicollis:0.0031416686447821585,((Saguinus_melanoleucus:0.0036952159840861754,(Saguinus_graellsi:8.809780418620461E-4,Saguinus_nigricollis:2.096503644366532E-4):0.005333913174389804):7.514247160054222E-4,Saguinus_tripartitus:0.005596365067332965):0.0014282032633594621):0.010786473150400488,(((Saguinus_oedipus:0.0022385182627511213,Saguinus_geoffroyi:0.00125904628657314):0.009663151799238323,((Saguinus_bicolor:8.835598306995429E-4,Saguinus_martinsi:0.007148546180463233):0.004973426223240596,(Saguinus_niger:3.869519049237713E-6,Saguinus_midas:0.005487887986540596):0.0033583524446121893):0.005015259524467852):0.002676729544687395,((Saguinus_imperator:0.008354570276014273,(Saguinus_labiatus:0.003915739300302479,Saguinus_mystax:0.0030683700464822494):0.002809077033531282):0.002707414704699429,Saguinus_leucopus:0.015346568047209408):0.003154867493892155):0.0027609385634929096):0.01518099976304016,(((Leontopithecus_rosalia:5.521270960613389E-4,Leontopithecus_chrysopygus:7.439397112698476E-4):0.0016128033429343436,Leontopithecus_chrysomelas:0.0013873248490728907):0.030193859898558273,(Callimico_goeldii:0.02430776074885954,(Callithrix_saterei:0.018061457297109174,((Callithrix_aurita:0.008786306922025644,(Callithrix_geoffroyi:0.0018434344912997003,(Callithrix_kuhlii:0.005718196328693637,(Callithrix_jacchus:9.246995966565863E-4,Callithrix_penicillata:0.0012573028543774178):7.224226605176409E-4):3.181694301560567E-4):0.003660439772702029):0.0037324012945383345,((Callithrix_argentata:0.00332907451254022,(Callithrix_emiliae:3.869519049237713E-6,(Callithrix_mauesi:9.739238416790474E-4,Callithrix_humeralifera:0.0028146680785618994):3.8672063808325996E-4):0.00313489171537612):0.0057083021258672595,(Callithrix_humilis:0.0033707066176450473,Callithrix_pygmaea:0.007588491345612236):0.002525863246995108):0.0021712122202276474):0.007126053590139181):0.007599256865286119):0.00459802994146824):0.0018915280590326433):0.01316951344310563,((Aotus_vociferans:0.0052773887344409465,((Aotus_lemurinus:0.0035630676929625515,Aotus_griseimembra:0.002667470734673716):0.0016946206847454626,Aotus_trivirgatus:0.006957266249311367):0.0010262443070521154):8.661790595680952E-4,(Aotus_nancymaae:0.0045119684686033,(Aotus_nigriceps:0.0026744746323730806,(Aotus_infulatus:0.0015524769049508058,Aotus_azarae:8.546864102587648E-4):0.0020071032433091607):0.0059279197010082):0.0012380236819581336):0.02368175373097947):9.650825341235292E-4):0.0057499708386351545):0.0027207321674267426):0.0538699052391346,((((Pongo_abelii:0.005871569393071541,Pongo_pygmaeus:0.0062660254326162845):0.019104624532652215,(((Pan_troglodytes:0.0036595513022038006,Pan_paniscus:0.003845542355302156):0.007015377552644364,Homo_sapiens:0.012090269993845992):0.0028585801876852557,(Gorilla_beringei:3.869519049293224E-6,Gorilla_gorilla:0.0025582592589533104):0.010861066921390605):0.01109554659335199):0.005188988101317793,((Hoolock_hoolock:0.002349500039712127,Hoolock_leuconedys:0.002600283490434785):0.008387356827407988,((Symphalangus_syndactylus:0.009672883561655521,(Hylobates_pileatus:0.006995976620239097,(((Hylobates_klossii:0.006006377379431771,Hylobates_moloch:0.00583003501877144):0.0012043387699977592,((Hylobates_agilis:0.001963424872815922,Hylobates_albibarbis:0.0035793477730577394):0.003900206628589642,(Hylobates_abbotti:0.002131513510750138,(Hylobates_funereus:0.003189829888888829,Hylobates_muelleri:0.0017506725689093172):0.001129346831066913):0.0021742325781246574):5.83778386547662E-4):6.869868299325121E-4,Hylobates_lar:0.006102571901682452):0.0016710733073504458):0.005628950287456025):0.0013448792382221297,((((Nomascus_leucogenys:7.247248918615945E-4,Nomascus_siki:7.232141183606267E-4):0.001734026709973746,Nomascus_gabriellae:0.002562045708505767):0.0011778894632075154,Nomascus_concolor:0.0026783896873950885):0.0015282058414438637,(Nomascus_nasutus:0.006909127566541051,Nomascus_hainanus:0.007101678611999951):0.002994771625463355):0.00791709904725968):0.001855566686481236):0.01792636158743116):0.013390977295696549,((((Macaca_sylvanus:0.012529991967239862,((((Macaca_nemestrina:0.003483882283903783,Macaca_silenus:0.006114209797654924):9.750844343984766E-4,(Macaca_pagensis:0.008551528957442989,(Macaca_siberu:0.0011037291730289778,Macaca_leonina:0.0056178225258141645):0.001670775349125242):0.001718823561873195):0.002016208160793842,((Macaca_hecki:0.0045065104516931465,(Macaca_nigrescens:0.005092452857104468,(Macaca_tonkeana:0.004112503754761632,Macaca_nigra:0.004429585248092893):0.001379481011617445):9.456356212277361E-4):2.857636877991454E-4,(Macaca_maura:0.004981759951791398,(Macaca_ochreata:0.0023432795130695627,Macaca_brunnescens:0.004487641095083705):0.0018245267782537322):7.145752541963257E-4):0.0026093412758874668):0.0026292608304855913,((Macaca_arctoides:0.006102929291930348,(((Macaca_thibetana:0.00193198235137787,Macaca_assamensis:0.0018625711980988502):0.0036318274887746083,Macaca_sinica:0.006975395205422175):0.0014923297706183614,(Macaca_radiata:0.002991678516407248,Macaca_munzala:0.006509198937071581):0.0038463587562667656):0.0019450511960337757):0.0013929198407105492,((Macaca_cyclopis:0.0031779071795354774,(Macaca_mulatta:0.004535343883651566,Macaca_fuscata:0.0032995006937869276):6.851415864423949E-4):0.0021998254959119667,Macaca_fascicularis:0.009333187186615188):0.0011088412974876172):0.0024884729821543217):0.0023564605616066547):0.00777832675813267,((Theropithecus_gelada:0.008803912588199014,(((Papio_papio:0.00302010082674542,(Papio_hamadryas:0.0040289775026260966,((Papio_cynocephalus:0.00243930461251618,Papio_ursinus:0.005821688093039401):0.001524703910721903,Papio_anubis:0.001856363974978792):1.3313659915248355E-4):6.199198536165906E-4):0.001842217248740896,Rungwecebus_kipunji:0.0035871112406854477):0.004099167597372166,(Lophocebus_albigena:0.001847141200989666,Lophocebus_aterrimus:0.001028124861887858):0.005573682221913068):0.0016834036873945024):0.00689287546439149,((Mandrillus_leucophaeus:0.004340156023918185,Mandrillus_sphinx:0.003943227428003626):0.004135956250610584,((Cercocebus_chrysogaster:2.2235946786924865E-4,(Cercocebus_galeritus:0.0014793070768157057,Cercocebus_agilis:6.561164474875869E-4):0.0027694191276393387):0.004217088918434919,(Cercocebus_atys:0.005376375399673228,Cercocebus_torquatus:1.2572471721705725E-5):0.007551028241653712):0.002368513904617897):0.0056204310888089415):0.0025806815078538436):0.004920693894413852,((((Cercopithecus_campbelli:3.869519049237713E-6,(Chlorocebus_cynosuros:9.079855613016163E-4,(Chlorocebus_sabaeus:0.005929467555030654,(Chlorocebus_pygerythrus:0.004584445071991905,(Chlorocebus_aethiops:0.002819247844690731,Chlorocebus_tantalus:0.0036557512532698344):5.767513465886887E-4):0.002445523594719501):5.25724994967891E-4):0.005997554265807226):7.376296451643283E-4,((Cercopithecus_solatus:0.01231975321852502,(Cercopithecus_preussi:0.006125244248765815,Cercopithecus_lhoesti:0.002498952808381416):0.0029507104076019752):0.0031993291011226943,Erythrocebus_patas:0.011023784290810357):7.865428637906513E-4):0.0039182688378541775,(Cercopithecus_hamlyni:0.009944987669779415,((Cercopithecus_neglectus:0.01270222702717172,(Cercopithecus_mona:0.006582881896494197,(Cercopithecus_pogonias:0.0011435509429463808,Cercopithecus_wolfi:9.949053723664059E-4):0.0010448165620156047):0.0031399882666919288):0.001209780870375321,(Cercopithecus_diana:0.006799587911525795,((Cercopithecus_nictitans:0.01061919458303845,(Cercopithecus_albogularis:0.0011974394238851715,Cercopithecus_mitis:0.001645261367667683):0.0025519239785294356):0.002307800218896927,((Cercopithecus_ascanius:0.002900879619820773,(Cercopithecus_erythrogaster:0.004255086906780836,(Cercopithecus_erythrotis:0.0044565265834485834,Cercopithecus_cephus:0.001465436886523508):0.0011948827418241104):4.484434375872093E-4):0.0012935413428690201,Cercopithecus_petaurista:0.004030976289017385):0.0019050596366086414):0.0020760237821009775):6.105824674733085E-4):4.5178851870442127E-4):0.001957350451013029):0.0017503263872490349,(Allenopithecus_nigroviridis:0.012657865588590611,(Miopithecus_ogouensis:0.00544273927528266,Miopithecus_talapoin:0.006398939762038536):0.0032792739086844325):0.0016864259770761891):0.004695783236112194):0.009654229662423852,((((((Semnopithecus_priam:0.0021936386359580617,Trachypithecus_vetulus:0.0019110875376353587):0.0024202895994947515,(((Semnopithecus_hector:0.0027560450286160942,Semnopithecus_entellus:0.0031101549569221842):0.0027289423980024208,Trachypithecus_johnii:0.009204950169339698):0.002188927220737047,(Trachypithecus_geei:3.419723288441978E-4,Trachypithecus_pileatus:7.35737904457745E-4):0.004022973123470719):0.0013647404835023247):0.008462188277554183,((Trachypithecus_laotum:0.004671816753946123,(Trachypithecus_hatinhensis:7.534913360124906E-4,(Trachypithecus_delacouri:6.033700346589344E-4,(Trachypithecus_francoisi:0.001516312659929575,Trachypithecus_poliocephalus:0.0013793523458678347):8.916484233998379E-4):3.9830842231108887E-4):0.0015383347714710838):0.0018441726641111877,((Trachypithecus_auratus:9.946731292362343E-4,(Trachypithecus_cristatus:0.003036173059509928,Trachypithecus_germaini:0.002521858905091545):4.4900737201153396E-4):0.002597657124010555,((Trachypithecus_barbei:0.005872753440264056,Trachypithecus_obscurus:0.0036233826300458016):0.00293394470754077,Trachypithecus_phayrei:0.006281191780739659):8.625864078351531E-4):0.0010996395340244214):0.007124288587041505):0.005467286267019533,(((Rhinopithecus_bieti:0.0059943325614887355,(Rhinopithecus_roxellana:0.006693385098289761,Rhinopithecus_avunculus:0.008897899082055127):0.001534196762777218):0.0021700624776280764,Rhinopithecus_brelichi:0.002161855159366066):0.009179091571112463,((Simias_concolor:0.005064291857491521,Nasalis_larvatus:0.006638976956843512):0.00935164916940101,(Pygathrix_nigripes:0.00276334943165929,(Pygathrix_cinerea:0.0012589294967423892,Pygathrix_nemaeus:0.0011592692450635433):0.0027250844315565015):0.01335793486220771):0.0012363154594193793):0.0035178591419232075):9.194846838011528E-4,(((Presbytis_siberu:0.003698556967293487,Presbytis_potenziani:0.002249295873132051):0.002645037702241837,((Presbytis_sumatranu:0.005926852993033194,((Presbytis_bicolor:0.005251840387678508,Presbytis_rubicunda:0.0029520824567049186):4.8049296450908274E-4,Presbytis_melalophos:0.0018707672723592417):3.7775128599076835E-4):0.0011053334370476064,((Presbytis_fredericae:0.003069941243230845,Presbytis_comata:0.0010965379757539306):8.052346408947697E-4,Presbytis_mitrata:0.004571445617900127):3.976298818973989E-4):9.141951664046744E-4):0.0046580725366312525,((Presbytis_hosei:0.012491187082829647,(Presbytis_frontata:0.012088989935561845,Presbytis_chrysomelas:0.011161597177879234):0.002423619804178323):0.0025897019186332804,(Presbytis_thomasi:0.013819622690997402,Presbytis_femoralis:0.014993999646926659):0.0017259162657153326):8.143574553123312E-4):0.008290102879204175):0.005508760340737551,((((((Piliocolobus_foai:0.0031392016910024023,(Piliocolobus_tephrosceles:0.0026452710067492724,Piliocolobus_rufomitratus:0.0024992654067865216):7.017698830969477E-4):0.0029200961391700475,(Piliocolobus_kirkii:0.0022458492505791905,Piliocolobus_gordonorum:0.001994096188266181):0.0020287611965955077):0.005313813593863348,((Piliocolobus_pennantii:8.061061415775561E-4,Piliocolobus_preussi:8.899214563579116E-4):0.007010417244639799,Piliocolobus_tholloni:0.008562536676770593):0.001424659238338566):0.001445493625959915,Piliocolobus_badius:0.008723632967610695):0.008953524743312569,Procolobus_verus:0.01632118325582277):0.00279162419342599,(Colobus_satanas:0.013886895291097268,(((Colobus_polykomos:3.570707027031883E-4,Colobus_vellerosus:0.0012180643846014805):0.0030418860277134363,Colobus_guereza:0.003575567669213353):0.0030592875280603393,Colobus_angolensis:0.005797760996951562):0.003270277347911743):0.010119188016169622):0.005224353553571981):0.010950457416915582):0.02534065828843063):0.029786502265521153):0.08638902634283274):0.011730651011150561):0.016061875536599435):0.006476634757797731,(Lagomorpha:0.25809728600618537,(Tupaia_glis:0.026399863498853815,Tupaia_minor:0.028889856563217797):0.22657308498934436):0.006476634757797714);

6. RAxML phylogram based on ten mitochondrial genes.

((((Tarsius_bancanus:0.08132064318827636,Tarsius_syrichta:0.1174752348936271):0.060887167776852213,(Tarsius_sangirensis:0.0523646757115761,((Tarsius_lariang:0.015520079058190528,(Tarsius_wallacei:0.017357319937446047,Tarsius_tarsier:0.01569378725827364):0.004715513059267207):0.005795245786517689,Tarsius_dentatus:0.0074764482852567316):0.013455837038804042):0.07599188027079229):0.1342810425365346,(((Daubentonia_madagascariensis:0.248894561973939,(((Varecia_variegata:0.006617455888366153,Varecia_rubra:0.013382100114632145):0.10959676616500946,((Lemur_catta:0.055585505108654676,((Hapalemur_aureus:0.04159155341796705,((Hapalemur_alaotrensis:7.562001227990889E-4,(Hapalemur_meridionalis:0.0517579108050098,Hapalemur_griseus:6.369498248948169E-6):0.007846719915130973):0.006800776497938754,Hapalemur_occidentalis:0.010955881675963286):0.03849824607143948):0.021262957083124978,Prolemur_simus:0.06246316703604138):0.007345862679913995):0.03416284803110081,(Eulemur_coronatus:0.03273759307183821,((((Eulemur_cinereiceps:0.009365442139537161,Eulemur_collaris:0.006222786452008577):0.00896937145700294,((Eulemur_rufifrons:0.009610693287396055,((Eulemur_fulvus:0.004195186605447598,Eulemur_albifrons:0.003756597493191016):0.0026523955563280133,Eulemur_sanfordi:0.003517637156790654):0.0021317114103928114):0.0018485841579068962,Eulemur_rufus:0.009751771527555464):0.004818612162400315):0.014249920188677656,(Eulemur_mongoz:0.031925673263408116,Eulemur_rubriventer:0.027824418573829557):0.003958005018441901):0.013735430438051033,(Eulemur_macaco:0.010986447466366678,Eulemur_flavifrons:0.014986073847867543):0.019139476113664178):0.004965913213937911):0.0564768757877514):0.0273132478574607):0.04630848756645978,((Indri_indri:0.10657943152659799,(((((Avahi_ramanantsoavani:0.007112992057635936,Avahi_meridionalis:0.005829159884950252):0.004839221094849799,(Avahi_peyrierasi:0.0029938453106999052,Avahi_betsilio:0.004024720963414219):0.008094337272102958):0.007763169952560545,Avahi_laniger:0.015132325282354309):0.03876715637165751,(Avahi_unicolor:0.010056553087269271,(Avahi_cleesei:0.009655776725036702,Avahi_occidentalis:0.012994945152026038):0.0015459994751768624):0.03780446989408637):0.07662519605488749,((Propithecus_verreauxi:0.02578215027236419,(Propithecus_coquereli:0.004259304915142592,Propithecus_tattersalli:0.005408052716313394):0.021879728378328744):0.018879507575592314,(Propithecus_perrieri:0.0069744084436690335,(Propithecus_diadema:0.010045181769843037,Propithecus_edwardsi:0.00904591053129944):0.006131641101594587):0.02804913491177552):0.04453394984508707):0.019936354220537966):0.04108824380118903,(((((Lepilemur_aeeclis:0.045273062811090115,(Lepilemur_randrianasoloi:0.018038066142846976,(Lepilemur_ruficaudatus:0.010799509888998582,Lepilemur_hubbardorum:0.028731808187520302):0.00906044457023436):0.009407127699317508):0.021232742848857455,(Lepilemur_leucopus:0.004879223373383423,Lepilemur_petteri:0.009698116141303004):0.0503284144845112):0.013707800337795839,((((Lepilemur_ankaranensis:0.005389277951045046,(Lepilemur_tymerlachsoni:0.013820263125865151,Lepilemur_milanoii:0.00819594468930307):0.002320663129152467):0.00923058692042733,(Lepilemur_dorsalis:0.016306079247981997,(Lepilemur_mittermeieri:0.01869521093945481,(Lepilemur_sahamalazensis:0.012768052599515078,Lepilemur_ahmansoni:0.013939036580864261):0.005106148963170742):0.005470681638904629):0.0012260442843744501):0.023233238639554843,Lepilemur_septentrionalis:0.041783496352629124):0.014052783092092347,(Lepilemur_microdon:0.028607624131833265,(Lepilemur_otto:0.013269757113032354,(Lepilemur_edwardsi:0.005923778094199306,Lepilemur_grewcockorum:0.012303261016809786):0.001988347810119362):0.024473932677719135):0.0119328319058426):0.012507475043222027):0.04174280794398577,(Lepilemur_seali:0.061630552318921894,((Lepilemur_fleuretae:0.02086046581894574,((Lepilemur_jamesorum:0.01247790830453166,Lepilemur_betsileo:0.003651230595227206):0.004089986936390844,Lepilemur_mustelinus:0.02102825333546754):0.0070830244943413945):0.019427731937923798,Lepilemur_wrighti:0.03143898646872878):0.015502116170132196):0.036578958936372974):0.08528644475591252,(Phaner_pallescens:0.21958293522847983,((Cheirogaleus_sibreei:0.06346092188578112,((Cheirogaleus_medius:0.04289523773614279,Cheirogaleus_major:0.024768973712299247):0.02251860805337247,Cheirogaleus_crossleyi:0.055093181479229814):0.026888273980639865):0.03543931162062308,(Allocebus_trichotis:0.10717280120434569,((Mirza_zaza:3.9345649912414515E-4,Mirza_coquereli:0.004190335763025743):0.1021044229083673,((Microcebus_murinus:0.04425915898684263,Microcebus_griseorufus:0.041011509393864554):0.021125860419143216,((Microcebus_spDWW2010aMarolambo:0.018195093601949708,Microcebus_gerpi:0.014665206715302803):0.0270089704847859,((Microcebus_tavaratra:0.04463313453324169,((((Microcebus_berthae:0.01550058425563694,(Microcebus_myoxinus:0.021804105549232178,Microcebus_lehilahytsara:0.018898958980331004):6.766641154598574E-4):0.008528166530509362,Microcebus_mittermeieri:0.024981195188970684):0.0026277698678593975,(Microcebus_rufus:0.014655718809473584,((Microcebus_mamiratra:0.00828658625913381,Microcebus_margotmarshae:0.01020775142049557):0.014745855729911717,(Microcebus_sambiranensis:0.009676475472893853,Microcebus_arnholdi:0.007819620946328265):0.01727285246568988):0.0043073015271877235):0.00732739363048196):0.004225167361698534,((Microcebus_jollyae:1.890329318935402E-4,(Microcebus_sp_nova1Louis06:0.011339332902520205,Microcebus_sp_nova3Louis06:6.369498248948169E-6):0.005785518229564968):0.018547225744653506,(Microcebus_spDWW2010aManantantel:0.028961300698394532,Microcebus_simmonsi:0.02681860504987532):0.0027406209962271832):0.003928575775751897):0.004555451326061832):0.009625502367858974,((Microcebus_macarthurii:0.022205009868402015,Microcebus_sp_nova3Radespiel08:0.02383774651483017):0.015905088991862937,(Microcebus_danfossi:0.02375126034502395,(Microcebus_ravelobensis:0.01184187806801218,Microcebus_bongolavensis:0.003615464815093228):0.02222204120936322):0.010209870929537401):0.00917110597410431):0.008176684407634638):0.014479525317448672):0.06706914294731581):0.016635535302308657):0.04059319684967566):0.03404337191058049):0.010733841637645347):0.01846396630197822):0.015856973321868972):0.07200380650401728):0.03740639136129875,((Perodicticus_potto:0.1046201086569103,(Arctocebus_aureus:0.0017836696481207115,Arctocebus_calabarensis:0.002344707563191162):0.11627303391180166):0.05914310216705798,(((Nycticebus_pygmaeus:0.057981742968576166,(Nycticebus_menagensis:0.029696799742341184,(Nycticebus_javanicus:0.014280872614765738,(Nycticebus_bengalensis:4.6334160199124064E-4,Nycticebus_coucang:0.0018813515617142063):0.006353389838681056):0.011501604119285358):0.039694560104692855):0.09896226914284512,(Loris_tardigradus:6.369498248948169E-6,Loris_lydekkerianus:6.369498248948169E-6):0.1329115319959705):0.05799963909484962,(Galago_demidoff:0.10323320731702634,((((Galago_gallarum:0.01685952487280451,(Galago_senegalensis:0.006894274066438388,Galago_moholi:0.017100753279354675):0.011077001430826572):0.018818348952384656,(Euoticus_elegantulus:0.02970005574965473,Galago_matschiei:0.0213637470373369):0.03942504944782832):0.04725334944658288,(Galago_orinus:0.07552951134973662,(Galago_zanzibaricus:0.017779644388073557,Galago_granti:0.006764898948862674):0.03364704005399255):0.016890607213006947):0.012053630229705625,((Galago_gabonensis:6.369498248948169E-6,Galago_alleni:0.006024916170591088):0.05810666815271448,((Otolemur_crassicaudatus:0.004171448369556741,Otolemur_garnettii:0.0030141812368619236):0.05502446430756247,Otolemur_monteiri:0.01886362866459712):0.014961257205767131):0.021338351683183543):0.01799257357246531):0.07802606679026536):0.026236433319584362):0.12389103691795567):0.043029639356602256,((Cynocephalus_volans:0.12161554718051604,Galeopterus_variegatus:0.11762452474820884):0.21080203258081065,((Pithecia_pithecia:0.10478216949740643,((((Pithecia_irrorata:0.002903638100155259,Pithecia_monachus:0.0024066079256092054):0.0778950542782651,((Cacajao_calvus:0.018246442186105827,(Cacajao_melanocephalus:0.009913722655121249,(Cacajao_ayresi:0.0012780763349432567,Cacajao_hosomi:0.002020767983359484):0.009934002346111082):0.017400018625649882):0.02816008577443474,(Chiropotes_utahicki:0.006257250005161308,(Chiropotes_chiropotes:0.003894331447441002,Chiropotes_israelita:0.0034523693792241072):0.009390609784475257):0.05676665483300514):0.03747351855444053):0.05548410828359851,((Callicebus_torquatus:0.023893001035400374,Callicebus_lugens:0.0073273779694524865):0.03730696952523893,(Callicebus_personatus:0.06739356068288321,((Callicebus_moloch:0.013615111473279429,Callicebus_hoffmannsi:0.027169384989710332):0.01960711730091036,Callicebus_donacophilus:0.029766809386776116):0.02932773658298965):0.016426622691644577):0.05321999579225828):0.015617559671936954,((((Brachyteles_hypoxanthus:0.08958311990204793,(Ateles_paniscus:0.009867125735373627,(((Ateles_chamek:0.0014173397566729085,Ateles_marginatus:0.01476751135838672):0.004073504830509167,Ateles_belzebuth:0.008556295685399395):0.005906857004344235,(Ateles_geoffroyi:0.017645178289304897,Ateles_fusciceps:0.009041277536314118):0.008951279230466902):0.005350562635254019):0.03322517436300154):0.008984961360253396,(Lagothrix_lugens:0.004047447066794274,(Lagothrix_cana:0.007561028320275387,(Lagothrix_poeppigii:0.010340848771843936,(Brachyteles_arachnoides:0.016437158818116693,Lagothrix_lagotricha:0.003994644484497045):0.004147330691256057):7.700956888742061E-4):0.00879790860773999):0.05792182704315618):0.029752350625061652,(((((Alouatta_nigerrima:0.0035635466233571167,(Alouatta_macconnelli:6.369498248948169E-6,Alouatta_seniculus:6.369498248948169E-6):0.0024567975339239156):0.011809966196472399,Alouatta_sara:0.011034766633600313):0.007374661657510551,Alouatta_caraya:0.016255406236028558):0.007055981617000273,(Alouatta_guariba:0.01779233071170888,Alouatta_belzebul:0.02152050105074338):0.0038253877041530826):0.008604639965975769,(Alouatta_pigra:0.018272412781228653,(Alouatta_palliata:0.0029015684052302038,Alouatta_coibensis:0.002687319553476064):0.012019522088156154):0.011702145020304366):0.03797966385134777):0.022672257773649396,(((Saimiri_ustus:0.013038408129460999,(Saimiri_boliviensis:0.021720374763801953,(Saimiri_oerstedii:0.012488893054869776,Saimiri_sciureus:0.013309904257174154):0.002464141102518136):0.003936447619691363):0.14250756191615616,(((Cebus_albifrons:0.003071780274143898,Cebus_olivaceus:0.007973949223622157):0.0075527357354661095,Cebus_capucinus:0.020257476554035647):0.03520381041736831,(Cebus_xanthosternos:0.03186382254579567,(Cebus_libidinosus:0.002337927220425695,Cebus_apella:0.00489784701578444):0.017967299852303165):0.0239732680773177):0.07975801898362683):0.01749329581448833,((((Aotus_lemurinus:0.014803417705891775,Aotus_griseimembra:0.012319565172160707):0.0071939173191059025,(Aotus_nancymaae:0.024559415802950513,Aotus_vociferans:0.020208818802233353):0.0048926053547648785):0.004721261492998674,(((Aotus_infulatus:0.0038043299975015765,Aotus_azarae:0.0036929046111665587):0.007593886644931436,Aotus_nigriceps:0.011655053304589602):0.01835094951116445,Aotus_trivirgatus:0.029774616015610822):0.0040338674069029246):0.07877391392166733,((((Saguinus_graellsi:0.0034079514183710247,Saguinus_nigricollis:0.0011428786064250351):0.01719863875890848,((Saguinus_fuscicollis:0.017449338135457904,Saguinus_tripartitus:0.02110019903994942):0.004006927316015041,Saguinus_melanoleucus:0.015211281481340433):0.004893026797277211):0.03820926720342932,(((Saguinus_niger:0.004111013619643611,Saguinus_midas:0.020609187058794443):0.04818021020729579,(Saguinus_oedipus:0.013009417951988178,Saguinus_geoffroyi:0.0063270782229883915):0.04897075158344544):0.012533766657306655,((Saguinus_leucopus:0.057650963658039234,(Saguinus_imperator:0.04508934700886946,(Saguinus_mystax:0.021099943785852004,Saguinus_labiatus:0.013114514482946316):0.011186374603427529):0.005769612720016193):0.0054675393437618736,(Saguinus_bicolor:6.369498248948169E-6,Saguinus_martinsi:0.010282375410206157):0.040645744191428856):0.029870185137637062):0.019507051613231408):0.0474337229422474,((Leontopithecus_chrysomelas:0.0045636189859459275,(Leontopithecus_rosalia:6.369498248948169E-6,Leontopithecus_chrysopygus:6.369498248948169E-6):0.01188369415337398):0.1252398684971237,(Callimico_goeldii:0.11621941767259136,((Callithrix_pygmaea:0.036600637388743085,(Callithrix_humilis:0.011479441826232173,((Callithrix_emiliae:6.369498248948169E-6,(Callithrix_humeralifera:0.012729933024571372,Callithrix_mauesi:0.003040151647277889):0.002387535031042942):0.00763957328016851,Callithrix_argentata:0.017446764121957004):0.014911338573186717):0.015687239545413845):0.008890714338683048,((((Callithrix_kuhlii:0.023186510496160717,(Callithrix_jacchus:0.0010490484214535911,Callithrix_penicillata:0.0047728449884133495):0.003451818534186968):0.002587444919624371,Callithrix_geoffroyi:0.009876427368449736):0.02478980289431465,Callithrix_aurita:0.03419335006366464):0.020686980396667054,Callithrix_saterei:0.07707864905854989):6.369498248948169E-6):0.03662994726905733):0.012501078016115386):0.007389494612822789):0.030752642233332572):0.005269820006978898):0.010999330155711862):0.010714734360427858):0.015869011480582673):0.15365434152764285,(((((Colobus_satanas:0.05325344542865873,(((Colobus_polykomos:0.0014728844402403518,Colobus_vellerosus:0.004902647603770127):0.012692341559297382,Colobus_guereza:0.012200630075102692):0.017449437865276307,Colobus_angolensis:0.028971399932789743):0.016035225438602696):0.04985075387708915,(Procolobus_verus:0.059681032121898014,(Piliocolobus_badius:0.03944682051677956,(((Piliocolobus_preussi:0.003648081911249601,Piliocolobus_pennantii:0.003246694998318367):0.028244462895916533,Piliocolobus_tholloni:0.03433652974804008):0.005897740488391312,((Piliocolobus_foai:0.012719734659882243,(Piliocolobus_rufomitratus:0.010152626911311469,Piliocolobus_tephrosceles:0.010718611212225004):0.002834680395132405):0.01180544143582285,(Piliocolobus_kirkii:0.009095191087242493,Piliocolobus_gordonorum:0.00809920053384905):0.00825633300943962):0.021156953948534563):0.00445656979471476):0.04038493592424519):0.012623651632272415):0.022929137551406575,(((((Trachypithecus_geei:0.0014230804331937796,Trachypithecus_pileatus:0.0030818982317873944):0.01537589238260828,(Semnopithecus_priam:0.007081134045942961,Trachypithecus_vetulus:0.01574922629856218):0.026583275501890546):0.00617356036349781,((Semnopithecus_entellus:0.014512616262176536,Semnopithecus_hector:0.013429314882362375):0.019181018770213942,Trachypithecus_johnii:0.03447361330277249):0.010923361504891038):0.061340312134155806,((Simias_concolor:0.013313350972162352,Nasalis_larvatus:0.03444644664902463):0.04205847212187508,((Pygathrix_nemaeus:0.0025399818113008843,Pygathrix_cinerea:0.0039456312708916474):0.0855481663638138,(Rhinopithecus_bieti:0.02251401938908476,((Rhinopithecus_brelichi:0.018366353498734,Rhinopithecus_avunculus:0.03580120169119194):0.006037634805500236,Rhinopithecus_roxellana:0.02759729933986832):0.004154227252931575):0.04335699887028843):0.010066549339814657):0.017195776302707078):0.0058151698727408885,(((Trachypithecus_phayrei:0.03536743230210759,((Trachypithecus_francoisi:0.009032399922331236,Trachypithecus_poliocephalus:0.004752736794745138):0.018948264213112576,Trachypithecus_laotum:0.016675510074480704):0.009804299424135299):0.00834828756581052,((Trachypithecus_germaini:0.009702050941237272,(Trachypithecus_auratus:0.0102433235445612,Trachypithecus_cristatus:0.01219796203968454):5.372012949833582E-4):0.01790067631788883,(Trachypithecus_obscurus:0.0170409852292841,Trachypithecus_barbei:0.02147372267837161):0.0086355699669447):0.009547550713999597):0.04519548321737776,(Presbytis_thomasi:0.05370873081113686,(((Presbytis_hosei:0.049396839441937124,(Presbytis_frontata:0.04772929227694189,Presbytis_chrysomelas:0.046787201277576074):0.01183536994247747):0.008474766073830686,((Presbytis_siberu:0.015599898144765367,Presbytis_potenziani:0.00914101776805254):0.008867021025656785,(((Presbytis_melalophos:0.013676398055127437,(Presbytis_bicolor:0.021849178347959353,Presbytis_rubicunda:0.012260090214561592):0.0016951908312316522):0.0026472710538874566,Presbytis_sumatranu:0.02428723090791557):0.008730675647731612,(Presbytis_mitrata:0.018480881515662406,(Presbytis_fredericae:0.011885057070940341,Presbytis_comata:0.010676967898164691):0.007631912142309405):0.0037450788781832056):0.007047355696521096):0.03720335544966924):0.00717602817792895,Presbytis_femoralis:0.044848661288532155):0.00548132177035332):0.04687713275563554):0.012473389705932925):0.026187786046941608):0.05562386465620239,((((Theropithecus_gelada:0.04558595026569634,((((Papio_ursinus:0.023921015879089458,Papio_cynocephalus:0.010434554819230724):0.006062657757107015,(Papio_anubis:0.012086044559357578,Papio_papio:0.019988074322456528):0.0015864158473404277):0.0017745352136731007,Papio_hamadryas:0.019144420236709703):0.011602694782106071,Rungwecebus_kipunji:0.01375235332414726):0.02454281650540413):0.009344555555175327,(Lophocebus_aterrimus:0.014941365497973869,Lophocebus_albigena:0.027339719552785025):0.030699495290422485):0.04038142291639091,((Miopithecus_talapoin:0.04397824364413472,Miopithecus_ogouensis:0.04327767956968409):0.040620228955065785,(((Mandrillus_sphinx:0.027446792734775682,Cercocebus_torquatus:0.04138412752111148):0.015185334927495742,(Mandrillus_leucophaeus:0.017195751043389773,(Cercocebus_agilis:7.092580193048814E-4,Cercocebus_galeritus:0.008077672918737067):0.034425656567993235):0.011770291252231946):0.051118853236231,(Macaca_sylvanus:0.060222513869465866,((((((Macaca_tonkeana:0.024518186067123193,Macaca_nigra:0.02087341039356455):0.012095084413470292,Macaca_nigrescens:0.020039638155084694):0.00907190406315661,Macaca_hecki:0.017579344791795348):0.0029970891533526167,(Macaca_maura:0.03420456001766592,(Macaca_brunnescens:0.018679050630638516,Macaca_ochreata:0.016131624792055044):0.011627005112752054):0.004704372915675492):0.013864931250007761,(Macaca_silenus:0.03916847026876402,(Macaca_pagensis:0.03538042510473999,(Macaca_nemestrina:0.014997349383625513,(Macaca_leonina:0.019486836881571656,Macaca_siberu:0.011507962042011655):0.013834617495545931):0.007205072740227747):0.007738640793824358):0.008210579672638385):0.012696720015731855,(((Macaca_sinica:0.03583739839825384,(Macaca_thibetana:0.009521154913597618,Macaca_assamensis:0.007138215345214638):0.020638672520795653):0.008198065085477602,(Macaca_radiata:0.016142748921443184,Macaca_munzala:0.025117935773349365):0.015617871469517652):0.008478049359333828,((Macaca_mulatta:0.024580718258271295,(Macaca_fuscata:0.0174601731219155,Macaca_cyclopis:0.00920388370587577):0.007673944360460094):0.012557359012168812,(Macaca_fascicularis:0.041396043051151854,Macaca_arctoides:0.03468454926401876):0.005994644002364824):0.008437299086751282):0.014230039579533282):0.012023970825667973):0.035286348459816486):0.005069371593433014):0.004871206906273184):0.01490142593097088,(Allenopithecus_nigroviridis:0.07420699886009707,(Erythrocebus_patas:0.10509133116109631,(((Cercopithecus_neglectus:0.09145044031381466,((Cercopithecus_diana:0.046965091039157913,(Cercopithecus_wolfi:0.0093745224920746,Cercopithecus_pogonias:6.369498248948169E-6):0.004860181204904834):0.003023261761758378,Cercopithecus_mona:0.037361986270014524):0.00825440288385293):0.01641105940387555,(Cercopithecus_campbelli:6.3694982488371465E-6,(Cercopithecus_hamlyni:0.08784739616384385,((((Chlorocebus_aethiops:0.013756255349056667,Chlorocebus_tantalus:0.014887486297977426):0.0032328350445458565,Chlorocebus_pygerythrus:0.017815790611892424):0.012816729052840592,Chlorocebus_sabaeus:0.02865383852475878):0.005707252727142564,Cercopithecus_solatus:0.03751394241977868):0.021223938770653206):0.010029664338211397):0.004192566893078453):0.004197162973344826,(((Cercopithecus_lhoesti:0.030701583743591554,Cercopithecus_preussi:0.02955459005327088):0.014848684162653392,((Cercopithecus_erythrotis:0.017282361403120894,Cercopithecus_cephus:0.011632275807630155):0.008504216719005475,((Cercopithecus_petaurista:0.024016364054107397,Cercopithecus_erythrogaster:0.009478598817466266):0.008902805490864862,Cercopithecus_ascanius:0.007434917382241446):0.005642611255636032):0.011483819844366083):0.033182580240389115,(Cercopithecus_mitis:0.03756310576236477,Cercopithecus_nictitans:0.028221561315006638):0.03224688987987834):0.013115307681064836):0.007793561908358004):0.016611873860871262):0.014999920513611764):0.04069093327031714):0.06855944378952816,((((Hoolock_leuconedys:0.010399326699089939,Hoolock_hoolock:0.010124832695397101):0.0398134264031591,((Hylobates_pileatus:0.027866419495685912,(((Hylobates_agilis:0.00899026904098732,Hylobates_albibarbis:0.014731716546166118):0.018826845584192675,((Hylobates_abbotti:0.008617678858274913,(Hylobates_funereus:0.01337309381694718,Hylobates_muelleri:0.008419260625478486):0.005422237088256288):0.009881984520503151,Hylobates_lar:0.027384800654991492):0.0030157773303041147):0.003032906421567172,(Hylobates_klossii:0.024087726742104576,Hylobates_moloch:0.02396631716003328):0.003784704315734966):0.008114014645180023):0.02238500507402963,Symphalangus_syndactylus:0.042237533071624034):0.003900950557881866):0.005539139183679875,(((Nomascus_gabriellae:0.012411381065280613,(Nomascus_leucogenys:0.0029584794956563076,Nomascus_siki:0.0030585517067944767):0.007668186666047072):0.00704681181645328,Nomascus_concolor:0.01249973209942512):0.00696371096673154,(Nomascus_nasutus:0.028529024461034003,Nomascus_hainanus:0.02913463014428852):0.012000259156448534):0.03260157742653291):0.06554473667712823,((Gorilla_gorilla:0.05915646833265098,(Homo_sapiens:0.05073373019186633,(Pan_paniscus:0.016063259587431822,Pan_troglodytes:0.01847105134745164):0.026630394104677935):0.014751907353944826):0.03337827900407708,(Pongo_abelii:0.0286228281867017,Pongo_pygmaeus:0.03457865346329858):0.07164526862167064):0.02748831723133982):0.04084720987745283):0.09470750665435901):0.137800830304458):0.03255285079645037):0.03298751213151424):0.027936669114151793,((Tupaia_minor:0.09832297797335532,Tupaia_glis:0.07507712125406552):0.2865604364444155,Lagomorpha:0.2504556359473566):0.0279366691141518);((((Tarsius_bancanus:0.08132064318827636,Tarsius_syrichta:0.1174752348936271):0.060887167776852213,(Tarsius_sangirensis:0.0523646757115761,((Tarsius_lariang:0.015520079058190528,(Tarsius_wallacei:0.017357319937446047,Tarsius_tarsier:0.01569378725827364):0.004715513059267207):0.005795245786517689,Tarsius_dentatus:0.0074764482852567316):0.013455837038804042):0.07599188027079229):0.1342810425365346,(((Daubentonia_madagascariensis:0.248894561973939,(((Varecia_variegata:0.006617455888366153,Varecia_rubra:0.013382100114632145):0.10959676616500946,((Lemur_catta:0.055585505108654676,((Hapalemur_aureus:0.04159155341796705,((Hapalemur_alaotrensis:7.562001227990889E-4,(Hapalemur_meridionalis:0.0517579108050098,Hapalemur_griseus:6.369498248948169E-6):0.007846719915130973):0.006800776497938754,Hapalemur_occidentalis:0.010955881675963286):0.03849824607143948):0.021262957083124978,Prolemur_simus:0.06246316703604138):0.007345862679913995):0.03416284803110081,(Eulemur_coronatus:0.03273759307183821,((((Eulemur_cinereiceps:0.009365442139537161,Eulemur_collaris:0.006222786452008577):0.00896937145700294,((Eulemur_rufifrons:0.009610693287396055,((Eulemur_fulvus:0.004195186605447598,Eulemur_albifrons:0.003756597493191016):0.0026523955563280133,Eulemur_sanfordi:0.003517637156790654):0.0021317114103928114):0.0018485841579068962,Eulemur_rufus:0.009751771527555464):0.004818612162400315):0.014249920188677656,(Eulemur_mongoz:0.031925673263408116,Eulemur_rubriventer:0.027824418573829557):0.003958005018441901):0.013735430438051033,(Eulemur_macaco:0.010986447466366678,Eulemur_flavifrons:0.014986073847867543):0.019139476113664178):0.004965913213937911):0.0564768757877514):0.0273132478574607):0.04630848756645978,((Indri_indri:0.10657943152659799,(((((Avahi_ramanantsoavani:0.007112992057635936,Avahi_meridionalis:0.005829159884950252):0.004839221094849799,(Avahi_peyrierasi:0.0029938453106999052,Avahi_betsilio:0.004024720963414219):0.008094337272102958):0.007763169952560545,Avahi_laniger:0.015132325282354309):0.03876715637165751,(Avahi_unicolor:0.010056553087269271,(Avahi_cleesei:0.009655776725036702,Avahi_occidentalis:0.012994945152026038):0.0015459994751768624):0.03780446989408637):0.07662519605488749,((Propithecus_verreauxi:0.02578215027236419,(Propithecus_coquereli:0.004259304915142592,Propithecus_tattersalli:0.005408052716313394):0.021879728378328744):0.018879507575592314,(Propithecus_perrieri:0.0069744084436690335,(Propithecus_diadema:0.010045181769843037,Propithecus_edwardsi:0.00904591053129944):0.006131641101594587):0.02804913491177552):0.04453394984508707):0.019936354220537966):0.04108824380118903,(((((Lepilemur_aeeclis:0.045273062811090115,(Lepilemur_randrianasoloi:0.018038066142846976,(Lepilemur_ruficaudatus:0.010799509888998582,Lepilemur_hubbardorum:0.028731808187520302):0.00906044457023436):0.009407127699317508):0.021232742848857455,(Lepilemur_leucopus:0.004879223373383423,Lepilemur_petteri:0.009698116141303004):0.0503284144845112):0.013707800337795839,((((Lepilemur_ankaranensis:0.005389277951045046,(Lepilemur_tymerlachsoni:0.013820263125865151,Lepilemur_milanoii:0.00819594468930307):0.002320663129152467):0.00923058692042733,(Lepilemur_dorsalis:0.016306079247981997,(Lepilemur_mittermeieri:0.01869521093945481,(Lepilemur_sahamalazensis:0.012768052599515078,Lepilemur_ahmansoni:0.013939036580864261):0.005106148963170742):0.005470681638904629):0.0012260442843744501):0.023233238639554843,Lepilemur_septentrionalis:0.041783496352629124):0.014052783092092347,(Lepilemur_microdon:0.028607624131833265,(Lepilemur_otto:0.013269757113032354,(Lepilemur_edwardsi:0.005923778094199306,Lepilemur_grewcockorum:0.012303261016809786):0.001988347810119362):0.024473932677719135):0.0119328319058426):0.012507475043222027):0.04174280794398577,(Lepilemur_seali:0.061630552318921894,((Lepilemur_fleuretae:0.02086046581894574,((Lepilemur_jamesorum:0.01247790830453166,Lepilemur_betsileo:0.003651230595227206):0.004089986936390844,Lepilemur_mustelinus:0.02102825333546754):0.0070830244943413945):0.019427731937923798,Lepilemur_wrighti:0.03143898646872878):0.015502116170132196):0.036578958936372974):0.08528644475591252,(Phaner_pallescens:0.21958293522847983,((Cheirogaleus_sibreei:0.06346092188578112,((Cheirogaleus_medius:0.04289523773614279,Cheirogaleus_major:0.024768973712299247):0.02251860805337247,Cheirogaleus_crossleyi:0.055093181479229814):0.026888273980639865):0.03543931162062308,(Allocebus_trichotis:0.10717280120434569,((Mirza_zaza:3.9345649912414515E-4,Mirza_coquereli:0.004190335763025743):0.1021044229083673,((Microcebus_murinus:0.04425915898684263,Microcebus_griseorufus:0.041011509393864554):0.021125860419143216,((Microcebus_spDWW2010aMarolambo:0.018195093601949708,Microcebus_gerpi:0.014665206715302803):0.0270089704847859,((Microcebus_tavaratra:0.04463313453324169,((((Microcebus_berthae:0.01550058425563694,(Microcebus_myoxinus:0.021804105549232178,Microcebus_lehilahytsara:0.018898958980331004):6.766641154598574E-4):0.008528166530509362,Microcebus_mittermeieri:0.024981195188970684):0.0026277698678593975,(Microcebus_rufus:0.014655718809473584,((Microcebus_mamiratra:0.00828658625913381,Microcebus_margotmarshae:0.01020775142049557):0.014745855729911717,(Microcebus_sambiranensis:0.009676475472893853,Microcebus_arnholdi:0.007819620946328265):0.01727285246568988):0.0043073015271877235):0.00732739363048196):0.004225167361698534,((Microcebus_jollyae:1.890329318935402E-4,(Microcebus_sp_nova1Louis06:0.011339332902520205,Microcebus_sp_nova3Louis06:6.369498248948169E-6):0.005785518229564968):0.018547225744653506,(Microcebus_spDWW2010aManantantel:0.028961300698394532,Microcebus_simmonsi:0.02681860504987532):0.0027406209962271832):0.003928575775751897):0.004555451326061832):0.009625502367858974,((Microcebus_macarthurii:0.022205009868402015,Microcebus_sp_nova3Radespiel08:0.02383774651483017):0.015905088991862937,(Microcebus_danfossi:0.02375126034502395,(Microcebus_ravelobensis:0.01184187806801218,Microcebus_bongolavensis:0.003615464815093228):0.02222204120936322):0.010209870929537401):0.00917110597410431):0.008176684407634638):0.014479525317448672):0.06706914294731581):0.016635535302308657):0.04059319684967566):0.03404337191058049):0.010733841637645347):0.01846396630197822):0.015856973321868972):0.07200380650401728):0.03740639136129875,((Perodicticus_potto:0.1046201086569103,(Arctocebus_aureus:0.0017836696481207115,Arctocebus_calabarensis:0.002344707563191162):0.11627303391180166):0.05914310216705798,(((Nycticebus_pygmaeus:0.057981742968576166,(Nycticebus_menagensis:0.029696799742341184,(Nycticebus_javanicus:0.014280872614765738,(Nycticebus_bengalensis:4.6334160199124064E-4,Nycticebus_coucang:0.0018813515617142063):0.006353389838681056):0.011501604119285358):0.039694560104692855):0.09896226914284512,(Loris_tardigradus:6.369498248948169E-6,Loris_lydekkerianus:6.369498248948169E-6):0.1329115319959705):0.05799963909484962,(Galago_demidoff:0.10323320731702634,((((Galago_gallarum:0.01685952487280451,(Galago_senegalensis:0.006894274066438388,Galago_moholi:0.017100753279354675):0.011077001430826572):0.018818348952384656,(Euoticus_elegantulus:0.02970005574965473,Galago_matschiei:0.0213637470373369):0.03942504944782832):0.04725334944658288,(Galago_orinus:0.07552951134973662,(Galago_zanzibaricus:0.017779644388073557,Galago_granti:0.006764898948862674):0.03364704005399255):0.016890607213006947):0.012053630229705625,((Galago_gabonensis:6.369498248948169E-6,Galago_alleni:0.006024916170591088):0.05810666815271448,((Otolemur_crassicaudatus:0.004171448369556741,Otolemur_garnettii:0.0030141812368619236):0.05502446430756247,Otolemur_monteiri:0.01886362866459712):0.014961257205767131):0.021338351683183543):0.01799257357246531):0.07802606679026536):0.026236433319584362):0.12389103691795567):0.043029639356602256,((Cynocephalus_volans:0.12161554718051604,Galeopterus_variegatus:0.11762452474820884):0.21080203258081065,((Pithecia_pithecia:0.10478216949740643,((((Pithecia_irrorata:0.002903638100155259,Pithecia_monachus:0.0024066079256092054):0.0778950542782651,((Cacajao_calvus:0.018246442186105827,(Cacajao_melanocephalus:0.009913722655121249,(Cacajao_ayresi:0.0012780763349432567,Cacajao_hosomi:0.002020767983359484):0.009934002346111082):0.017400018625649882):0.02816008577443474,(Chiropotes_utahicki:0.006257250005161308,(Chiropotes_chiropotes:0.003894331447441002,Chiropotes_israelita:0.0034523693792241072):0.009390609784475257):0.05676665483300514):0.03747351855444053):0.05548410828359851,((Callicebus_torquatus:0.023893001035400374,Callicebus_lugens:0.0073273779694524865):0.03730696952523893,(Callicebus_personatus:0.06739356068288321,((Callicebus_moloch:0.013615111473279429,Callicebus_hoffmannsi:0.027169384989710332):0.01960711730091036,Callicebus_donacophilus:0.029766809386776116):0.02932773658298965):0.016426622691644577):0.05321999579225828):0.015617559671936954,((((Brachyteles_hypoxanthus:0.08958311990204793,(Ateles_paniscus:0.009867125735373627,(((Ateles_chamek:0.0014173397566729085,Ateles_marginatus:0.01476751135838672):0.004073504830509167,Ateles_belzebuth:0.008556295685399395):0.005906857004344235,(Ateles_geoffroyi:0.017645178289304897,Ateles_fusciceps:0.009041277536314118):0.008951279230466902):0.005350562635254019):0.03322517436300154):0.008984961360253396,(Lagothrix_lugens:0.004047447066794274,(Lagothrix_cana:0.007561028320275387,(Lagothrix_poeppigii:0.010340848771843936,(Brachyteles_arachnoides:0.016437158818116693,Lagothrix_lagotricha:0.003994644484497045):0.004147330691256057):7.700956888742061E-4):0.00879790860773999):0.05792182704315618):0.029752350625061652,(((((Alouatta_nigerrima:0.0035635466233571167,(Alouatta_macconnelli:6.369498248948169E-6,Alouatta_seniculus:6.369498248948169E-6):0.0024567975339239156):0.011809966196472399,Alouatta_sara:0.011034766633600313):0.007374661657510551,Alouatta_caraya:0.016255406236028558):0.007055981617000273,(Alouatta_guariba:0.01779233071170888,Alouatta_belzebul:0.02152050105074338):0.0038253877041530826):0.008604639965975769,(Alouatta_pigra:0.018272412781228653,(Alouatta_palliata:0.0029015684052302038,Alouatta_coibensis:0.002687319553476064):0.012019522088156154):0.011702145020304366):0.03797966385134777):0.022672257773649396,(((Saimiri_ustus:0.013038408129460999,(Saimiri_boliviensis:0.021720374763801953,(Saimiri_oerstedii:0.012488893054869776,Saimiri_sciureus:0.013309904257174154):0.002464141102518136):0.003936447619691363):0.14250756191615616,(((Cebus_albifrons:0.003071780274143898,Cebus_olivaceus:0.007973949223622157):0.0075527357354661095,Cebus_capucinus:0.020257476554035647):0.03520381041736831,(Cebus_xanthosternos:0.03186382254579567,(Cebus_libidinosus:0.002337927220425695,Cebus_apella:0.00489784701578444):0.017967299852303165):0.0239732680773177):0.07975801898362683):0.01749329581448833,((((Aotus_lemurinus:0.014803417705891775,Aotus_griseimembra:0.012319565172160707):0.0071939173191059025,(Aotus_nancymaae:0.024559415802950513,Aotus_vociferans:0.020208818802233353):0.0048926053547648785):0.004721261492998674,(((Aotus_infulatus:0.0038043299975015765,Aotus_azarae:0.0036929046111665587):0.007593886644931436,Aotus_nigriceps:0.011655053304589602):0.01835094951116445,Aotus_trivirgatus:0.029774616015610822):0.0040338674069029246):0.07877391392166733,((((Saguinus_graellsi:0.0034079514183710247,Saguinus_nigricollis:0.0011428786064250351):0.01719863875890848,((Saguinus_fuscicollis:0.017449338135457904,Saguinus_tripartitus:0.02110019903994942):0.004006927316015041,Saguinus_melanoleucus:0.015211281481340433):0.004893026797277211):0.03820926720342932,(((Saguinus_niger:0.004111013619643611,Saguinus_midas:0.020609187058794443):0.04818021020729579,(Saguinus_oedipus:0.013009417951988178,Saguinus_geoffroyi:0.0063270782229883915):0.04897075158344544):0.012533766657306655,((Saguinus_leucopus:0.057650963658039234,(Saguinus_imperator:0.04508934700886946,(Saguinus_mystax:0.021099943785852004,Saguinus_labiatus:0.013114514482946316):0.011186374603427529):0.005769612720016193):0.0054675393437618736,(Saguinus_bicolor:6.369498248948169E-6,Saguinus_martinsi:0.010282375410206157):0.040645744191428856):0.029870185137637062):0.019507051613231408):0.0474337229422474,((Leontopithecus_chrysomelas:0.0045636189859459275,(Leontopithecus_rosalia:6.369498248948169E-6,Leontopithecus_chrysopygus:6.369498248948169E-6):0.01188369415337398):0.1252398684971237,(Callimico_goeldii:0.11621941767259136,((Callithrix_pygmaea:0.036600637388743085,(Callithrix_humilis:0.011479441826232173,((Callithrix_emiliae:6.369498248948169E-6,(Callithrix_humeralifera:0.012729933024571372,Callithrix_mauesi:0.003040151647277889):0.002387535031042942):0.00763957328016851,Callithrix_argentata:0.017446764121957004):0.014911338573186717):0.015687239545413845):0.008890714338683048,((((Callithrix_kuhlii:0.023186510496160717,(Callithrix_jacchus:0.0010490484214535911,Callithrix_penicillata:0.0047728449884133495):0.003451818534186968):0.002587444919624371,Callithrix_geoffroyi:0.009876427368449736):0.02478980289431465,Callithrix_aurita:0.03419335006366464):0.020686980396667054,Callithrix_saterei:0.07707864905854989):6.369498248948169E-6):0.03662994726905733):0.012501078016115386):0.007389494612822789):0.030752642233332572):0.005269820006978898):0.010999330155711862):0.010714734360427858):0.015869011480582673):0.15365434152764285,(((((Colobus_satanas:0.05325344542865873,(((Colobus_polykomos:0.0014728844402403518,Colobus_vellerosus:0.004902647603770127):0.012692341559297382,Colobus_guereza:0.012200630075102692):0.017449437865276307,Colobus_angolensis:0.028971399932789743):0.016035225438602696):0.04985075387708915,(Procolobus_verus:0.059681032121898014,(Piliocolobus_badius:0.03944682051677956,(((Piliocolobus_preussi:0.003648081911249601,Piliocolobus_pennantii:0.003246694998318367):0.028244462895916533,Piliocolobus_tholloni:0.03433652974804008):0.005897740488391312,((Piliocolobus_foai:0.012719734659882243,(Piliocolobus_rufomitratus:0.010152626911311469,Piliocolobus_tephrosceles:0.010718611212225004):0.002834680395132405):0.01180544143582285,(Piliocolobus_kirkii:0.009095191087242493,Piliocolobus_gordonorum:0.00809920053384905):0.00825633300943962):0.021156953948534563):0.00445656979471476):0.04038493592424519):0.012623651632272415):0.022929137551406575,(((((Trachypithecus_geei:0.0014230804331937796,Trachypithecus_pileatus:0.0030818982317873944):0.01537589238260828,(Semnopithecus_priam:0.007081134045942961,Trachypithecus_vetulus:0.01574922629856218):0.026583275501890546):0.00617356036349781,((Semnopithecus_entellus:0.014512616262176536,Semnopithecus_hector:0.013429314882362375):0.019181018770213942,Trachypithecus_johnii:0.03447361330277249):0.010923361504891038):0.061340312134155806,((Simias_concolor:0.013313350972162352,Nasalis_larvatus:0.03444644664902463):0.04205847212187508,((Pygathrix_nemaeus:0.0025399818113008843,Pygathrix_cinerea:0.0039456312708916474):0.0855481663638138,(Rhinopithecus_bieti:0.02251401938908476,((Rhinopithecus_brelichi:0.018366353498734,Rhinopithecus_avunculus:0.03580120169119194):0.006037634805500236,Rhinopithecus_roxellana:0.02759729933986832):0.004154227252931575):0.04335699887028843):0.010066549339814657):0.017195776302707078):0.0058151698727408885,(((Trachypithecus_phayrei:0.03536743230210759,((Trachypithecus_francoisi:0.009032399922331236,Trachypithecus_poliocephalus:0.004752736794745138):0.018948264213112576,Trachypithecus_laotum:0.016675510074480704):0.009804299424135299):0.00834828756581052,((Trachypithecus_germaini:0.009702050941237272,(Trachypithecus_auratus:0.0102433235445612,Trachypithecus_cristatus:0.01219796203968454):5.372012949833582E-4):0.01790067631788883,(Trachypithecus_obscurus:0.0170409852292841,Trachypithecus_barbei:0.02147372267837161):0.0086355699669447):0.009547550713999597):0.04519548321737776,(Presbytis_thomasi:0.05370873081113686,(((Presbytis_hosei:0.049396839441937124,(Presbytis_frontata:0.04772929227694189,Presbytis_chrysomelas:0.046787201277576074):0.01183536994247747):0.008474766073830686,((Presbytis_siberu:0.015599898144765367,Presbytis_potenziani:0.00914101776805254):0.008867021025656785,(((Presbytis_melalophos:0.013676398055127437,(Presbytis_bicolor:0.021849178347959353,Presbytis_rubicunda:0.012260090214561592):0.0016951908312316522):0.0026472710538874566,Presbytis_sumatranu:0.02428723090791557):0.008730675647731612,(Presbytis_mitrata:0.018480881515662406,(Presbytis_fredericae:0.011885057070940341,Presbytis_comata:0.010676967898164691):0.007631912142309405):0.0037450788781832056):0.007047355696521096):0.03720335544966924):0.00717602817792895,Presbytis_femoralis:0.044848661288532155):0.00548132177035332):0.04687713275563554):0.012473389705932925):0.026187786046941608):0.05562386465620239,((((Theropithecus_gelada:0.04558595026569634,((((Papio_ursinus:0.023921015879089458,Papio_cynocephalus:0.010434554819230724):0.006062657757107015,(Papio_anubis:0.012086044559357578,Papio_papio:0.019988074322456528):0.0015864158473404277):0.0017745352136731007,Papio_hamadryas:0.019144420236709703):0.011602694782106071,Rungwecebus_kipunji:0.01375235332414726):0.02454281650540413):0.009344555555175327,(Lophocebus_aterrimus:0.014941365497973869,Lophocebus_albigena:0.027339719552785025):0.030699495290422485):0.04038142291639091,((Miopithecus_talapoin:0.04397824364413472,Miopithecus_ogouensis:0.04327767956968409):0.040620228955065785,(((Mandrillus_sphinx:0.027446792734775682,Cercocebus_torquatus:0.04138412752111148):0.015185334927495742,(Mandrillus_leucophaeus:0.017195751043389773,(Cercocebus_agilis:7.092580193048814E-4,Cercocebus_galeritus:0.008077672918737067):0.034425656567993235):0.011770291252231946):0.051118853236231,(Macaca_sylvanus:0.060222513869465866,((((((Macaca_tonkeana:0.024518186067123193,Macaca_nigra:0.02087341039356455):0.012095084413470292,Macaca_nigrescens:0.020039638155084694):0.00907190406315661,Macaca_hecki:0.017579344791795348):0.0029970891533526167,(Macaca_maura:0.03420456001766592,(Macaca_brunnescens:0.018679050630638516,Macaca_ochreata:0.016131624792055044):0.011627005112752054):0.004704372915675492):0.013864931250007761,(Macaca_silenus:0.03916847026876402,(Macaca_pagensis:0.03538042510473999,(Macaca_nemestrina:0.014997349383625513,(Macaca_leonina:0.019486836881571656,Macaca_siberu:0.011507962042011655):0.013834617495545931):0.007205072740227747):0.007738640793824358):0.008210579672638385):0.012696720015731855,(((Macaca_sinica:0.03583739839825384,(Macaca_thibetana:0.009521154913597618,Macaca_assamensis:0.007138215345214638):0.020638672520795653):0.008198065085477602,(Macaca_radiata:0.016142748921443184,Macaca_munzala:0.025117935773349365):0.015617871469517652):0.008478049359333828,((Macaca_mulatta:0.024580718258271295,(Macaca_fuscata:0.0174601731219155,Macaca_cyclopis:0.00920388370587577):0.007673944360460094):0.012557359012168812,(Macaca_fascicularis:0.041396043051151854,Macaca_arctoides:0.03468454926401876):0.005994644002364824):0.008437299086751282):0.014230039579533282):0.012023970825667973):0.035286348459816486):0.005069371593433014):0.004871206906273184):0.01490142593097088,(Allenopithecus_nigroviridis:0.07420699886009707,(Erythrocebus_patas:0.10509133116109631,(((Cercopithecus_neglectus:0.09145044031381466,((Cercopithecus_diana:0.046965091039157913,(Cercopithecus_wolfi:0.0093745224920746,Cercopithecus_pogonias:6.369498248948169E-6):0.004860181204904834):0.003023261761758378,Cercopithecus_mona:0.037361986270014524):0.00825440288385293):0.01641105940387555,(Cercopithecus_campbelli:6.3694982488371465E-6,(Cercopithecus_hamlyni:0.08784739616384385,((((Chlorocebus_aethiops:0.013756255349056667,Chlorocebus_tantalus:0.014887486297977426):0.0032328350445458565,Chlorocebus_pygerythrus:0.017815790611892424):0.012816729052840592,Chlorocebus_sabaeus:0.02865383852475878):0.005707252727142564,Cercopithecus_solatus:0.03751394241977868):0.021223938770653206):0.010029664338211397):0.004192566893078453):0.004197162973344826,(((Cercopithecus_lhoesti:0.030701583743591554,Cercopithecus_preussi:0.02955459005327088):0.014848684162653392,((Cercopithecus_erythrotis:0.017282361403120894,Cercopithecus_cephus:0.011632275807630155):0.008504216719005475,((Cercopithecus_petaurista:0.024016364054107397,Cercopithecus_erythrogaster:0.009478598817466266):0.008902805490864862,Cercopithecus_ascanius:0.007434917382241446):0.005642611255636032):0.011483819844366083):0.033182580240389115,(Cercopithecus_mitis:0.03756310576236477,Cercopithecus_nictitans:0.028221561315006638):0.03224688987987834):0.013115307681064836):0.007793561908358004):0.016611873860871262):0.014999920513611764):0.04069093327031714):0.06855944378952816,((((Hoolock_leuconedys:0.010399326699089939,Hoolock_hoolock:0.010124832695397101):0.0398134264031591,((Hylobates_pileatus:0.027866419495685912,(((Hylobates_agilis:0.00899026904098732,Hylobates_albibarbis:0.014731716546166118):0.018826845584192675,((Hylobates_abbotti:0.008617678858274913,(Hylobates_funereus:0.01337309381694718,Hylobates_muelleri:0.008419260625478486):0.005422237088256288):0.009881984520503151,Hylobates_lar:0.027384800654991492):0.0030157773303041147):0.003032906421567172,(Hylobates_klossii:0.024087726742104576,Hylobates_moloch:0.02396631716003328):0.003784704315734966):0.008114014645180023):0.02238500507402963,Symphalangus_syndactylus:0.042237533071624034):0.003900950557881866):0.005539139183679875,(((Nomascus_gabriellae:0.012411381065280613,(Nomascus_leucogenys:0.0029584794956563076,Nomascus_siki:0.0030585517067944767):0.007668186666047072):0.00704681181645328,Nomascus_concolor:0.01249973209942512):0.00696371096673154,(Nomascus_nasutus:0.028529024461034003,Nomascus_hainanus:0.02913463014428852):0.012000259156448534):0.03260157742653291):0.06554473667712823,((Gorilla_gorilla:0.05915646833265098,(Homo_sapiens:0.05073373019186633,(Pan_paniscus:0.016063259587431822,Pan_troglodytes:0.01847105134745164):0.026630394104677935):0.014751907353944826):0.03337827900407708,(Pongo_abelii:0.0286228281867017,Pongo_pygmaeus:0.03457865346329858):0.07164526862167064):0.02748831723133982):0.04084720987745283):0.09470750665435901):0.137800830304458):0.03255285079645037):0.03298751213151424):0.027936669114151793,((Tupaia_minor:0.09832297797335532,Tupaia_glis:0.07507712125406552):0.2865604364444155,Lagomorpha:0.2504556359473566):0.0279366691141518);

7. RAxML phylogram based on 69 nuclear genes.

(((Cynocephalus_volans:0.0112084687476669,Galeopterus_variegatus:0.011535328161343256):0.07049237970868688,(((((Perodicticus_potto:0.01758920018785115,Arctocebus_calabarensis:0.020914354707966598):0.014314100784594996,(Loris_tardigradus:0.01822598910827472,((Nycticebus_bengalensis:7.765741936278103E-4,Nycticebus_coucang:7.320314833842712E-4):0.0041365177667835595,Nycticebus_pygmaeus:0.0053510762922678035):0.011111501739043711):0.015091613962438105):0.0021326994989384063,(Euoticus_elegantulus:0.017991697746462643,((((Otolemur_crassicaudatus:0.005403183549621854,Otolemur_garnettii:0.003628482416651785):0.006909544857273492,(Galago_moholi:7.386301341749668E-4,(Galago_senegalensis:6.291577185854047E-4,Galago_matschiei:0.0019381764173426463):1.8082024282883857E-6):0.012214551963813325):0.0030667271599575663,Galago_thomasi:0.016184484797226367):0.001611657359592955,Galago_demidoff:0.008499432764577902):0.01201580034807423):0.004291017490751142):0.06140224635121094,(Daubentonia_madagascariensis:0.03462171960835875,(((Varecia_variegata:6.861096627087393E-4,Varecia_rubra:8.025837760683163E-4):0.015146194947420949,((Lemur_catta:0.005887853988259695,(Prolemur_simus:0.006046333392864284,(Hapalemur_occidentalis:8.985061756292823E-4,Hapalemur_griseus:5.404259756351393E-4):0.0051767832987070495):9.89802217321728E-4):0.009945000542605775,(Eulemur_rubriventer:0.00600058734264608,((Eulemur_macaco:8.814093096477238E-4,Eulemur_flavifrons:7.456651532696812E-4):0.004163042605945078,((Eulemur_mongoz:0.004163293508379656,((Eulemur_sanfordi:0.0010910916839343143,Eulemur_albifrons:8.044298339306977E-4):0.0012937405238750421,((Eulemur_rufus:0.0014433678138796868,Eulemur_collaris:0.0016622575193378508):6.591001578588052E-4,Eulemur_fulvus:0.001282307339559835):4.820787449912045E-4):0.001115684646305709):4.8347891887745575E-4,Eulemur_coronatus:0.004330689979370278):6.406443336585133E-4):0.001563334174646408):0.008535167356445938):0.002550639351754147):0.0091691754809137,((((((Microcebus_ravelobensis:0.006969646916739153,((Microcebus_simmonsi:0.0041784922081195,(Microcebus_rufus:0.0018748604707567174,(Microcebus_spDWW2010aManantantel:0.004134277450335927,((Microcebus_berthae:0.0023114984618317624,Microcebus_myoxinus:3.6704672301049457E-4):0.0015710136260468355,Microcebus_spDWW2010aMarolambo:0.0022338023268900775):3.8249971593554144E-4):8.067584452395582E-4):0.0011434491391527635):7.722452909130206E-4,(Microcebus_tavaratra:0.004179984840348955,((Microcebus_mittermeieri:3.7323942357636586E-4,Microcebus_lehilahytsara:7.538977737149011E-4):0.0024824631468844394,(Microcebus_sambiranensis:0.003185161758668953,Microcebus_arnholdi:0.0032483248182197944):0.001133318082027851):0.002048640537060331):7.500786985515373E-4):0.00162732398429849):0.001203742578893885,(Microcebus_griseorufus:0.003437618136724968,Microcebus_murinus:0.003913324843717286):0.0033434873216275363):0.00624415542986026,(Mirza_coquereli:0.0029286983126397825,Mirza_zaza:8.300435958710772E-4):0.016848727185489965):0.011875548167148031,(Cheirogaleus_sibreei:0.006219034997948569,(Cheirogaleus_crossleyi:0.004807768063589873,(Cheirogaleus_medius:0.007155748466460193,Cheirogaleus_major:0.007646387829793544):9.883424502805382E-4):0.0014225929068121534):0.00786102353778409):0.006832616664125546,(((Lepilemur_dorsalis:0.0023258269957038835,(Lepilemur_septentrionalis:0.0013899285586812804,Lepilemur_ankaranensis:0.0011416829438977705):5.440164753565124E-4):0.003717711891385006,Lepilemur_ruficaudatus:0.004953137724614143):0.0026114670069607437,Lepilemur_jamesorum:0.006866533971578076):0.021297431083313306):0.0032072223227973262,(Lepilemur_edwardsi:0.02150082288021657,(Avahi_laniger:0.01738861155183591,((Propithecus_edwardsi:0.0018184033590180892,Propithecus_diadema:0.001796999112337272):0.003710619341520477,(Propithecus_tattersalli:0.002474280426558284,(Propithecus_verreauxi:0.0025692953734649526,Propithecus_coquereli:0.0014289932929904547):4.3042416008490236E-4):0.0027807280804934):0.005840973438212244):0.009466426988491317):0.004278115724024617):0.00131814807111294):0.016910613692913357):0.005566319902718109):0.024033820816733165,((Tarsius_tarsier:0.02917647461781031,(Tarsius_syrichta:0.009625997314529094,Tarsius_bancanus:0.007876690053352786):0.011401392688730666):0.08214264272378433,(((((Pithecia_pithecia:0.003231304277705932,Pithecia_irrorata:0.0026993499972739754):0.00758899426687587,((Cacajao_calvus:0.0020180565606640055,Cacajao_melanocephalus:0.0031405815899559486):0.0031333099832567823,(Chiropotes_chiropotes:8.867668651448002E-4,Chiropotes_israelita:0.001970719894815176):0.005140359006364281):0.005456671256437645):0.005870514993508075,(Callicebus_torquatus:0.0038522113001246416,((Callicebus_nigrifrons:0.003345780111500607,(Callicebus_personatus:0.0023116563481528907,Callicebus_coimbrai:0.002064157143443296):0.0014722769071519037):0.003466073616433668,((Callicebus_donacophilus:4.7701394060678526E-4,Callicebus_caligatus:0.0023356018943749834):0.0027669266059225373,(Callicebus_cupreus:0.001136674873547433,(Callicebus_moloch:0.002490306981695445,Callicebus_brunneus:0.00618338193757334):4.4585405688729596E-4):0.0011106215769187133):0.0037583522607839703):2.4942169389358115E-4):0.011335755764945221):0.0033180825243416256,((((((Alouatta_pigra:7.176339532778131E-4,Alouatta_sara:0.0020042706095683704):6.515541868334918E-4,(Alouatta_belzebul:0.0026153174797328704,Alouatta_caraya:0.0032825861024883274):3.824900462277847E-4):6.503839373803444E-4,Alouatta_seniculus:0.00387374946580335):2.9909807562039648E-5,Alouatta_palliata:0.0035712628618112807):0.010978971454930847,(((Ateles_chamek:9.322041189190888E-4,Ateles_paniscus:7.665196594053569E-4):0.0015476272924522527,(((Ateles_belzebuth:6.761400176535648E-4,Ateles_geoffroyi:3.0281474883558923E-4):6.320732892221015E-4,Ateles_hybridus:0.0020073389373297668):2.4857462695990207E-4,(Saguinus_tripartitus:0.004095431907692404,Ateles_fusciceps:0.0012129906756669917):8.000010611730612E-5):8.107708131878111E-4):0.006346566809249027,((Brachyteles_arachnoides:0.0017056807236597027,Brachyteles_hypoxanthus:0.003157806259257462):0.0065370712387168295,(Lagothrix_cana:1.9160712327231932E-4,Lagothrix_lagotricha:7.626150621251049E-4):0.007265635108165358):9.220426633786138E-4):0.003011675116041418):0.005165182264118606,(((Saimiri_boliviensis:0.0016637676855355,(Saimiri_ustus:0.0029551753817444737,(Saimiri_oerstedii:6.781996941830348E-4,Saimiri_sciureus:4.659442066578534E-4):3.727220269008624E-4):7.383190695231523E-4):0.01620793799279363,((Cebus_apella:0.0019076321476641234,(Cebus_robustus:0.0023022741707275363,Cebus_xanthosternos:3.768800588917487E-4):1.673189751520976E-4):0.002990534452660648,(Cebus_albifrons:0.0024727632806206823,(Cebus_capucinus:0.0011558618444198476,(Cebus_kaapori:6.986300387680777E-4,Cebus_olivaceus:4.6530930948382454E-4):7.427977840333555E-4):1.713124375588293E-4):0.00328396487914856):0.010292660073860233):0.002468199609763938,(((((Leontopithecus_chrysopygus:5.30470287283924E-4,Leontopithecus_rosalia:2.556912569742775E-4):6.182007389313182E-4,Leontopithecus_chrysomelas:5.807051342244796E-4):0.013176912671704039,(Callimico_goeldii:0.010152517926841853,(((Callithrix_mauesi:0.003135817237090749,(Callithrix_humeralifera:0.002708106183008513,Callithrix_argentata:0.0013616772515158415):8.21660935124896E-4):0.0017090543796864088,(Callithrix_humilis:0.0012820503596291388,Callithrix_pygmaea:0.0014395359961497933):0.0028656502499564973):8.661100096685748E-4,(Callithrix_aurita:0.003780717150317109,((Callithrix_jacchus:5.558829017565103E-4,Callithrix_penicillata:5.863598330632103E-4):2.8144972834132087E-4,(Callithrix_kuhlii:0.0025175855290731908,Callithrix_geoffroyi:6.335113053367591E-4):5.7669064914805945E-5):0.0013432008214039615):0.0015333720472052448):0.007132329961504258):0.002164513414051855):8.566216366984181E-4,(Saguinus_fuscicollis:0.006074443550258601,(((Saguinus_geoffroyi:5.096056470560717E-4,Saguinus_oedipus:5.292935950780009E-4):0.002635183780852063,((Saguinus_bicolor:5.659443943473175E-4,Saguinus_martinsi:0.002161687853399985):0.0016041960199551508,Saguinus_midas:0.0019433721559286576):0.0024967097990218345):0.0011393693295166374,(Saguinus_imperator:0.0035138176651943742,(Saguinus_mystax:9.016329863821837E-4,Saguinus_labiatus:0.0018123149744742606):0.0011495102487792397):0.002400129523459249):8.397674867446914E-4):0.0064540392998949925):0.007038414105271368,((Aotus_griseimembra:0.0016602454590937676,Aotus_trivirgatus:0.0021011499588521054):0.0013770186595184475,((Aotus_infulatus:0.0013051149123918804,Aotus_azarae:4.3041651925224134E-4):0.0035738704305576796,Aotus_nancymaae:0.0011782228093938918):0.0011741714768853106):0.011254388570839935):4.5464795436830907E-4):0.0032445681622423617):0.001262401415334291):0.023329439326382595,((((Pongo_pygmaeus:4.700654622731282E-4,Pongo_abelii:8.058686775596069E-4):0.008749029883523302,((Gorilla_gorilla:9.282350412599116E-4,Gorilla_beringei:1.8082024282883857E-6):0.003952887766148244,((Pan_troglodytes:6.564510615134633E-4,Pan_paniscus:0.0013361132968038592):0.0033799042084449726,Homo_sapiens:0.00486592195503649):9.046252507284025E-4):0.005892148707056899):0.001581686022942147,((((Nomascus_concolor:7.306795662907484E-4,Nomascus_gabriellae:3.7807101084338934E-4):1.760049350205961E-4,(Nomascus_siki:2.8375867436980506E-4,Nomascus_leucogenys:3.537241723800544E-4):4.887581360852877E-4):0.0029709210400843122,((Hylobates_moloch:1.8082024283161413E-6,((Hylobates_agilis:0.0013654951190612274,Hylobates_muelleri:8.651811286272404E-4):3.9910245350580076E-4,Hylobates_lar:0.001817029618634558):0.0017796357058653955):0.001477247572511109,Symphalangus_syndactylus:0.00369657775306495):3.5005651989930553E-4):0.006302427681266598,Hoolock_hoolock:0.0018188022568063067):0.0030183994707527473):0.0063348904536244255,(((((Colobus_angolensis:0.0018828350192628407,(Colobus_polykomos:0.0015805840128025883,Colobus_guereza:0.0016364495756273356):4.2824025114271413E-4):0.0038605272976307003,Piliocolobus_badius:0.005881047352818719):3.4378479091573544E-4,Procolobus_verus:0.006650259118695573):0.0015166682112702778,((Presbytis_comata:2.467376297075885E-4,Presbytis_melalophos:7.941888026169741E-4):0.004494513932478905,((Rhinopithecus_brelichi:0.004235225264324821,((Pygathrix_nigripes:0.0012286233065347818,(Pygathrix_nemaeus:5.227019116439136E-4,Pygathrix_cinerea:7.318514626057648E-4):5.435993573287556E-4):0.0027268844100714063,((Nasalis_larvatus:0.0019176613999175585,Simias_concolor:0.002200627568127389):0.0023114069354046585,(Rhinopithecus_roxellana:1.8082024282883857E-6,Rhinopithecus_avunculus:4.777139720071044E-4):0.0028646393557326955):2.5825015568747833E-4):1.9697104314519986E-4):7.198926220853274E-4,((Trachypithecus_vetulus:0.0012121756626650715,(Semnopithecus_hector:0.0011869252460642576,Semnopithecus_entellus:5.644998213228114E-4):7.271557770220372E-4):0.0010243166007885551,(Trachypithecus_johnii:0.004306797295912046,((Trachypithecus_hatinhensis:3.4053880733925235E-4,(Trachypithecus_delacouri:2.7123899487865866E-4,Trachypithecus_francoisi:5.818779117924833E-4):9.687396258922787E-5):9.05656443904862E-4,((Trachypithecus_phayrei:0.0010220829783453012,Trachypithecus_obscurus:5.964152845490123E-4):3.609649614389199E-4,(Rhinopithecus_bieti:0.003297090801286373,(Trachypithecus_auratus:3.9453345164625064E-4,Trachypithecus_cristatus:1.8082024283161413E-6):1.8082024282883857E-6):6.36532063977846E-4):1.8054283107257763E-4):8.064028890534813E-4):1.8082024282883857E-6):0.0029926237807927913):2.594924220466477E-4):0.001776436113515023):0.0034440370367256334,((((Theropithecus_gelada:0.0016607549868685179,((Lophocebus_albigena:1.2808081031584484E-4,Lophocebus_aterrimus:1.3264719335298603E-4):0.0018929690722909731,(Papio_papio:7.408778077213074E-4,(Papio_hamadryas:0.0010280797503252614,Papio_anubis:4.0602662114180843E-4):2.578179160790395E-4):0.0015842751039375491):5.493896485682104E-4):9.65531450357604E-4,(Papio_cynocephalus:1.8082024282883857E-6,(((Cercocebus_agilis:0.0011928722559005345,Cercocebus_chrysogaster:1.0220548875378155E-4):0.0016122410719077718,(Cercocebus_galeritus:0.0028013098934118186,(Cercocebus_atys:0.0023751637244871504,Cercocebus_torquatus:1.8082024282883857E-6):9.36891814294627E-4):0.0017550956826046793):0.001089094039527283,(Mandrillus_leucophaeus:0.0012070270807198469,Mandrillus_sphinx:5.289136093182267E-4):0.002037657248252722):5.126328903610755E-4):5.38092950412572E-4):7.355496609737777E-4,(Macaca_sylvanus:0.0026725483000429873,(((((Macaca_nigra:9.708845168714952E-4,Macaca_nigrescens:2.2945866912854607E-4):4.153474922218847E-4,((Macaca_brunnescens:2.669152201079106E-4,Macaca_ochreata:1.8082024282883857E-6):5.754872274841838E-4,Macaca_hecki:6.86649917430282E-4):1.8082024282883857E-6):1.3281394782138634E-4,(Macaca_maura:5.393824916779533E-4,Macaca_tonkeana:7.896522275727125E-4):1.4517106369449362E-4):7.380044946019848E-4,((Macaca_nemestrina:8.884824455583917E-4,Macaca_silenus:9.47552307875521E-4):5.989542323575625E-4,Macaca_siberu:0.0013572088734100651):2.909178434252202E-4):6.209991124084824E-4,((((Macaca_arctoides:5.366263308195229E-4,(Macaca_radiata:4.523930783408847E-4,Macaca_assamensis:0.0010640443588865178):2.5054472003688266E-4):4.4825929126168784E-4,Macaca_sinica:6.517807933059672E-4):3.1646423638004295E-4,Macaca_thibetana:9.525090674826764E-4):5.011288538859793E-4,(Macaca_fascicularis:0.0018769425713506815,((Macaca_mulatta:9.92695512277153E-4,Macaca_fuscata:0.0011455630958929208):1.38657412778187E-4,Macaca_cyclopis:0.0011671897535041054):5.1488636783803E-4):2.186212973161461E-4):2.05859881840742E-4):4.8517098847133955E-4):0.0019164805373019789):0.0019478368077065489,((Miopithecus_ogouensis:0.004147748193550016,(Cercopithecus_hamlyni:0.0027367655200756236,((Cercopithecus_diana:0.0027385932863019935,((Cercopithecus_mitis:4.111084254293518E-4,Cercopithecus_albogularis:6.021171774549527E-4):0.0015185952752611032,(((Cercopithecus_ascanius:0.001145339117356925,(Cercopithecus_nictitans:0.004665601576543998,Chlorocebus_pygerythrus:0.0018504433419725963):1.8082024282883857E-6):1.6204970660971263E-4,Cercopithecus_cephus:0.0010023233117737451):2.680257960899768E-4,Cercopithecus_petaurista:0.0017136849795221232):2.5238107540881405E-4):8.839883578125596E-4):2.6617480548007943E-4,((Cercopithecus_mona:0.002236935423907349,Cercopithecus_wolfi:8.273682380527947E-4):0.0011713323508726892,Cercopithecus_neglectus:0.0032516783238113467):3.3273375285169937E-4):8.258193959842508E-5):5.705542528554597E-4):2.744602549761832E-4,(((Chlorocebus_cynosuros:4.087443651771827E-4,((Chlorocebus_sabaeus:0.0010924995843087926,Chlorocebus_tantalus:1.8082024282883857E-6):1.8082024282883857E-6,Chlorocebus_aethiops:9.578907901102718E-4):1.5099819183911345E-4):0.0018442194761777442,(Erythrocebus_patas:0.0029246640523659106,((Cercopithecus_solatus:0.0015297164068497904,Cercopithecus_preussi:1.8082024282883857E-6):1.8082024282883857E-6,Cercopithecus_lhoesti:1.8082024282883857E-6):0.0027173858630228365):2.705187398536202E-4):0.001420090182843392,(Allenopithecus_nigroviridis:0.002057466641697092,Miopithecus_talapoin:1.8082024282883857E-6):0.0029757340404522603):3.612213732721026E-4):0.0016106505864167397):0.003258117902067703):0.01258681382411117):0.012934976346544419):0.043581511694955694):0.005971278402398278):0.008525871203316382):0.0035843198408427256,(Lagomorpha:0.16861137145791333,(Tupaia_glis:0.012249895386966192,Tupaia_minor:0.01321686260920768):0.11073681966179388):0.003584319840842737);
