# Supplementary material for: Macroevolutionary Dynamics and Historical Biogeography of Primate Diversification Inferred from a Species Supermatrix
Source: PLoS One. 2012 Nov 16;7(11):e49521. doi: 10.1371/journal.pone.0049521 (PMC3500307; doi:10.1371/journal.pone.0049521)
Supplement: Text S3 — Fourteen minimum and 14 maximum calibrations that were employed in primate timetree analyses with mcmctree [140], [141] . Minimum calibrations were based on the oldest crown fossil belong to each clade. Maximum calibrations were based on stratigraphic bounding (two chronologic units), phylogenetic bracketing (two outgroups), and phylogenetic uncertainty [18], [22]. (DOCX) [file pone.0049521.s009.docx]

Text S3. Fourteen minimum and 14 maximum calibrations that were employed in primate timetree analyses with mcmctree [1,2]. Minimum calibrations were based on the oldest crown fossil belong to each clade. Maximum calibrations were based on stratigraphic bounding (two chronologic units), phylogenetic bracketing (two outgroups), and phylogenetic uncertainty as in Meredith et al. [3].

Calibration 1. Lorisiformes. Minimum = 37.1 Ma; maximum = 56 Ma. The minimum age is based on *Saharagalago misrensis*, which is the oldest crown lorisiform. *S. misrensis* is known from the Bartonian and was assigned to Galagidae by Seiffert et al. [4]; *Karanisia* emerged as a crown lorisid in Seiffert et al.'s [4] analysis, although these authors also allow for the possibility of stem lorisid or stem lorisiform status for *Karanisia*. The maximum age is based on stratigraphic bounding (base of Ypresian = 55.8 +/- 0.2 = 56 Ma).

Calibration 2. Haplorhini. Minimum = 37.1 Ma; maximum = 58.9 Ma. We used the top of the middle Eocene (37.1 Ma) as a minimum for crown Haplorhini based on the occurrence of numerous eosimiids from the middle Eocene [5] including *Tarsius eocaenus* [6,7]. *Altiatlasius* is known from the late Paleocene and is a stem anthropoid in some cladistic analyses [8,9], but not in Bloch et al. [10]. *Anthrasimias gujaratensis* (54-55 Ma, Sparnacian) is another putative stem anthropoid [8,11], although Rose et al. [9] suggest that *Anthrasimias gujaratensis* may be a junior synonym of the adapoid *Marcgodinotius indicus*. *Algeripithecus* is younger (> 45 Ma) and has been referred to Anthropoidea [12]. However, recent discoveries of more complete specimens of *Algeripithecus* suggest that it is not an anthropoid [5]. Instead, it may belong to Azibiidae, which has uncertain phylogenetic affinities (e.g., adapiform, early euprimate, plesiadapiform, or even nonprimate) [5]. The maximum age for Haplorhini is based on the possibility that *Altiatlasius* is a stem anthropoid [8,9].

Calibration 3. Simiiformes (= Anthropoidea). Minimum = 28.3 Ma; maximum = 56 Ma. The minimum age for Anthropoidea is based on *Aegyptopithecus* (early Oligocene~Rupelian), which is a stem catarrhine in cladistic analyses that were either unconstrained or constrained with a molecular backbone [13]. The oldest stem platyrrhine is *Branisella* [13] from the late Oligocene of Bolivia (Salla Beds, Chron 8, age = 25.82-27.02 Ma according to Kay et al. [13]). The maximum age for Anthropoidea is based on *Algeripithecus minutus*, which Seiffert et al. [12] recovered in a polytomy with catarrhines, platyrrhines, Parapithecoidea, and Proteopithecidae. This establishes a maximum of 56.0 for the base of Anthropoidea if we allow for phylogenetic uncertainty, i.e., that *Algeripithecus* is either a crown anthropoid or one of the first two outgroups. In contrast, Tabuce et al. [14] recovered *Algeripithecus* as a stem strepsirrhine.

Calibration 4. Catarrhini. Minimum = 20.55 Ma; maximum = 37.3 Ma. *Afropithecus turkanensis*, previously *Morotopithecus bishopi* [15], is dated at 20.6 +/- 0.5 = 20.55 based on overlying lava [16] and is the oldest crown-group catarrhine. It is a stem hominoid [17] or even a crown hominoid [16]. The oldest cercopithecoid is *Victoriapithecus* [18] at ~19 Ma. The maximum age is based on the oligopithecid *Catopitheus*, which is sometimes regarded as a stem catarrhine that is a member of the second outgroup to crown catarrhines (fig 1a in Seiffert and Simons [19]). The age of *Catopithecus* is 34.8-33.7 (Chron C13r) based on Seiffert [20], which is mostly within the Priobonian. We used the base of the Priabonian (37.2 +/- 0.1 = 37.3) as the maximum age for the base of Catarrhini.

Calibration 5. Strepsirrhini. Minimum = 37.1 Ma; maximum = 56 Ma. The minimum age for Strepsirrhini is based on *Saharagalago* and *Karansia*. *Saharagalago* is known from the Bartonian [4] and is assigned to Galagidae; *Karanisia* emerged as a crown lorisid in Seiffert et al.'s [4] analysis, although these authors also allow for the possibility of stem lorisid or stem lorisiform status for *Karanisia*. The maximum age is based on early Eocene adapiforms, which may be included in the first or second outgroup to crown Strepsirrhini [21,22].

Calibration 6. Hominoidea. Minimum = 11.608; maximum = 28.5. *Sivapithecus* is the oldest crown hominoid is Finarelli and Clyde's [17] cladistic and stratocladistic analyses. The maximum age for Hominoidea is based on stratigraphic bounding.

Calibration 7. *Aotus* to *Saguinus* + *Leontopithecus* + *Callimico* + *Callithrix.* Minimum = 11.8 Ma; maximum = 28.5 Ma. The minimum age is based on *Aotus dindensis*, which is the oldest member of the crown clade that includes *Aotus* and its sister lineage (i.e., subfamily Callitrichinae of Hartwig and Meldrum [23] including *Saguinus, Leontopithecus, Callithrix, Callimico, Mohanamico*). *A. dindensis* is known from the Laventan (middle Miocene) [24], which has an age that ranges from 13.5-11.8 Ma [25]. Kay [26] takes a different view and regards *A. dindensis* as a junior synonym of *Mohanamico*, but most authors recognize *A. dindensis* as a distinct taxon, e.g., Hartwig and Meldrum [23] regard *Mohanamico* and *A. dindensis* as separate taxa. If *A. dindensis* is a junior synonym *Mohanamico* it still remains in the crown clade that includes *Aotus* and callitrichines. The maximum age is based on stratigraphic bounding.

Calibration 8. *Theropithecus* to *Papio* + *Lophocebus.* Minimum = 3.5 Ma; maximum = 15.97 Ma. The minimum age is based on the oldest *Theropithecus* fossils, which are from the Pliocene and have a minimum age of ~3.5 Ma [27,28]. The maximum age is based on stratigraphic bounding.

Calibration 9. *Macaca* to other Papionini. Minimum = 5.5 Ma; maximum = 23.03 Ma. The oldest fossil macaque is *Macaca libyca* from the Turolian (late Miocene) of Egypt [29]. *Macaca* fossils are also known from Europe by at least 5.3 Ma [30]. The maximum age is based on stratigraphic bounding.

Calibration 10. Colobinae to Cercopithecinae. Minimum = 8.5 Ma; maximum = 23.03 Ma. The minimum age is based on *Microcolobus turgenensis*, which is known from Ngeringerowa in sub-Saharan Africa. The age of *M. turgenensis* is between 8.5 and 10.5 Ma [31]. Jablonski [29] suggests that *Microcolobus* is closer to colobines than to cercopithecines. The maximum age is based on stratigraphic bounding.

Calibration 11. *Saimiri* to *Cebus*. Minimum = 11.8 Ma; maximum = 28.5 Ma. The minimum age is based on *Neosaimiri fieldsi*, which is known from the Laventan (midde Miocene) [23] and has an age that ranges from 13.5-11.8 Ma [25]. The maximum age is based on stratigraphic bounding.

Calibration 12. Platyrrhini. Minimum = 11.8 Ma; maximum = 37.3 Ma. Numerous crown platyrrhines, including *Neosaimiri*, *Laventiana*, *Nuciruptor*, *Mohanamico*, and *Aotus dindensis* are known from middle Miocene Laventan deposits [23] that have a minimum age of 11.8 Ma. The maximum age is based on Kay et al.’s [13] cladistic analyses, which allow for the possibility (i.e., fig. 21) that *Aegyptopithecus* (early Oligocene) belongs to the second outgroup to crown Platyrrhini.

Calibration 13. *Homo* to *Pan*. Minimum = 5.11 Ma; maximum = 15.97 Ma. The minimum is based on *Ardipithecus ramidus kadabba* from Ethiopia, which has been dated at 5.8-5.2 Ma [32]. The oldest fossils are from the Kuseralee Member of the Sagantole Formation [33]. The upper age for this member of the Sagantole Formation is 5.18 +/- 0.07 Ma = 5.11 Mya [34], which is early Pliocene. *Orrorin tugenensis* and *Sahelanthropus tchadensis* are older, but not all workers agree that these taxa are early hominins [32,35,36]. The maximum age is based on stratigraphic bounding.

Calibration 14. Ponginae to Homininae. Minimum = 11.608 Ma; maximum = 28.5 Ma. *Sivapithecus* is the oldest crown member of Hominidae in Finarelli and Clydes’s [17] cladistic and stratocladistic analyses. The maximum age is based on stratigraphic bounding.

**References**

1. Yang Z (2007) PAML 4: Phylogenetic Analysis by Maximum Likelihood. Mol Biol Evol 24: 1586-1591.

2. Rannala B, Yang Z (2007) Inferring speciation times under an episodic molecular clock. Syst Biol 56: 453-466.

3. Meredith RW, Janecka JE, Gatesy J, Ryder OA, Fisher CA, et al. (2011) Impacts of the Cretaceous Terrestrial Revolution and KPg extinction on mammal diversification. Science 334: 521-524.

4. Seiffert ER, Simons EL, Attia Y (2003) Fossil evidence for an ancient divergence of lorises and galagos. Nature 433: 421-424.

5. Williams BA, Kay RK, Kirk EC (2010) New perspectives on anthropoid origins. Proc Natl Acad Sci U S A 107: 4797-4804.

6. Beard KC, Qi T, Dawson MR, Wang B, Li C (1994) A diverse new primate fauna from middle Eocene fissure-fillings in southeastern China. Nature 368: 604-609.

7. Rossie JB, Ni X, Beard KC (2006) Cranial remains of an Eocene tarsier. Proc Natl Acad Sci U S A 103: 4381-4385.

8. Bajpai S, Kay RF, Williams BA, Pas DP, Kapur VV, et al. (2008) The oldest Asian record of Anthropoidea. Proc Natl Acad Sci U S A 105: 11093-11098.

9. Rose KD, Rana RS, Sahni A, Kumar K, Missiaen P, et al. (2009) Early Eocene primates from Gujarat, India. J Hum Evol 56: 366-404.

10. Bloch JI, Silcox MT, Boyer DM, Sargis EJ (2007) New Paleocene skeletons and the relationship of plesiadapiforms to crown-clade primates. Proc Natl Acad Sci U S A 104: 1159-1164.

11. Prasad GVR (2009) Divergence time estimates of mammals from molecular clocks and fossils: relevance of new fossil finds from India. J Biosci 34: 649-659.

12. Seiffert ER, Simons EL, Clyde WC, Rossie JB, Attia Y, et al. (2005) Basal anthropoids from Egypt and the antiquity of Africa’s higher primate radiation. Science 310: 300-304.

13. Kay RF, Fleagle JG, Mitchell TRT, Colbert M, Bown T, et al. (2008) The anatomy of *Dolichocebus gaimanensis*, a stem platyrrhine monkey from Argentina. *J Hum Evol* 54: 323-382.

14. Tabuce R, Marivaux L, Lebrun R, Adaci M, Bensalah M, et al. (2009) Anthropoid versus strepsirhine status of the African Eocene primates *Algeripithecus* and *Azibius*: craniodental evidence. Proc R Soc B 276: 4087-4094.

15. Harrison T, Andrews P (2009) The anatomy and systematic position of the early Miocene proconsulid from Meswa Bridge, Kenya. J Hum Evol 56: 479-496.

16. Young NM, MacLatchy L (2004) The phylogenetic position of *Morotopithecus*. J Hum Evol 46: 163-184.

17. Finarelli JA, Clyde WC (2004) Reassessing hominoid phylogeny: evaluating congruence in the morphological and temporal data. Paleobiology 30: 614-651.

18. Benefit BR, McCrossin ML (2002) The Victoriapithecidae, Cercopithecoidea. In: Hartwig WC, ed. The primate fossil record. Cambridge, UK: Cambridge Univ. Press. pp. 241-253.

19. Seiffert ER, Simons EL (2001) Astragalar morphology of late Eocene anthropoids from the Fayum Depression (Egypt) and the origin of catarrhine primates. J. Hum. Evol. 41: 577-606.

20. Seiffert ER (2006) Revised age estimates for the later Paleogene mammal faunas of Egypt and Oman. Proc Natl Acad Sci U S A 103: 5000-5005.

21. Seiffert ER, Perry JMG, Simons EL, Boyer DM (2009) Convergent evolution of anthropoid-like adaptations in Eocene adapiform primates. Nature 461: 1118-1122.

22. Seiffert ER, Simons EL, Boyer DM, Perry JMG, Ryan TM, et al. (2010) A fossil primate of uncertain affinities from the earliest late Eocene of Egypt. Proc Natl Acad Sci U S A 107: 9712-9717.

23. Hartwig WC, Meldrum DJ (2002) Miocene platyrrhines of the northern Neotropics. In: Hartwig WC, ed. The primate fossil record. Cambridge, UK: Cambridge Univ. Press. pp. 175-188.

24. Setoguchi T, Rosenberger AL (1987) A fossil owl monkey from La Venta, Colombia. Nature 326: 692-694. The middle Miocene (Laventan) Fitzcarrald Fauna, Amazonian Peru.

25. Antoine PO, Salas-Gismondi R, Baby P, Benammi M, Brusset S, et al. (2007) In: E. Díaz-Martínez E, Rábano I, eds. 4th European meeting on the palaeontology and stratigraphy of Latin America, Cuadernos del Museo Geominero, nº 8. Madrid, España: Instituto Geológico y Minero de, Madrid, España.

26. Kay RF (1990) The phyletic relationships of extant and fossil Pitheciinae (Platyrrhini, Anthropoidea). J Hum Evol 19: 175-208.

27. Leakey MG (1993) Evolution of *Theropithecus* in the Turkana Basin. In: Jablonski NG, ed. *Theropithecus*: the rise and fall of a primate genus. Cambridge: Cambridge Univ Press. pp. 85-123.

28. Steiper ME, Young NM, Sukarna (2004) Genomic data support the hominoid slowdown and an early Oligocene estimate for the hominoid-cercopithecoid divergence. Proc Natl Acad Sci U S A 101: 17021-17026.

29. Jablonski NG, Leakey MG, Kiarie C, Anton M (2002) A new skeleton of *Theropithecus brumpti* (Primates: Cercopithecidae) from Lomekwi, West Turkana, Kenya. J Hum Evol 43: 887-923.

30. Köhler M, Moyà-Solà S, Alba DM (2000) *Macaca* (Primates, Cercopithecidae) from the late Miocene of Spain. J Hum Evol 38: 447-452.

31. Benefit BR, Pickford M (1986) Miocene fossil cercopithecoids from Kenya. Am J Phys Anthropol 69: 441-464.

32. Benton MJ, Donoghue PCJ, Asher RJ (2009) Calibrating and constraining molecular clocks. In: Hedges SB, Kumar S, eds. The timetree of life. Oxford: Oxford Univ Press. pp. 35-86.

33. Haile-Selassie Y (2001) Late Miocene hominids from the Middle Awash, Ethiopia. Nature 412: 178-181.

34. Renne PR, WoldeGabriel G, Hart WK, Heiken G, White TD (1999) Chronostratigraphy of the Miocene-Pliocene Sagantole Formation, Middle Awash Valley, Afar rift, Ethiopia. GSA Bull 111: 869-885.

35. Begun DR (2004) The earliest hominins – is less more? Science 303: 1478-1480.

36. Wolpoff MH, Hawks J, Senut B, Pickford M, Ahern J (2006) An ape or the ape: is the Toumaï cranium TM 266 a hominid? PaleoAnthropology 2006: 36-50.
